# Reactivity of Square Planar Pt(II) Complexes Toward Acidic Moieties as H^+^, M^+^, and [ML]^+^ (M = Ag, Au)

**DOI:** 10.1002/chem.202502370

**Published:** 2025-09-17

**Authors:** Laura Martínez‐Romera, David Campillo, Daniel Escudero, Miguel Baya, Antonio Martín

**Affiliations:** ^1^ Instituto de Síntesis Química y Catálisis Homogénea (ISQCH) CSIC Universidad de Zaragoza C/ Pedro Cerbuna 12 Zaragoza 50009 Spain; ^2^ Department of Chemistry Katholieke Universiteit Leuven Celestijnenlaan 200 f — Box 2404 Leuven 3001 Belgium

**Keywords:** donor‐acceptor systems, hydrogen bond, isolobal relationship, M‐H interactions, polymetallic complexes

## Abstract

The Lewis base (LB) properties of the d^8^ Pt(II) are manifested in the formation of Pt→E species, where E are electrophiles such as acidic hydrogens or metal cations. Thus, the reactions of [Pt(CNC)(dmso)] (CNC = 2,6‐di(phen‐2‐ide)‐pyridine) toward PPh_2_(C_6_H_4_‐*o*‐COOH) or PPh_2_(C_6_H_4_‐*o*‐OH) produce complexes [Pt(CNC‐H){PPh_2_(C_6_H_4_‐*o*‐COO)}] (**1**) and [Pt(CNC‐H){PPh_2_(C_6_H_4_‐*o*‐O)}] (**2**). Their X‐ray structures confirm the proton transfer from the OH at the phosphane to one of the phenylene rings of the CNC ligand. Then, the deprotonated oxygen occupies the vacant site. It is proposed that the transfer starts with a hydrogen bond (Pt→H) which evolves to the final products favored by the planar nature of the CNC ligand. **1** and **2** react with Ag^+^ and [M(PPh_3_)]^+^ (M = Ag, Au) giving rise to complexes without Pt─M bonds, but O─M or O─Ag─O instead. Their structures reveal that the oxygen atoms present in **1** and **2** advantageously compete with the Pt as the donor entity for the incoming acidic metal. Moreover, dinuclear complexes with O─H···O systems structurally analogous to the O─Ag─O have been prepared and characterized. They reinforce the idea of resemblance of the proton and cations such as Ag^+^, Au^+^, [AgL]^+^, and [AuL]^+^ as Lewis acidic moieties, which is consistent with the concept of isolobality.

## Graphical abstract

Despite the fact that Pt(II) in square‐planar complexes exhibits Lewis basic properties toward electrophilic species (E⁺), the presence of oxygen atoms acting as competing Lewis basic sites leads to the formation of Pt─O─E⁺─O─Pt or Pt─O─E⁺ type complexes, in which no direct metal–metal bonds are observed. The formation of analogous species with either H⁺ or [M–L]⁺ fragments can be connected to the concept of isolobality between these electrophilic entities.

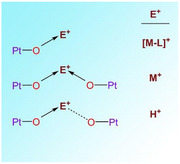

## 1 Introduction

The Lewis base (LB) properties of the d^8^ Pt(II) center in its square planar complexes are well known. This feature arises from the electronic distribution in the d orbitals of the metal and the existence of a filled d_z_2 orbital oriented on the z‐axis direction of the square‐planar environment of the Pt(II).

**Scheme 1:**
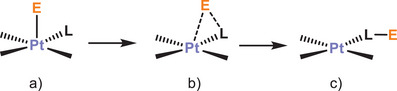
Interactions of electrophiles E with Pt(II) basic centers in square planar complexes.

The basic properties of Pt(II) can be activated toward electrophiles that behave as Lewis acids (E, electrophile, Scheme 1). Thus, the formation of adducts M–E may be the starting point for subsequent reactions in which E is transferred to ligands, involving the breaking and formation of chemical bonds.

In particular, square planar complexes of platinum (II) are known for being ideal precursors in the formation of dative Pt→M bonds with acidic metals (M) such as Ag(I),^[^ ^1^, ^2^, ^3^, ^4^, ^5^, ^6^, ^7^, ^8^, ^9^, ^10^, ^11^, ^12^, ^13^, ^14^, ^15^, ^16^ ^]^ Tl(I),^[^ ^10^, ^17^, ^18^, ^19^, ^20^, ^21^, ^22^, ^23^ ^]^ Au(I),^[^ ^9^, ^23^, ^24^, ^25^, ^26^, ^27^, ^28^, ^29^, ^30^, ^31^ ^]^ Cu(I),^[^ ^32^, ^33^ ^]^ Pb(II),^[^ ^21^, ^34^ ^]^ Cd(II)^[^ ^35^, ^36^ ^]^ or Hg(II),^[^ ^37^, ^38^, ^39^, ^40^ ^]^ giving rise to heteropolymetallic clusters with interesting structural features and varied nuclearities, that in most cases respond to type a) in Scheme 1.

**Figure 1:**
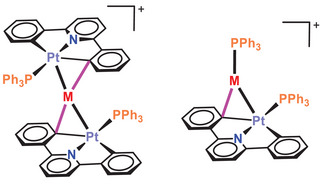
Tri‐ and dinuclear complexes containing the [Pt(CNC)(PPh_3_)] unit showing Pt‐M dative bonds.

Complex [Pt(CNC)(PPh_3_)] (**A**) (CNC = 2,6‐di(phen‐2‐ide)‐pyridine) has been used to prepare di and trinuclear complexes containing Pt─M bonds (M = Ag or Au, see Figure 1).^[^ ^9^, ^41^ ^]^ These Pt─M clusters are interesting not only because of the presence of the intermetallic bonding, but also due to the existence of remarkable interactions between the acidic center and one of the carbon atoms of the CNC ligands, resembling structures of type b) in Scheme 1. Such interactions cause a perceptible distortion on the geometry of the “Pt(CNC)” fragments and can be regarded as snapshots of a frustrated transfer of an E group from the Pt center to M.^[^ ^42^ ^]^ They are possible due to the planar nature of the ligand and its low steric hindrance, that allows the M center to “lean” toward the C*_ipso_* atom. In support of this idea, in most of the heterometallic Pt→M complexes reported so far, the Pt bears more or less bulky ligands deviating from the coordination plane and, as stated above, they display a type a) structure. Only in these CNC complexes and a handful of Pt(II) complexes with other planar chelate complexes such as benzoquinolinate giving rise to planar M(LL’) or M(LL'L‘’) structures, type b) architectures are observed.^[^ ^3^, ^4^, ^5^, ^16^, ^31^, ^43^, ^44^ ^]^

Besides, acidic hydrogen atoms can also activate the LB properties of square planar Pt complexes. Thus, d^8^ square‐planar metallic complexes can react with acids through several possible mechanisms, one of which is oxidative addition leading to M(IV) intermediates that evolve to the final product via a reductive elimination process.^[^ ^45^ ^]^

**Figure 2:**
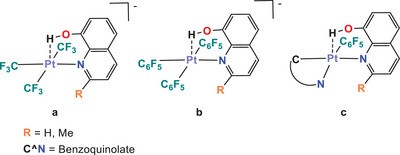
Square planar Pt(II) complexes with O‐H···Pt hydrogen bonding. Pt···H distances are 2.05(9) Å (a), 2.19 Å (b), and 2.09(4) Å (c).

There are also examples in the literature of Pt(II) metallic centers acting as hydrogen‐bond acceptors.^[^ ^46^, ^47^, ^48^, ^49^ ^]^ For example, hydroxyl groups supported by a ligand have a tendency to form that kind of interactions (Figure 2),^[^ ^50^, ^51^, ^52^ ^]^ disposing the hydroxy group perpendicular to the square metallic environment (type a) in Scheme 1). This supports the participation of the filled d_z_2 orbital of the metallic center, that gives rise to formal acid‐base Lewis interactions.

An analogy of the reactivity of basic Pt(II) complexes toward acidic metal centers, such as Ag^+^, Au^+^, [AgL]^+^, or [AuL]^+^, and protons or acidic hydrogen atoms can be made by means of the isolobality concept, since it is established in the literature that the proton and the methyl group are isolobal with [M‐L]^+^ (M = Ag, Au) fragments.^[^ ^53^, ^54^, ^55^, ^56^ ^]^ The concept of isolobality was first proposed by Hoffmann in 1982,^[^ ^57^ ^]^ defining that two fragments are isolobal when their “number, symmetry properties, approximate energy and shape of the frontier orbitals and the number of electrons in them are similar”. This analogy helped to analyze the structure and bonding situation of more complex compounds by the study of less complicated molecules. It was not until 1984 that Stone related this concept to gold species, proposing their isolobal relationship with the proton.^[^ ^58^ ^]^

Thus, in light of this background, we have investigated the reactivity of the solvento starting material [Pt(CNC)(dmso)] (**B**) toward two new phosphane ligands: 2‐(diphenylphosphino)benzoic acid PPh_2_(C_6_H_4_‐*o*‐COOH) and (2‐hydroxyphenyl)diphenylphosphane PPh_2_(C_6_H_4_‐*o*‐OH). These phosphanes have one of their phenyl rings functionalized in the *ortho* position with a group possessing a hydrogen atom with acidic properties, capable of interacting with the metal center and even of triggering a proton transfer process.

Furthermore, if the latter is the case, the resulting square planar Pt(II) complexes shall present additional Lewis base spots other than the metal center. These observations prompt new questions central to our investigation, particularly regarding the potentially competitive interactions between nucleophilic sites toward Lewis acidic H^+^, M^+^, and [M(PPh_3_)]^+^ (M = Ag, Au) fragments. Accordingly, we have examined this reactivity within the broader context of Lewis Pair and Metal‐Only Lewis Pair (MOLP) chemistry.

## 2 Results and Discussion

### 2.1 Syntheses of [Pt(CNC‐H){PPh_2_(C_6_H_4_‐*o*‐COO)}] (1) and [Pt(CNC‐H){PPh_2_(C_6_H_4_‐*o*‐O)}] (2)

**Scheme 2:**
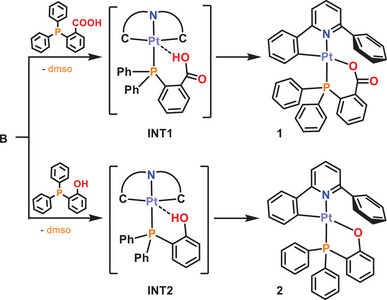
Syntheses of complexes **1** and **2**.

The reactions of [Pt(CNC)(dmso)] (**B**) toward PPh_2_(C_6_H_4_‐*o*‐COOH) or PPh_2_(C_6_H_4_‐*o*‐OH) in CH_2_Cl_2_ for 25 minutes at room temperature were carried out. The P atoms of these ligands are capable of displacing the dmso from the platinum center (**INT1**, **INT2**), and a subsequent proton transfer process occurs, giving rise to complexes with formulas [Pt(CNC‐H){PPh_2_(C_6_H_4_‐*o*‐COO)}] (**1**) and [Pt(CNC‐H){PPh_2_(C_6_H_4_‐*o*‐O)}] (**2**), which are isolated as yellow solids in 81% and 53% yield, respectively (Scheme 2).

**Figure 3:**
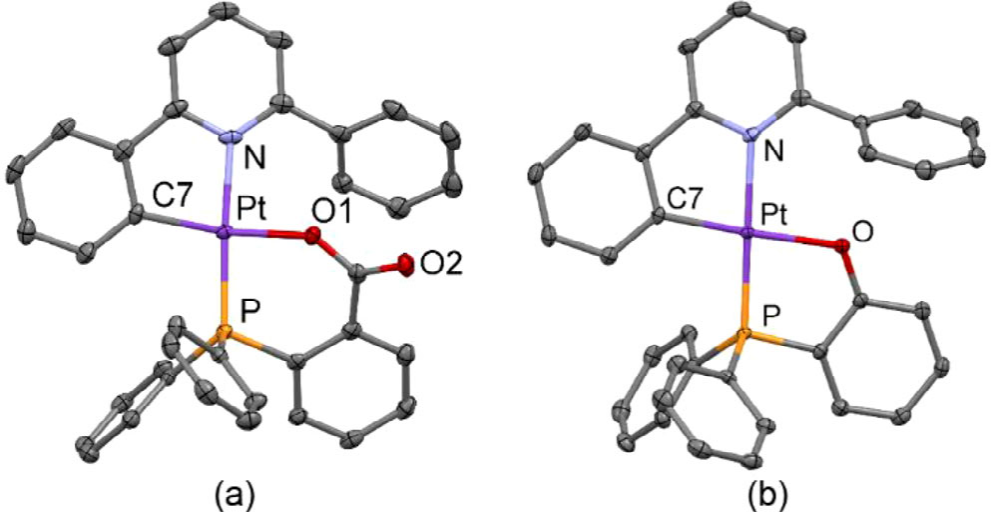
Molecular structures of complexes [Pt(CNC‐H){PPh_2_(C_6_H_4_‐*o*‐COO)}] (**1**, a) and [Pt(CNC‐H){PPh_2_(C_6_H_4_‐*o*‐O)}] (**2**, b).

Crystals of both [Pt(CNC‐H){PPh_2_(C_6_H_4_‐*o*‐COO)}] (**1**) and [Pt(CNC‐H){PPh_2_(C_6_H_4_‐*o*‐O)}] (**2**) could be grown and their structures determined by X‐ray diffraction studies (XRD). Views of them are presented in Figure 3, while some relevant distances and angles are listed in Table S3 (Supporting Information). The structures of **1** and **2** are qualitatively similar and can be described as mononuclear Pt(II) complexes where the phosphane ligand with an oxygen donor atom occupies two coordination sites, thus forming a chelate ring of 5 or 6 members, respectively. A bidentate C^N ligand completes the coordination environment of the metallic centers. These structures confirm the transfer of the hydrogen from the OH group of the phosphane to one of the phenylene rings of the original CNC ligand, which decoordinates from the metal atom. Simultaneously, the deprotonated oxygen occupies the vacant coordination site.

The ^1^H and ^31^P{^1^H} NMR spectra of compounds **1** and **2** at room temperature are consistent with the structures obtained by XRD. The ^1^H spectra endorse the protonation of one of the phenylene rings of the CNC ligand, which now shows a typical pattern for a phenyl ring. On the other hand, the ^31^P{^1^H} NMR spectra confirm the coordination of the phosphorus atom to the platinum center with the presence of singlets along with satellites resulting from the coupling with the active isotopes of platinum (^1^ *J*_P‐Pt_ = 4388 Hz for **1** and 4435 Hz for **2**). Moreover, it is known that the ^31^P{^1^H} NMR signal shift depends on the number of members of the ring in complexes possessing chelated phosphanes. Thus, five‐membered rings typically show more deshielded signals than those with six members.^[^ ^59^ ^]^ This fact is consistently confirmed in the ^31^P{^1^H} NMR signals of **1** and **2**, as the signal for **2** is shifted downfield (20.2 ppm) compared to that of **1** (11.1 ppm).

The mechanism of formation of **1** and **2** can be envisaged through the initial formation of an intermediate in which the phosphane replaces the dmso ligand in **B** and the H atom of the OH fragment establishes a hydrogen bond‐type interaction with the platinum center (**INT1** and **INT2**, Scheme 2). As mentioned in the introduction, this kind of interaction has been detected and fully characterized for some square planar complexes of platinum containing 8‐hydroxyquinoline‐type ligands such as the ones depicted in Figure 2. However, these particular complexes are stable in solution, and transfer of the acidic hydrogen atoms does not take place except for the pentafluorophenyl complex in which, after several hours of reflux in CHCl_3_, the formation of [Pt(C_6_F_5_)_2_(hq^−^)] (hq^−^ = 8‐hydroxyquinolinate) and elimination of C_6_F_5_H is observed.^[^ ^52^ ^]^

**Figure 4:**
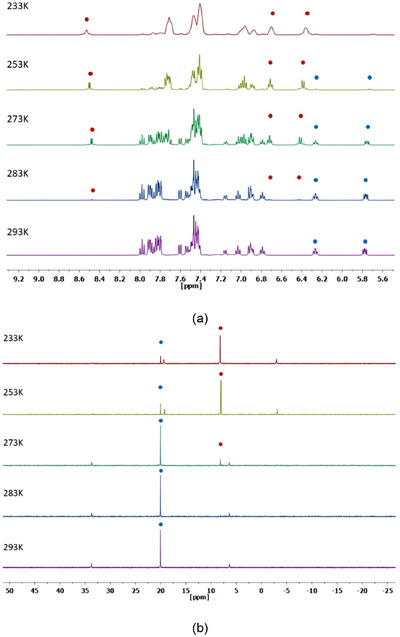
Selected VT NMR spectra (CD_2_Cl_2_) of the reaction of complex [Pt(CNC)(dmso)] (**B**) and (2‐hydroxyphenyl)diphenylphosphane. a) ^1^H spectra. b) ^31^P{^1^H} spectra (blue dots, signals of **2**; red dots; signals of **INT2**). Time lapse of measurements: 90 minutes.

Keeping this in mind, low‐temperature NMR experiments were performed to gain insights into the formation of **1** and **2**. Thus, equimolar amounts of complex [Pt(CNC)(dmso)] (**B**) and the corresponding phosphane ligand were dissolved in CD_2_Cl_2_ at low temperature, and NMR spectra were immediately recorded at 233 K. In the case of the PPh_2_(C_6_H_4_‐*o*‐COOH), both the ^1^H and ^31^P{^1^H} NMR spectra correspond to the final complex **1**, and no further changes are observed in the various measurements performed when increasing the sample temperature up to RT (see Supporting Information, Figures S9, S10). Differently, in the case of the sample with (2‐hydroxyphenyl)diphenylphosphane, both the ^1^H and ^31^P NMR spectra do not correspond to the final product **2** (see Figure 4). The ^31^P{^1^H} NMR spectrum shows a main signal with Pt satellites at 8.2 ppm (^1^ *J*_P‐Pt_ = 3650 Hz), thus indicating a fast displacement of the dmso ligand by the phosphane. In the case of the ^1^H NMR spectrum, a noteworthy feature is the presence of a signal at 8.7 ppm with platinum satellites (^1^ *J*_Pt‐H_ = 27.5 Hz) Both the chemical shift and the magnitude of the coupling constant are compatible with a hydrogen atom interacting with Pt, that is, a hydrogen bond, in a similar fashion to the one described in the complexes depicted in Figure 2.^[^ ^50^, ^51^, ^52^ ^]^ We believe that this 233 K spectrum shows the signals corresponding to an intermediate complex with a Pt···H‐O interaction, as the one represented as **INT2** in Scheme 2. It is noteworthy that in the ^1^H NMR (CD_2_Cl_2_) spectrum of the free phosphane ligand, the signal corresponding to the H hydroxyl group appears at 6.2 ppm. Thus, the deshielding of the previously referred signal (Δ*δ* = +2.5 ppm), together with the presence of ^195^Pt satellites, is another proof of the presence of a Pt···H bond.

When the temperature of the sample is increased to 253 K, signals corresponding to **2** start to show. A progressive augmentation of the NMR recording temperature causes the continuous decrease in the intensity of the signals of **INT2**, and an increase of the intensity of the signals corresponding to the final product **2**, in such a way that at 293 K, 90 minutes after the start of the experiment, only the latter signals are observed (Figure 4a).

**Figure 5:**
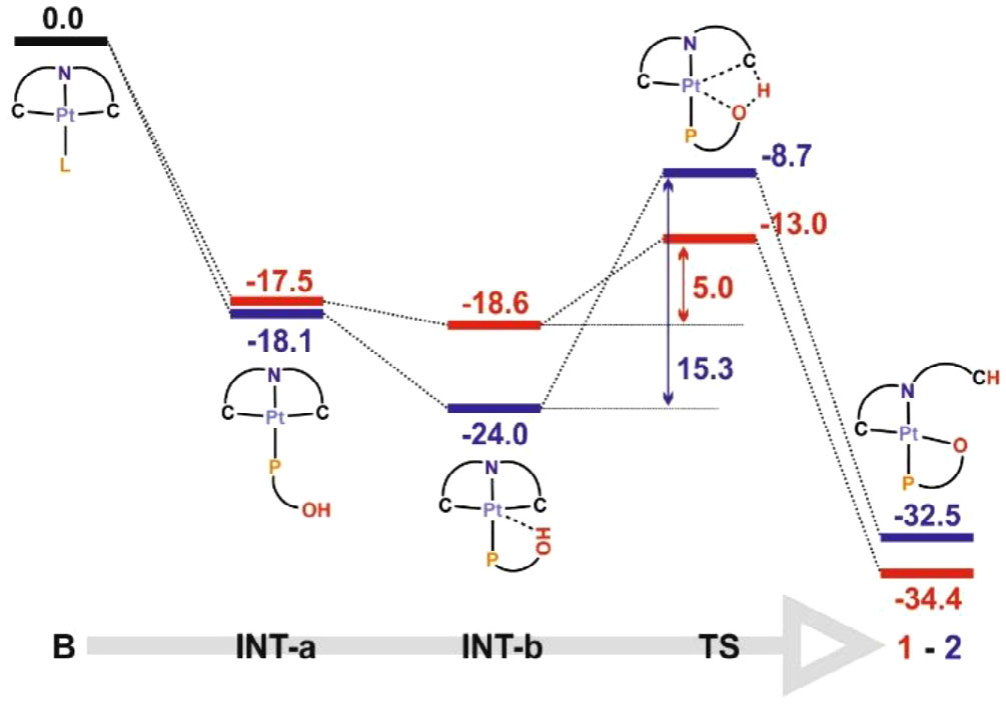
Gibbs Free Energy profile (DFT/BP86‐D3 level, dichloromethane solution) for the reaction of **B** with 2‐(diphenylphosphino)benzoic acid (red) and (2‐hydroxyphenyl)diphenylphosphane (blue). Energies are in kcal/mol.

This different behavior observed in solution for the formation of **1** and **2** prompted us to complete this study by investigating plausible mechanisms for these reactions through DFT calculations (BP86‐D3 level, dichloromethane solution, SMD model; see Computational Details in the Supporting Information). As shown in Figure 5, the global transformations are thermodynamically favorable. The substitution of dmso is easy; therefore, the formation of **INT1a**/**INT2a** and then **INT1b**/**INT2b** is straightforward and takes place immediately, even at low temperature. The optimized geometries of the first computed intermediates display the OH group of the phosphane ligand away from the metallic center. Thereafter, the functionalized phenyl ring can rotate to establish an O‐H···[Pt] interaction (**INT1b** and **INT2b**, respectively).

Interestingly, the formation of the final complexes **1** and **2** from **INT1b** and **INT2b** requires ΔG_act_ of either 5.0 or 15.3 kcal/mol, respectively. This suggests that, whereas spectroscopic observation of the latter intermediate (**INT2b**, in blue) might be possible, the former (**INT1b**, in red) shall evolve to **1** immediately, even at very low temperature. These theoretical results are in excellent agreement with the experimental observations.

Moreover, formation of **2** shall occur via a relatively low energy transition state, **TS2** (426i cm^−1^; ‐OH bond stretching), in which proton transfer from the hydroxyl group to the C_ipso_ (CNC) takes place. The main geometrical features of **INT2b**, the only NMR‐observable intermediate in both reactions, resemble those reported for the 8‐hydroxiquinoline‐based Pt(II) complexes (Figure 2), with the perpendicular disposition of the Pt‐H line to the main square plane and the Pt, and a Pt···H distance of 2.12 Å very similar to those reported for complexes represented in Figure 2.^[^ ^50^, ^51^ ^]^

The novel compounds **1** and **2** are square planar Pt(II) complexes which, as discussed in the introduction, are suitable basic fragments to form heteropolynuclear clusters containing dative Pt→M bonds. Indeed, they resemble [Pt(CNC)(PPh_3_)] (**A**), a complex whose reactivity toward Ag(I) and Au(I) species allowed the synthesis of dinuclear Pt─Ag and Pt─Au, and trinuclear Pt─Ag─Pt and Pt─Au─Pt clusters with striking features.^[^ ^9^, ^41^ ^]^ However, **1** and **2** differ from **A** in that they contain oxygen atoms within their structures, which introduces a potential competition between two basic sites for the coordination of an incoming acidic metal center such as Ag(I) or Au(I). With all this in mind, we have carefully analyzed the reactivity of **1** and **2** toward Ag(I) and Au(I) moieties.

### 2.2 Reactions of 1 and 2 with Ag(I) Moieties

As stated above, Ag(I) is the most common acidic center used in the formation of Pt─M heteropolynuclear clusters with dative bonds. Herein, two kinds of silver precursors have been used: [Ag(OClO_3_)(PPh_3_)] as a source of the [Ag(PPh_3_)]^+^ fragment, and Ag(ClO_4_), a salt that generates “naked” Ag^+^ cations in solution.

**Scheme 3:**
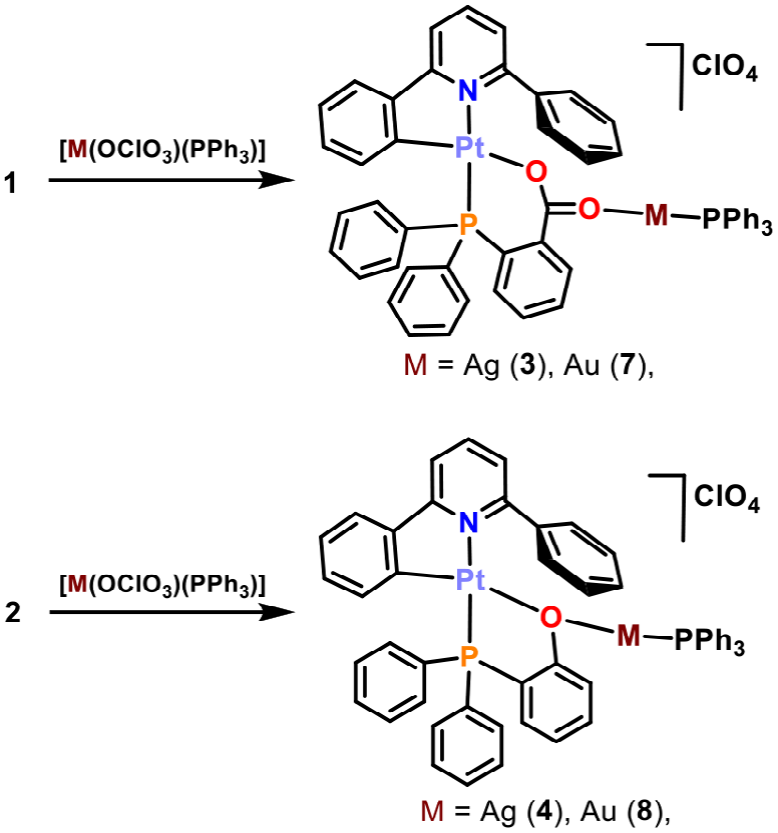
Syntheses of complexes **3**, **4**, **7**, and **8**.

Thus, the reactions of **1** and **2** with [Ag(OClO_3_)(PPh_3_)] (Scheme 3) in 1:1 molar ratio using CH_2_Cl_2_ as solvent produces, after work‐up of the solution, yellow solids which were studied by analytical and spectroscopic means. In the case of the reaction of the complex containing the carboxylic acid phosphane ligand **1**, these studies allow to identify the solid obtained and the sought‐after product [Pt(CNC‐H){PPh_2_(C_6_H_4_‐*o*‐COO)}Ag(PPh_3_)](ClO_4_) (**3**) in 28% yield, whereas the reaction of the starting material **2** produces a mixture of compounds, 4*, that will be described later.

The ^1^H and ^31^P{^1^H} NMR spectra of **3** are in agreement with the proposed formula. In the ^1^H NMR, the presence of the phenyl signals corresponding to the incorporation of the PPh_3_ ligand, along with the signals corresponding to a CNC‐H ligand, are observed. On the other hand, the ^31^P{^1^H} NMR spectrum shows three different signals, a singlet with ^195^Pt satellites corresponding to the PPh_2_(C_6_H_4_‐*o*‐COO) phosphane, and two concentric doublets corresponding to PPh_3_ bonded to the silver center, caused by the coupling with the two active isotopes of Ag (^107^Ag (abundance 51.8%) and ^109^Ag (abundance 48.2%)). The absence of ^195^Pt satellites in these latter signals, a feature sometimes observed in complexes with Pt─Ag bonds, seems to indicate there is no such bond in solution in complex **3**. Moreover, an MS spectrum has confirmed the formation of a Pt‐Ag heterodinuclear complex with the proposed formula (Figure S29, Supporting Information).

**Figure 6:**
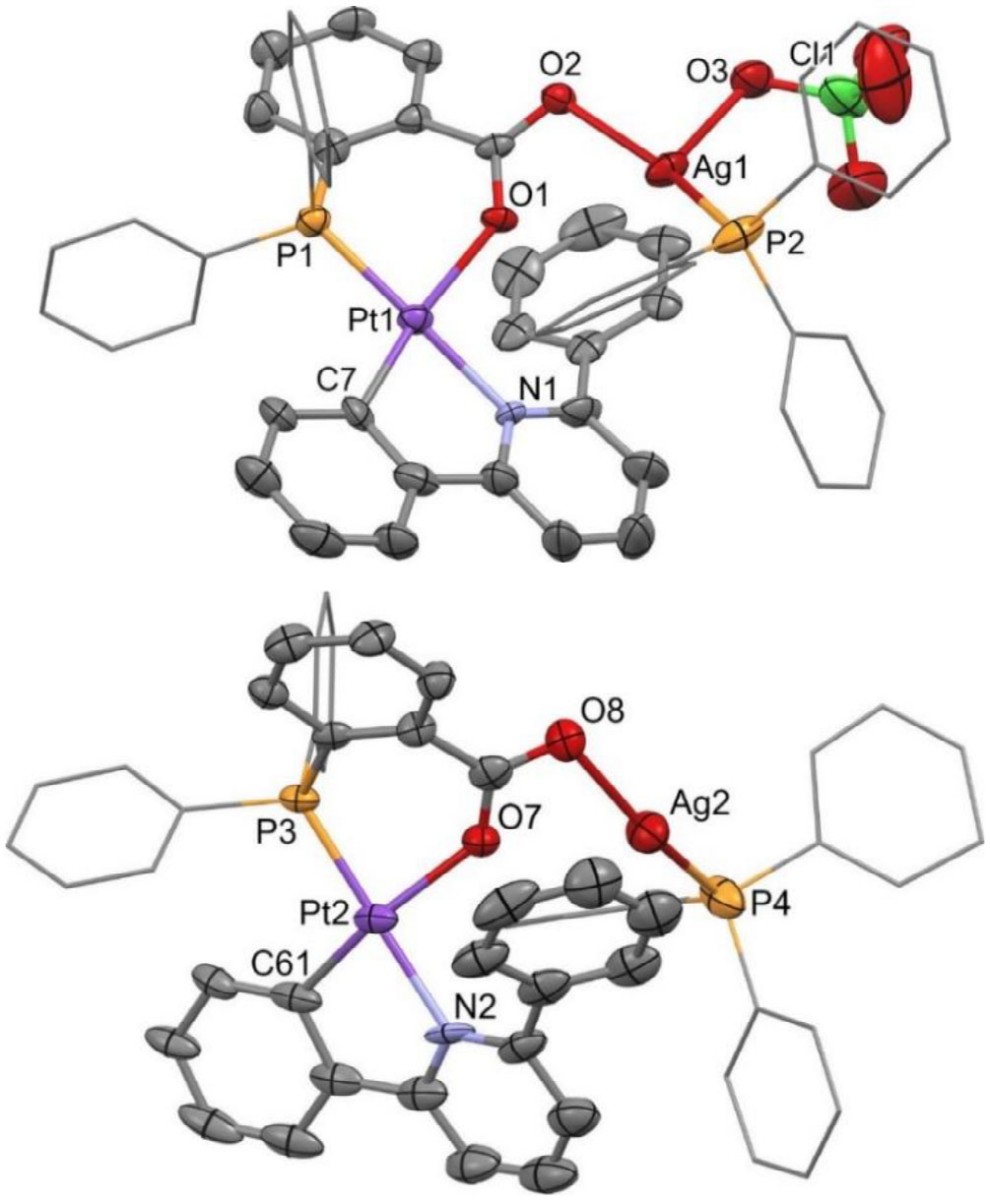
Molecular structure of the two different molecules of **3**, molecule 1 (above) and cationic molecule 2 (below). For the sake of clarity, some phenyl rings are represented in a schematic way.

Suitable single crystals of complex [Pt(CNC‐H){PPh_2_(C_6_H_4_‐*o*‐COO)}Ag(PPh_3_)](ClO_4_) (**3**) could be obtained for an X‐ray diffraction study. Figure 6 shows the obtained structure, whereas its most relevant angles and distances are listed in Table S4 (Supporting Information). The structure of **3** confirms its dinuclear nature. Remarkably, no interactions are established between the Pt and Ag centers, but the latter is coordinated to each “Pt(CNC‐H){PPh_2_(C_6_H_4_‐*o*‐COO)}” unit via the terminal oxygen atom of the carboxylate group. In the asymmetric part of the unit cell there are two molecules of complex **3**, which show striking structural differences. The most relevant one is that in molecule 1 (the one containing the Pt(1) and Ag(1) atoms) the perchlorate cation is coordinated to the Ag(1) center, while in molecule 2 (Pt(2) and Ag(2)) there is no similar interaction. The Ag(1)–O(3) distance is 2.502(7) Å, in the intermediate range described for Ag─OClO_3_ interactions.^[^ ^60^, ^61^ ^]^ On the other hand, the distance from this Ag(1) center to the O(1) oxygen of the carboxylate group, bound to platinum (Ag(1)–O(1) 2.747(5) Å), is longer than the analogue found in molecule 2 (Ag(2)–O(7) 2. 603(7) Å), which could indicate that the silver center in molecule 2 seeks to complete its electron density requirements by approaching the oxygen atom coordinated to the Pt center. In both cases the silver atoms complete their coordination sphere by a conventional bond to the phosphorus atom of a triphenylphosphane ligand. The presence of the interaction with the perchlorate counterion in one of the molecules and its absence in the other is probably due to the packing of the different species in the crystalline environment. Otherwise, as for the silver centers receiving more electron density by forming some kind of *η*‐arene interaction with the phenyl ring of the CNC‐H ligand, the Ag─C distances found are greater than 2.913(9) Å, indicating that, if such an interaction exists, it must be rather weak.

As stated above, the attempts to prepare the analogous complex [Pt(CNC‐H){PPh_2_(C_6_H_4_‐*o*‐O)}Ag(PPh_3_)](ClO_4_) (**4**) yielded a solid that is identified as a mixture of products, 4*. This is evident after inspection of the ^31^P{^1^H} NMR spectra of a solution of the solid (Figure S34 Supporting Information). At room temperature, only a badly resolved spectrum with only a noticeable signal is observed. However, at 173 K, a similar pattern to the one described for **3** (a singlet with ^195^Pt satellites and a pair of concentric doublets corresponding to P bonded to Ag), along with another pair of doublets, the typical pattern for a P atom bonded to silver, is found. The two former signals are compatible with the existence of the sought‐after product [Pt(CNC‐H){PPh_2_(C_6_H_4_‐*o*‐O)}Ag(PPh_3_)](ClO_4_) (**4**), and in fact, the existence of a species with this formulation is confirmed by the MS spectra of the solid 4*.

**Figure 7:**
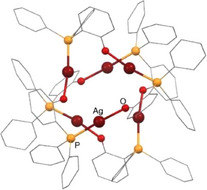
Schematic structure of the complex [Ag{PPh_2_(C_6_H_4_‐*o*‐O)}]_6_.

Unfortunately, no crystals of pure **4** suitable for X‐ray diffraction measurements could be obtained. Instead, only bad‐quality, small, colorless crystals, along with some signs of decomposition are observed in the crystallization tubes. Only an incomplete and low‐quality structure could be determined from those colorless crystals, but the connectivity of the compound could be established. It results in a hexanuclear silver complex of formula [Ag{PPh_2_(C_6_H_4_‐*o*‐O)}]_6_ (Figure 7), in which the phosphane ligand is bridging two silver centers through Ag─P and Ag─O bonds.

Therefore, these results point to the formation of the sought‐after product [Pt(CNC‐H){PPh_2_(C_6_H_4_‐*o*‐O)}Ag(PPh_3_)](ClO_4_) (**4**), which probably bears a similar structure to that found for **3**, with no existence of Pt─Ag bond. This complex would decompose giving the referred hexanuclear silver cluster, likely responsible for the additional signal observed in the ^31^P{^1^H} NMR spectrum of the solid 4*.

**Scheme 4:**
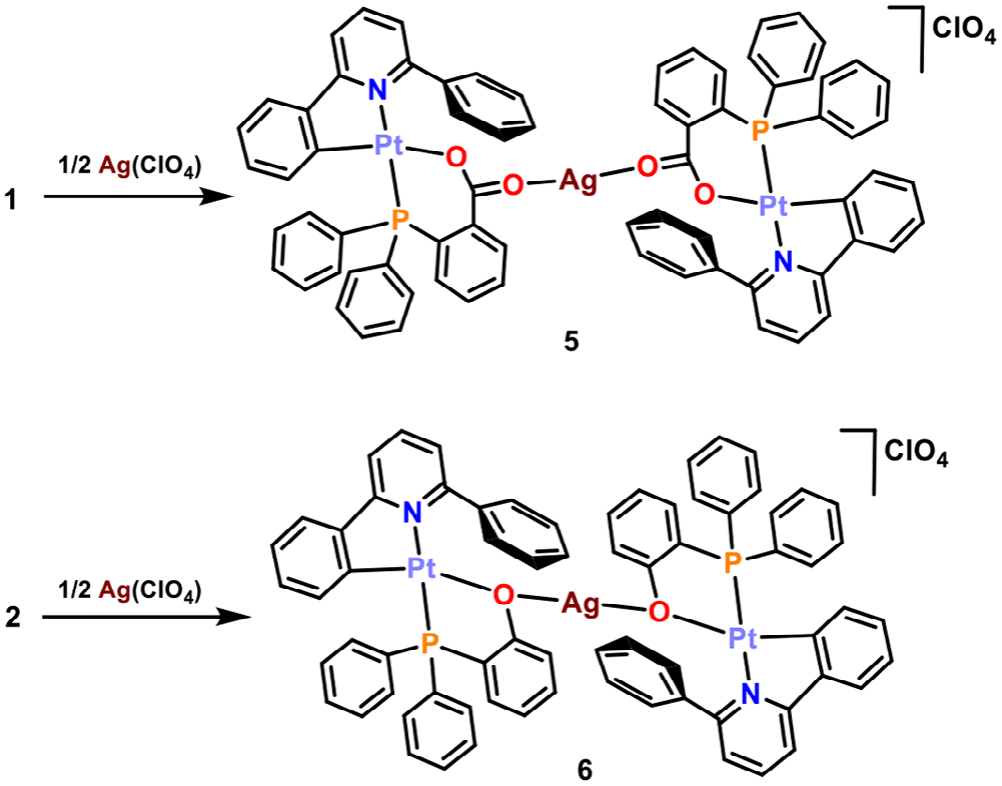
Syntheses of complexes **5** and **6**.

On the other hand, the reactions of the starting materials **1** and **2** with the silver(I) salt Ag(ClO_4_) in acetone in 2:1 molar ratio cause an intensification of the yellow color of the solutions. After 60 minutes of stirring in the absence of light, evaporation of the solvent renders orange‐yellow solids in both cases. The analytical and spectroscopic data allow the assignation of the formulas [{Pt(CNC‐H){PPh_2_(C_6_H_4_‐*o*‐COO)}}_2_Ag](ClO_4_) (**5**) and [{Pt(CNC‐H){PPh_2_(C_6_H_4_‐*o*‐O)}}_2_Ag](ClO_4_) (**6**), respectively, for these solids (36% and 73% yield, respectively; Scheme 4). Their IR spectra confirm the presence of the ClO_4_^−^ anion, whereas their MS spectra show peaks consistent with each of the proposed formulas for the corresponding cations (Figures S44, S53, Supporting Information).

The ^1^H and ^31^P{^1^H} NMR spectra of compounds **5** and **6** show analogous patterns to the ones described above for the starting materials **1** and **2**, but with slight differences in the chemical displacements of certain signals. However, no further information about the structural role played by the silver centers can be extracted from them.

**Figure 8:**
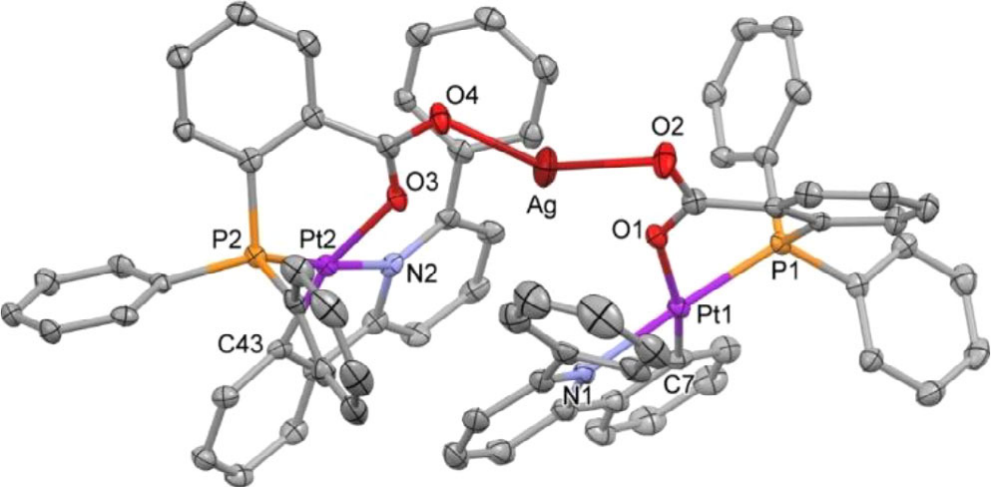
Molecular structure of the cation of complex [{Pt(CNC‐H){PPh_2_(C_6_H_4_‐*o*‐COO)}}_2_Ag](ClO_4_) (**5**).

We obtained single crystals suitable for their study by X‐ray diffraction, and thus the structures of both **5** and **6** were determined. Figure 8 shows a view of the structure of the cation of **5**, while Table S5 (Supporting Information) lists relevant bond distances and angles. **5** is a trinuclear complex formed by two “Pt(CNC‐H){PPh_2_(C_6_H_4_‐*o*‐COO)}” units linked by a Ag^+^ acidic center acting as a bridge between them. As observed for complex **3**, in **5** the silver center bonds the terminal oxygen atom of the carboxylate group. Thus, two Ag─O bonds of identical length within the experimental error are formed (2.203(3) and 2.205(3) Å). The distances between the silver center and the oxygen atoms of the carboxylate groups coordinated to platinum are considerably longer (2.760(3) and 2.893(2) Å), effectively ruling out the presence of a direct Pt‐Ag bond.

The O(4)–Ag–O(2) angle, 142.86(11)°, deviates from a linear arrangement typical for an Ag(I) environment. Besides, there is some closeness between one of the carbon atoms of each of the phenyl rings of the CNC‐H ligands and the silver atom, with interatomic distances of 2.849(6) (Ag─C(17)) and 2.891(5) Å (Ag─C(52)). These are long for a conventional *η^1^* interaction,^[^ ^62^ ^]^ but could indicate a weak contact between the aromatic ring and the silver center.

**Figure 9:**
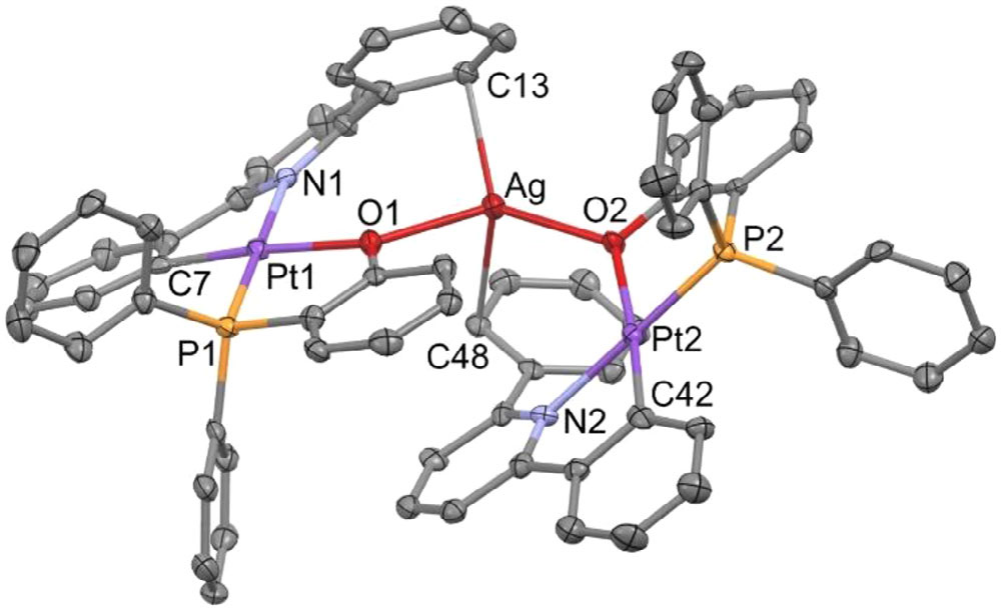
Molecular structure of the cation of complex [{Pt(CNC‐H){PPh_2_(C_6_H_4_‐*o*‐O)}}_2_Ag](ClO_4_) (**6**).

Figure 9 shows a view of the structure of the cation of **6**, and Table S6 (Supporting Information) lists relevant bond distances and angles. **6** is a trinuclear complex in which two platinum units “Pt(CNC‐H){PPh_2_(C_6_H_4_‐*o*‐O)}” are connected by an Ag^+^ center. This center bonds to the oxygen atom of the chelated phosphane of each of the Pt units (Ag─O(1) 2.337(3), Ag─O(2) 2.258(3) Å) with no proof of any Pt‐Ag interaction. The silver center establishes two *η^1^* contacts with two atoms of each of the phenyl rings of the CNC‐H ligands, with distances Ag─C(13) 2.424(5) and Ag─C(48) 2.531(5) Å. This is certainly favored by the closer approximation of Ag to the core of the platinum units and to the phenyl ring of the CNC‐H ligand. Thus, the resulting environment for the silver atom is somewhat distorted tetrahedral. The establishment of *η^1^*‐Ag─C interactions is noteworthy and probably favored by the fact that the oxygen atom bonded to the silver center is the one that is also bonded to the platinum center. Consequently, a closer approximation of Ag to the core of the platinum units and to the phenyl ring of the CNC‐H ligand is possible.

### 2.3 Reactions of 1 and 2 with an Au(I) Moiety

As with silver(I), gold(I) moieties can behave as acidic centers able to interact with basic fragments, such as Pt(II) in square planar complexes. However, there is not a ready‐to‐use gold precursor that behaves as the fragment “Au(PPh_3_)^+^” in solution. For this reason, it has to be generated in situ by reacting [AuCl(PPh_3_)] with Ag(ClO_4_). Thus, to solutions of [Au(Cl)(PPh_3_)] in THF under argon at ‐65 °C, equimolar amounts of AgClO_4_ were added, causing the formation of a white precipitate (AgCl). Over these suspensions, [Pt(CNC‐H){PPh_2_(C_6_H_4_‐*o*‐COO)}] (**1**) or [Pt(CNC‐H){PPh_2_(C_6_H_4_‐*o*‐O)}] (**2**) were added in molar ratios of 1:1. The mixtures were stirred for 1 hour, and the temperature was allowed to rise to ‐15 °C, the AgCl precipitate was filtered off and, after work up, yellow solids were obtained. The analytical and spectroscopic data retrieved for them allowed to assign the formulas [Pt(CNC‐H){PPh_2_(C_6_H_4_‐*o*‐COO)}Au(PPh_3_)](ClO_4_) (**7**) and [Pt(CNC‐H){PPh_2_(C_6_H_4_‐*o*‐O)}Au(PPh_3_)](ClO_4_) (**8**), respectively (68% and 73% yield, respectively; Scheme 3).

The stability of **7** and **8** in solution seems to be limited, especially for the latter. Indeed, solutions of these complexes, even if maintained refrigerated, show signs of slow but continuous decomposition, by the appearance of a purple solid. In the ^1^H NMR spectra of **7** and **8**, shifts in the chemical displacements of the signals of the CNC‐H ligand with respect to those observed for the starting materials are observed, together with the presence of new aromatic signals corresponding to a PPh_3_ ligand. On the other hand, the ^31^P{^1^H} NMR spectra of **7** and **8** show two singlets each, corresponding to the phosphorus atoms of the two phosphane ligands. One of these singlets shows ^195^Pt satellites and is assigned to the chelate phosphane bonded to Pt. The absence of ^195^Pt satellites in the other signal, assigned to a phosphane ligand attached to Au, suggests there is no direct Pt─Au bond either in **7** or in **8**. Finally, their MS spectra show in each case a set of peaks with a pattern consistent with their proposed formulas.

Unfortunately, we failed in the obtaining of crystals of either **7** or **8**. Thus, on the basis of the spectroscopic data available, we tentatively propose structures similar to the ones found for **3** and **4**, with no Pt─Au bonds and Au─O bonds instead.

### 2.4 Reactions of 5 and 6 with H^+^

As mentioned, the attempts to obtain crystals of the gold complexes **7** and **8** invariably failed, and we typically observed the formation of purple solids, indicative of decomposition. However, in some cases, the presence of a few yellow crystals was also observed. These crystals were good enough to perform X‐ray structure determinations.

**Figure 10:**
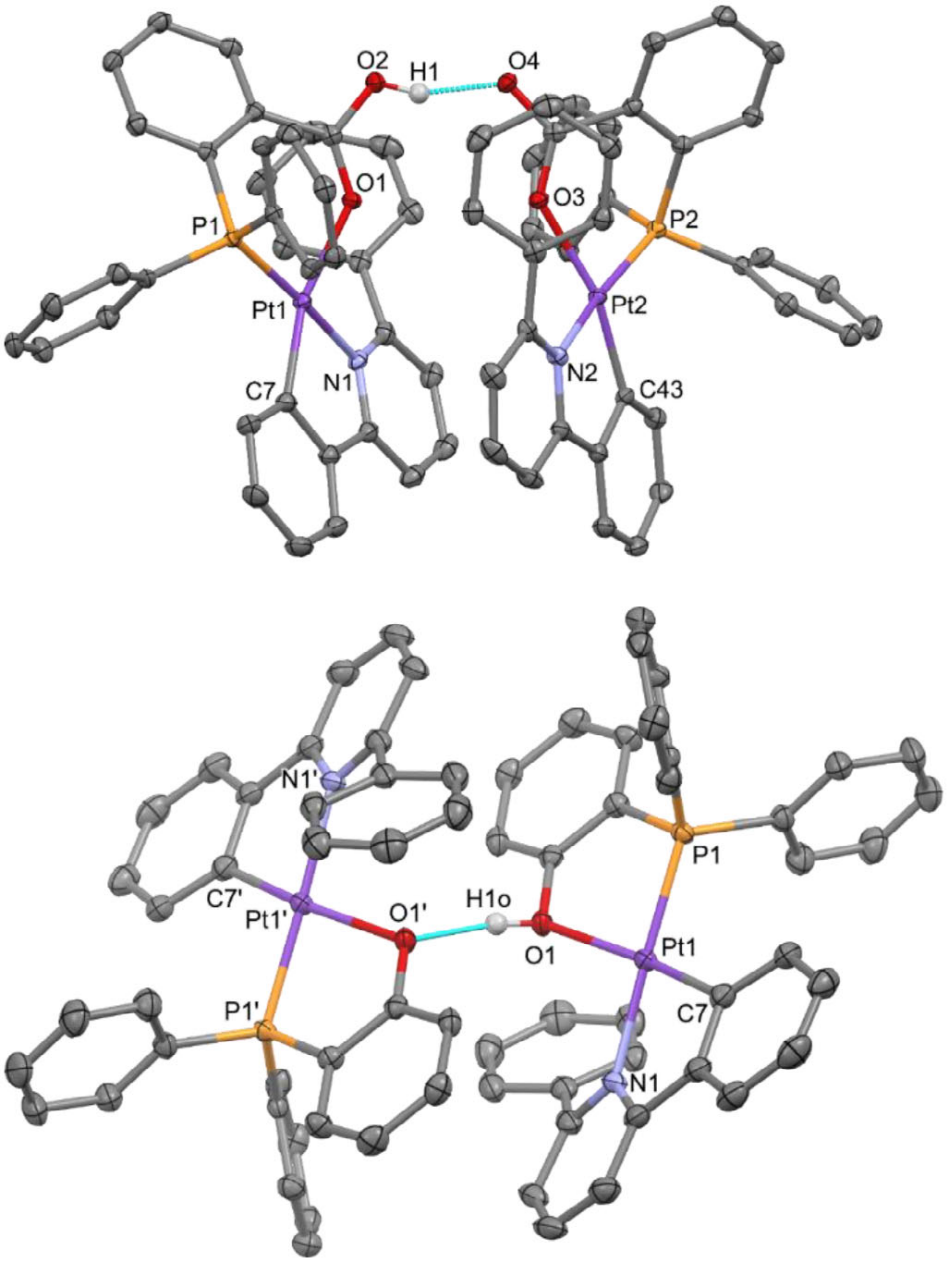
Molecular structure of the cations of the complexes [{Pt(CNC‐H){PPh_2_(C_6_H_4_‐*o*‐COOH)}}{Pt(CNC‐H){PPh_2_(C_6_H_4_‐*o*‐COO)}}](ClO_4_) (**9**, above) and [{Pt(CNC‐H){PPh_2_(C_6_H_4_‐*o*‐OH)}}{Pt(CNC‐H){PPh_2_(C_6_H_4_‐*o*‐O)}}](ClO_4_) (**10**, below).

Surprisingly, these structures showed no gold but revealed the formation of homodinuclear platinum complexes of formulas [{Pt(CNC‐H){PPh_2_(C_6_H_4_‐*o*‐COOH)}}{Pt(CNC‐H){PPh_2_(C_6_H_4_‐*o*‐COO)}}](ClO_4_) (**9**) and [{Pt(CNC‐H){PPh_2_(C_6_H_4_‐*o*‐OH)}}{Pt(CNC‐H){PPh_2_(C_6_H_4_‐*o*‐O)}}](ClO_4_) (**10**) (Figure 10; Tables S7, S8 at the Supporting Information list relevant bond distances and angles). The presence of the perchlorate anions indicates the cationic character of the metallic entities. However, in the absence of other heavy atoms and in view of the short O···O distances (2.461(2) Å for **9**, 2.460(5) Å (average) for **10**), the presence of a bridging proton can be inferred. Indeed, close inspection of the X‐ray electron density maps allowed to locate and further refine good‐quality geometrical parameters for a O─H···O hydrogen bond system (Table S9, Supporting Information).

Interestingly, a close inspection of the structures of **9** and **10**, and those of their analogues **5** and **6** (Figures 8, 9) discloses a very similar disposition of all of their constitutive elements. Thus, in both cases, the acidic centers (Ag^+^ or H^+^) are bridging two Pt units through oxygen atoms. In the silver case the O─Ag─O systems are symmetrical, while in **9**, the O─H···O system seems to be unsymmetrical, which is characteristic for hydrogen bonding. In addition, the large difference in size between Ag^+^ and H^+^ causes a marked variation in the separation of the two Pt complexes (6.548(1) Å in **5** vs. 5.694(1) Å in **9**; 6.898(1) Å in **6** vs. 5.957(1) Å in **10**).

**Scheme 5:**
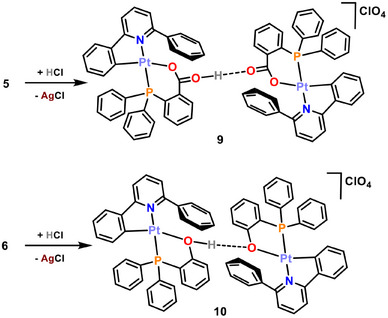
Syntheses of complexes **9** and **10**.

The unexpected formation of crystals of **9** and **10** has to be related to the decomposition of the gold complexes **7** and **8** in the crystallization tubes. Keeping this in mind, we have tried the deliberate preparation of these complexes by reacting CH_2_Cl_2_ solutions of complexes **5** and **6** with solutions of HCl in MeOH/H_2_O in molar ratios 1:1 (Scheme 5). After a few minutes, a white precipitate corresponding to AgCl is observed in both cases, which is filtered off. Workup of the resulting solutions led to the isolation of pale yellow solids, identified as **9** and **10**, in 89% and 58% yield, respectively.

The IR of these solids confirms the presence of the perchlorate cation, whereas the ^1^H and ^31^P{^1^H} NMR spectra of compounds **9** and **10** show similar patterns as the ones described above for the starting materials **1** and **2**, or the silver ones **5** and **6**, with slight differences in the observed chemical displacements. No signals attributable to the H atom involved in the hydrogen bonding interaction can be identified in the ^1^H NMR spectra at room temperature, possibly due to either the fact that this signal is embedded in the multiplets corresponding to the aromatic protons of the ligands, or to a rapid dynamic proton transfer between the two bridged oxygen atoms. Lowering the temperature down to 213 K did not allow to locate a signal for this nucleus. Finally, X‐ray diffraction studies on crystals grown from these solids confirmed their identity as complexes **9** and **10**.

### 2.5 Complexes 3, 4, 7, and 8 from the Perspective of Lewis Acid‐Lewis Base Interactions

The formation of complexes **3**, **4**, **7**, and **8** as a result of a Lewis pair assembly of two metal fragments raises some interesting questions, particularly in light of the structures of other heteropolymetallic complexes reported in the literature. In the current study, the presence of oxygen atoms within the structures of the platinum precursors unveils a competition between basic sites for the coordination of an incoming acidic metal center, thus generating a substantial effect on the thermodynamically favored products. This is evident from the inspection of the seven XRD structures described herein. Unfortunately, we have structural data for only one of the above‐referred four derivatives (**3**), but DFT calculations can provide some further insights on this set of complexes.

Moreover, in this broad context we (and others) have postulated that the interaction of a Lewis Acid (LA) metal fragment with the electron density of a Pt‐C or Pd‐C bond of a complex acting as an LB can trigger a transmetallation process.^[^ ^32^, ^42^, ^63^, ^64^, ^65^ ^]^ Consequently, the introduction of additional basic sites through complex design might have an impact that can be potentially advantageous for the ulterior design of catalytic systems, in the field of cooperative catalysis.

Insisting on this idea, we remark that the presence of several basic spots in a LB Pt complex, as is the case for **1** and **2**, can modulate the importance of acid‐base interactions. Thus, with all this in mind, we have carefully analyzed the Potential Energy Surface (PES) at the BP86‐D3 level of theory, in dichloromethane solution (SMD model), for the possible adducts resulting from the interaction between the starting materials **1** and **2**, and the [Ag(PPh_3_)]^+^ and [Au(PPh_3_)]^+^ LAs, trying to establish an energy profile relating the different possible LB‐LA isomers. Comparison of the computed minimum for the **1**‐[Ag(PPh_3_)]^+^ adduct with the XRD structure available (**3**, see above) is pertinent, but we note that the different conditions of the experimental measurement (solid‐state crystalline material in a 3D lattice) and the modelled system (discrete chemical entity, in dichlorometane solution) shall give rise to certain differences.

**Figure 11:**
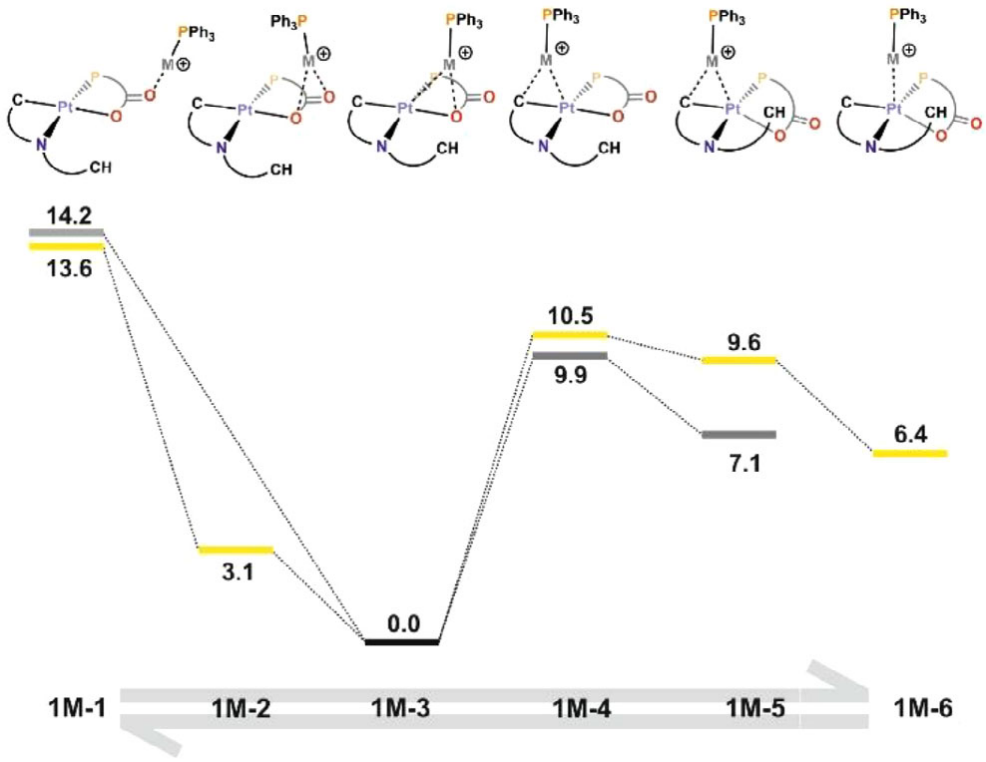
Gibbs Free Energy profile (DFT/BP86‐D3 level, dichloromethane solution) for the LB‐LA interaction of **1** with the [Ag(PPh_3_)]^+^ (gray) and [Au(PPh_3_)]^+^ (yellow) fragments. Energies are in kcal/mol.

**Figure 12:**
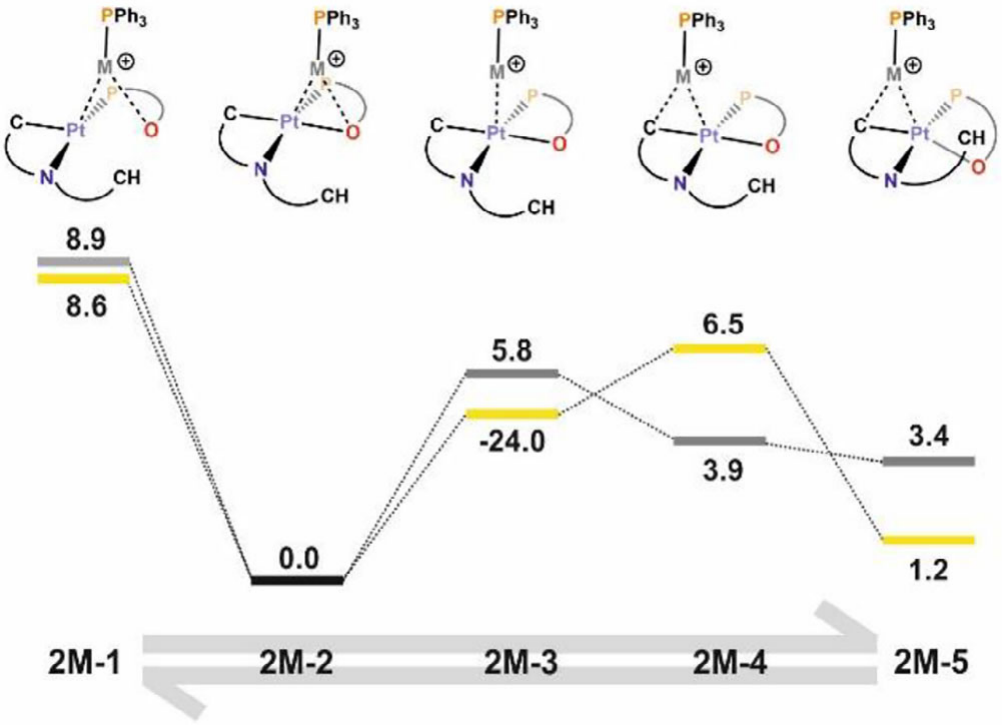
Gibbs Free Energy profile (DFT/BP86‐D3 level, dichloromethane solution) for the LB‐LA interaction of **2** with the [Ag(PPh_3_)]^+^ (gray) and [Au(PPh_3_)]^+^ (yellow) fragments. Energies are in kcal/mol.

Thus, regarding precursor **1**, the two profiles obtained (Figure 11) show qualitative similarity in both cases, with the adduct resulting from the interaction of the LA fragment with the electron density of the Pt‐O axis (**1M‐3**) being the most favored one. The free energy differences with regard to the adducts resulting from the interaction with the Pt‐C axis (**1M‐5**) are 7.1 (M = Ag) and 9.6 (M = Au) kcal/mol. Focusing on precursor **2**, the two profiles obtained (Figure 12) are also qualitatively similar to one another, again with the adduct resulting from the interaction with the Pt‐O axis (**2M‐2**) as the most favored one. Interestingly, the adducts resulting from the interaction with the Pt‐C axis (**2M‐5**) are markedly less disfavored than in the previous case, by 3.4 (M = Ag) and 1.2 (M = Au) kcal/mol.

These profiles illustrate how the use of P,O‐bidentate ligands with their potentially O donor atoms favors the formation of the LB‐LA adduct through the Pt‐O axis over the Pt‐C axis. In general, this is in agreement with the observed crystal structures **3**, **5,** and **6** reported here. Since the interaction with the Pt‐C axis is a necessary intermediate for a subsequent transmetallation, this emphasizes the importance of the selection of auxiliary ligands in the design of LB fragments to modulate LB‐LA interactions in the intermediates of transmetallation processes.

## 3 Conclusion

The basic properties of the platinum (II) center in square planar complexes have been analyzed through the reaction of the solvated starting material **B** with phosphano ligands containing an acidic hydrogen atom in their structure, such as 2‐(diphenylphosphino)benzoic acid (PPh_2_(C_6_H_4_‐*o*‐COOH) and (2‐hydroxyphenyl)diphenylphosphane (PPh_2_(C_6_H_4_‐*o*‐OH) (Scheme 2). Only the final proton transfer products **1** and **2** can be isolated from these reactions. These complexes can be regarded as the evolution of an intermediate showing a Pt→H interaction as represented in Scheme 1 (type a). However, in the case of **2**, the hydrogen‐bonding intermediate can be detected by low‐temperature NMR. This rapid evolution is likely related to the planar nature of the CNC ligand present in **B** and the accessibility to an intermediate situation of type b in Scheme 1. The energy profile calculated (Figure 5) supports this point. Such type of behavior is in agreement with the structures of type B found in Pt→M (M = Ag, Au) complexes also containing the CNC ligand (Figure 1), in which the planarity of the Pt fragment favors the evolution of the Lewis acid‐base pairs interaction until this intermediate point.

On the other hand, **1** and **2** are still suitable precursors for the preparation of heterobimetallic clusters. However, the presence of oxygen atoms in competition with the metal center as Lewis basic points leads to the formation of Pt─O─Ag─O─Pt or Pt─O─M(PPh_3_) complexes with no metal‐metal bonds, thus indicating that Lewis donor‐acceptor metal‐metal interactions are weak and easily replaceable. Surprisingly, dimers Pt─O─H···O─Pt (**9** and **10**) have been identified as decomposition products of the Pt/Au complexes. Synthetic routes for these complexes have been developed for them, and they have been fully characterized.

The latter are noteworthy as they support the idea of a comparable behavior of electrophilic fragments as a proton (H^+^), a naked metal (Ag^+^, Au^+^), and a metal‐ligand fragment (Ag(PPh_3_)^+^, Au(PPh_3_)^+^). These observations are consistent with the well‐known isolobality concept, that relates some of these chemical fragments. It is also worth noting that the addition of another acidic proton to compounds **1** or **2** does not result in a second protonation of the CNC‐H ligand, but rather occurs at the oxygen atom. The presence of this nucleophillic spot likely prevents a mechanism as proposed in Scheme 1, as no initial Pt···H interaction takes place.

## Conflict of Interest

The authors declare no conflict of interest.

## Supporting information



Supporting Information

Supporting Information

## Data Availability

The data that support the findings of this study are available in the supplementary material of this article.
