# Supplementary material for: Reactivity of Square Planar Pt(II) Complexes Toward Acidic Moieties as H+, M+, and [ML]+ (M = Ag, Au)
Source: Chemistry. 2025 Sep 17;31(59):e02370. doi: 10.1002/chem.202502370 (PMC12548521; doi:10.1002/chem.202502370)
Supplement: Supplementary file 1 — Supporting Information [file CHEM-31-e02370-s001.pdf]

Supporting Information

for

Reactivity of Square Planar Pt(II) Complexes Towards Acidic Moieties as H^+, M^+, and $[ML]^+$ (M = Ag, Au)

Laura Martínez Romera,[a] David Campillo,[a] Daniel Escudero,[b] Miguel Baya,[a] Antonio Martín*,[a]

[a] *Instituto de Síntesis Química y Catálisis Homogénea (ISQCH), CSIC-Universidad de Zaragoza, C/Pedro Cerbuna 12, 50009 Zaragoza, Spain*

[b] *Department of Chemistry, Katholieke Universiteit Leuven, Celestijnenlaan 200 f – Box 2404, 3001 Leuven, Belgium*

- 1. Experimental details S2
- 2. IR, NMR and MS spectra of complexes S11
- 3. Crystal data and structural refinement S62
- 4. Computational details S72
- 5. References S73

1. Experimental details

General procedures and materials. Elemental analyses were carried out with a Perkin-Elmer 2400 CHNS analyzer. IR spectra were recorded on a Perkin-Elmer Spectrum 100 FT-IR spectrometer (ATR in the range 250-4000 cm^{-1}). Mass spectrometry was performed with the Microflex matrix-assisted laser desorption ionization-time-of-flight (MALDI-TOF) Bruker or an Autoflex III MALDI-TOF Bruker instruments. ^{1}H, $^{13}C\{1H\}$, $^{31}P\{1H\}$ were recorded on Bruker ARX-300, AV-400 and AV-500 spectrometers using the standard references: $SiMe_4$ and 85% H_3PO_4 for references for ^{1}H and ^{13}C, ^{31}P respectively. The signal attributions and coupling constant assessment was made on the basis of a multinuclear NMR analysis of each compound including, besides 1D spectra, ^{1}H-^{1}H COSY, ^{1}H-^{13}C HMQC, ^{1}H-^{13}C HMBC and $^{13}C\{^{1}H\}$ APT. [Pt(CNC)(dmso)] was prepared following the procedure described in the bibliography.[1]

Safety Note: Perchlorate salts of metal complexes with organic ligands are potentially explosive. Only small amounts of material should be prepared, and these should be handled with great caution.

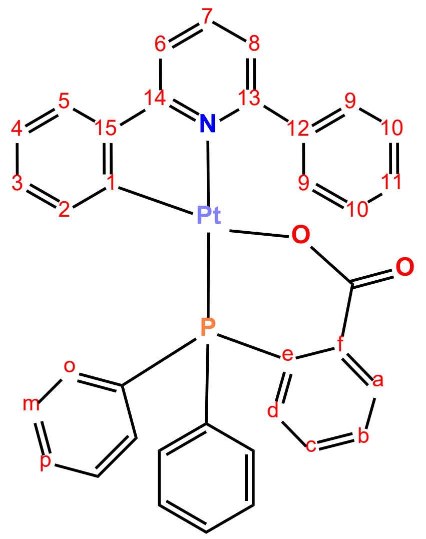

Chart 1. ^{1}H and ^{13}C NMR numbering scheme.

Preparation of the complex [Pt(CNC-H){PPh$_2$(C$_6$H$_4$-*o*-COO)}] (1). To a solution of [Pt(CNC)(dmso)] (0.050 g, 0.099 mmol) in 20 mL of CH_2Cl_2, 2-(diphenylphosphino)benzoic acid (0.031 g, 0.099 mmol) was added. After 25 minutess of reaction, the resulting bright yellow solution was evaporated to dryness. To the resulting yellow solid 10 mL of CH_2Cl_2 were added and the

solution was again evaporated to dryness. After three times of washing and drying the solid with CH_2Cl_2 it was extracted with 10 mL of diethylether, filtered and air-dried (0.059 g, 81%). Anal. Found: C, 58.93; H, 3.62; N, 1.54. Calcd. for $C_{36}H_{26}NO_2PPt$: C, 59.18; H, 3.59; N, 1.92. IR (ATR, cm^{-1}): 1623 (s, υ(C=O)), 1602 (w, υ(N-C)), 1580 (m, υ(N-C)), 1559 (w, υ(N-C)), 541 (s, υ(P-C)), 514 (s, υ(P-C)). ^1H NMR (400.132 MHz, CD_2Cl_2, 293K, see Chart 1 for the H numbering scheme): δ = 7.98 (3H, m, H7 y H9), 7.89 (1H, d, $^3J_{H6-H7}$ = 7.9 Hz, H6), 7.75 (1H, m, Ha), 7.64 (1H, d, $^3J_{H5-H4}$ = 7.6 Hz, H5), 7.56 (7H, m, *o*-PPh_2, *p*-PPh_2 y H8), 7.44 (5H, m, *m*-PPh_2 y Hb), 7.28 (2H, m, H11 y Hc), 7.22 (2H, t, $^3J_{H10-H9}$ = $^3J_{H10-H11}$ = 7.2 Hz, H10), 7.02 (1H, t, $^3J_{H4-H5}$ = $^3J_{H4-H3}$ = 7.6 Hz, H4), 6.79 (1H, m, Hd), 6.61 (1H, t, $^3J_{H3-H2}$ = $^3J_{H3-H4}$ = 7.6 Hz, H3), 6.33 (1H, dd, $^3J_{H2-H3}$ = 7.6 Hz, $^4J_{H2-P}$ = 3.0 Hz, $^3J_{H2-Pt}$ = 55.4 Hz, H2) ppm. $^{31}P\{^1H\}$ NMR (161.923 MHz, CD_2Cl_2, 293K): δ = 11.1 (s, $^1J_{P-Pt}$ = 4388 Hz) ppm. $^{13}C\{^1H\}$ NMR plus HSQC and HMBC (100.624 MHz, CD_2Cl_2, 293K, see Chart 1 for the C numbering scheme): δ = 166.6 (s, COO), 164.4 (s, C14), 160.8 (s, C13), 146.8 (s, C15), 143.3 (s, Cf), 139.8 (s, C7), 139.3 (s, C12), 136.7 (d, $^3J_{C-P}$ = 4.9 Hz, C2), 135.5 (d, $^2J_{C-P}$ = 4.2 Hz, C1), 134.8 (d, $^2J_{C-P}$ = 13.0 Hz, *o*-PPh_2), 133.0 (d, $^3J_{C-P}$ = 9.8 Hz, Ca), 132.5 (d, $^2J_{C-P}$ = 5.4 Hz, Cd), 132.1 (d, $^4J_{C-P}$ = 2.6 Hz, *p*-PPh_2), 130.9 (d, $^4J_{C-P}$ = 2.6 Hz, Cb), 129.6 (s, C11), 129.3 (d, $^3J_{C-P}$ = 11.2 Hz, *m*-PPh_2), 129.2 (s, C9), 129.1 (s, C3), 128.9 (d, $^3J_{C-P}$ = 8.9 Hz, Cc), 128.3 (s, C10), 128.2 (d, $^1J_{C-P}$ = 61.6 Hz, *q*-PPh_2), 126.6 (d, $^1J_{C-P}$ = 60.9 Hz, Ce), 124.8 (s, C5), 124.5 (d. $^4J_{C-P}$ = 4.3 Hz, C8), 123.8 (s, C4), 116.9 (d, $^4J_{C-P}$ = 2.0 Hz, C6) ppm. MS MALDI+ DCTB: m/z = 731.2 [[Pt(CNC-H)(C_6H_4-*o*-COO)]+H]$^+$.

Preparation of the complex [Pt(CNC-H){PPh_2(C_6H_4-*o*-O)} (2). To a solution of [Pt(CNC)(dmso)] (0.070 g, 0.139 mmol) in 20 mL of CH_2Cl_2, (2-hydroxyphenyl)diphenylphosphane (0.039 g, 0.142 mmol) under argon was added. After 25 minutes of reaction, the resulting yellow solution was concentrated to *ca.* 2 mL and 15 mL of *n*-hexane were added. The yellow solid formed was then filtered and air-dried (0.052 g, 53%). Anal. Found: C, 60.20; H, 3.73; N, 1.64. Calcd. for $C_{35}H_{26}NOPPt$: C, 59.83; H, 3.73; N, 1.99. IR (ATR, cm^{-1}): 1596 (w, υ(N-C)), 1578 (w, υ(N-C)), 1564 (s, υ(N-C)), 1551 (w, υ(N-C)), 532 (s, υ(P-C)), 509 (s, υ(P-C)), 489 (s, υ(P-C)). ^1H NMR (400.132 MHz, CD_2Cl_2, 293K, see Chart 1 for the H numbering scheme): δ = 7.96 (1H, t, $^3J_{H7-H6}$ = $^3J_{H7-H8}$

= 7.8 Hz, H7), 7.90 (2H, dd, $^3J_{H9\text{-}H10}$ = 7.8 Hz, $^4J_{H9\text{-}H11}$ = 1.5 Hz, H9), 7.86 (1H, d, $^3J_{H6\text{-}H7}$ = 7.8 Hz, H6), 7.80 (4H, m, *o*-PPh_2), 7.59 (1H, dd, $^3J_{H5\text{-}H4}$ = 7.6 Hz, $^4J_{H5\text{-}H3}$ = 1.0 Hz, H5), 7.53 (1H, d, $^3J_{H8\text{-}H7}$ = 7.6 Hz, H8), 7.44 (8H, m, H10, H11, *m*-PPh_2 y *p*-PPh_2), 7.15 (1H, dd, $^3J_{H2\text{-}H3}$ = 7.6 Hz, $^4J_{H2\text{-}H4}$ = 1.2 Hz, $^3J_{H2\text{-}Pt}$ = 54.0 Hz, H2), 7.02 (1H, td, $^3J_{H4\text{-}H5}$ = $^3J_{H4\text{-}H3}$ = 7.6 Hz, $^4J_{H4\text{-}H2}$ = 1.2 Hz, H4), 6.90 (2H, m, Ha y Hc), 6.78 (1H, td, $^3J_{H3\text{-}H2}$ = $^3J_{H3\text{-}H4}$ = 7.6 Hz, $^4J_{H3\text{-}H5}$ = 1.0 Hz, H3), 6.26 (1H, t, $^3J_{Ha\text{-}Hb}$ = $^4J_{Ha\text{-}P}$ = 7.6 Hz, Hb), 5.77 (1H, m, Hd) ppm. $^{31}P\{^1H\}$ NMR (161.923 MHz, CD_2Cl_2, 293K): δ = 20.2 (s, $^1J_{P\text{-}Pt}$ = 4435 Hz) ppm. $^{13}C\{^1H\}$ NMR plus HSQC and HMBC (100.624 MHz, CD_2Cl_2, 293K, see Chart 1 for the C numbering scheme): δ = 177.0 (s, Cf), 165.4 (s, C14), 162.1 (s, C13), 146.9 (s, C15), 146.9 (s, C1), 139.8 (s, C12), 139.7 (s, C7), 138.9 (d, $^3J_{C\text{-}P}$ = 5.7 Hz, C2), 134.1 (d, $^2J_{C\text{-}P}$ = 11.7 Hz, *o*-PPh_2), 133.4 (d, $^3J_{C\text{-}P}$ = 1.5 Hz, Ca o Cc), 131.9 (s, Ca o Cc), 131.3 (d, $^4J_{C\text{-}P}$ = 2.6 Hz, *p*-PPh_2), 131.1 (d, $^1J_{C\text{-}P}$ = 58.0 Hz, *q*-PPh_2), 129.9 (s, C3), 129.8 (s, C9), 129.4 (s, C11), 129.0 (d, $^3J_{C\text{-}P}$ = 11.1 Hz, *m*-PPh_2), 127.7 (s, C10), 124.7 (s, C5), 124.4 (s, C8), 123.3 (s, C4), 119.5 (d, $^2J_{C\text{-}P}$ = 9.2 Hz, Cd), 117.1 (s, C6), 117.0 (d, $^1J_{C\text{-}P}$ = 65.0 Hz, Ce), 114.3 (d, $^4J_{C\text{-}P}$ = 9.3 Hz, Cb) ppm. MS ESI+: m/z = 702.5 [[Pt(CNC-H){$PPh_2(C_6H_4$-*o*-O)}]+H]$^+$.

Preparation of the complex [{Pt(CNC-H){$PPh_2(C_6H_4$-*o*-COO)}}Ag(PPh_3)](ClO_4) (3). To a solution of [Pt(CNC-H){$PPh_2(C_6H_4$-*o*-COO)}] (**1**) (0.070 g, 0.096 mmol) in 20 mL of CH_2Cl_2 at room temperature [Ag($OClO_3$)(PPh_3)] (0.045 g, 0.096 mmol) was added. After 4 hours of stirring in the dark, the resulting yellow solution was evaporated to dryness. The resulting yellow solid was extracted with 10 mL of *n*-hexane, filtered and air-dried (0.030 g, 28 %). Anal.Found: C, 60.37; H, 5.55; N, 1.20. Anal.Calcd. for $C_{60}H_{65}AgNO_2P_2Pt$: C, 60.20; H, 5.47; N, 1.17. IR (ATR, cm^{-1}): 1598 (w, υ(N-C)), 1583 (w, υ(N-C)), 1572 (m, υ(N-C)), 1535 (s, υ(N-C)), 1086 (s, υ(ClO_4^-)), 619 (s, υ(ClO_4^-)), 542 (s, υ(P-C)), 511 (s, υ(P-C)), 503 (s, υ(P-C)). ^1H NMR (400.132 MHz, CD_2Cl_2, 293K, see Chart 1 for the H numbering scheme): δ = 7.97 (1H, ddd, $^3J_{Ha\text{-}Hb}$ = 7.8 Hz, $^4J_{Ha\text{-}P}$ = 4.3 Hz, $^4J_{Ha\text{-}Hc}$ = 1.2 Hz, Ha), 7.92 (1H, d, $^3J_{H6\text{-}H7}$ = 7.9 Hz, H6), 7.80 (1H, t, $^3J_{H7\text{-}H6}$ = 7.9 Hz, H7, overlapped with H9), 7.78 (2H, d, $^3J_{H9\text{-}H10}$ = 7.7 Hz, H9, overlapped with H7), 7.70 (1H, dd, $^3J_{H5\text{-}H4}$ = 7.7 Hz, $^4J_{H5\text{-}H3}$ = 1.2 Hz, H5), 7.67 (1H, tpt, $^3J_{Hb\text{-}Ha}$ = $^3J_{Hb\text{-}Hc}$ = 7.8 Hz, $^4J_{H\text{-}H}$ = 1.2 Hz, Hb), 7.63-7.28 (27H, m, H-PPh_2, H-PPh_3, Hc y H11), 7.24 (2H, t, $^3J_{H10\text{-}H9}$ = $^3J_{H10\text{-}H11}$ = 7.7 Hz,

H10), 7.13 (1H, td, $^3J_{H4-H5}$ = $^3J_{H4-H3}$ = 7.7 Hz, $^4J_{H4-H2}$ = 1.0 Hz, H4), 7.00 (1H, m, Hd, overlapped with H8), 6.94 (1H, d, $^3J_{H8-H7}$ = 7.3 Hz, H8), 6.70 (1H, td, $^3J_{H3-H2}$ = $^3J_{H3-H4}$ = 7.7 Hz, $^4J_{H3-H5}$ = 1.2 Hz, H3), 6.38 (1H, ddd, $^3J_{H2-H3}$ = 7.7 Hz, $^4J_{H2-P}$ = 3.5 Hz, $^4J_{H2-H4}$ = 1.0 Hz, $^3J_{H2-Pt}$ = 55.6 Hz, H2) ppm. $^{31}P\{^1H\}$ NMR (161.923 MHz, CD_2Cl_2, 293K): δ = 16.1 (d, $^1J_{P-Ag}$ = 804.6 Hz, $^1J_{P-Ag}$ = 701.6 Hz), 12.7 (s, $^1J_{P-Pt}$ = 4388 Hz) ppm. $^{13}C\{^1H\}$ NMR with HSQC y HMBC (100.624 MHz, CD_2Cl_2, 293K, see Chart 1 for the C numbering scheme): δ = 171.4 (s, COO), 164.7 (s, C14), 159.3 (s, C13), 146.1 (s, C15), 140.7 (s, C7), 138.8 (s, C12), 136.8 (d, $^3J_{C-P}$ = 6.0 Hz, C2), 134.8 (d, $^2J_{C-P}$ = 12.2 Hz, *o*-PPh_2), 134.1 (m, Cd, Ca y *o*-PPh_3), 132.8 (d, $^4J_{C-P}$ = 2.6 Hz, *p*-PPh_2), 132.1 (m, Cb y Cc), 131.9 (d, $^4J_{C-P}$ = 2.5 Hz, *p*-PPh_3), 131.3 (s, C1), 131.0 (s, C11), 129.9 (m, C3 y *m*-PPh_3), 129.6 (d, $^3J_{C-P}$ = 12.0 Hz, *m*-PPh_2), 129.2 (s, C10), 128.6 (s, C9), 127.8 (d, $^1J_{C-P}$ = 58.5 Hz, Ce), 127.0 (d, $^1J_{C-P}$ = 62.9 Hz, *q*-PPh_2), 125.3 (s, C5), 125.1 (s, C4), 124.5 (d, $^4J_{C-P}$ = 3.8 Hz, C8), 118.0 (d, $^4J_{C-P}$ = 2.1 Hz, C6) ppm. MS MALDI+ DCTB: m/z = 1569.3 [{Pt(CNC-H){$PPh_2(C_6H_4$-*o*-COO)}}$_2$Ag]$^+$, 1100.2 [{Pt(CNC-H){$PPh_2(C_6H_4$-*o*-COO)}Ag(PPh_3)]$^+$.

Preparation of the mixture 4*. To a solution of [Pt(CNC-H){$PPh_2(C_6H_4$-*o*-O)}] (**2**) (0.100 g, 0.142 mmol) in 30 mL CH_2Cl_2 at room temperature [Ag($OClO_3$)(PPh_3)] (0.067 g, 0.142 mmol) was added. After 1 h of stirring in the dark, the resulting yellow solution was evaporated to dryness. The resulting orange-yellow solid was extracted with 10 mL of *n*-hexane, filtered and air-dried (0.063 g, 42%). Anal.Found: C, 60.47; H, 5.48; N, 1.11. Anal.Calcd. for $C_{59}H_{65}AgNOP_2Pt$: C, 60.62; H, 5.60; N, 1.20. IR (ATR, cm^{-1}): 1598 (w, υ(N-C)), 1579 (w, υ(N-C)), 1548 (s, υ(N-C)), 1090 (s, υ(ClO_4^-)), 616 (s, υ(ClO_4^-)), 516 (s, υ(P-C)), 503 (s, υ(P-C)), 487 (s, υ(P-C)). ^1H NMR (400.132 MHz, CD_2Cl_2, 293K, see Chart 1 for the H numbering scheme): δ = 7.90 (1H, t, $^3J_{H7-H6}$ = $^3J_{H7-H8}$ = 7.6 Hz, H7), 7.84 (2H, d, $^3J_{H9-H10}$ = 8.1 Hz, H9), 7.75 (4H, m, *o*-PPh_2), 7.67 (1H, d, $^3J_{H5-H4}$ = 7.7 Hz, H5), 7.43 (22H, m, H_{ar}), 7.07 (8H, m, H_{ar}), 6.89 (1H, td, $^3J_{H3-H2}$ = $^3J_{H3-H4}$ = 7.5 Hz, $^4J_{H3-H5}$ = 1.4 Hz, H3), 6.55 (1H, t, $^3J_{Hc-Hd}$ = $^3J_{Hc-Hb}$ = 6.9 Hz, Hc), 6.01 (1H, m, Hd) ppm. $^{31}P\{^1H\}$ NMR (161.923 MHz, CD_2Cl_2, 213K): δ = 19.0 (s, $^1J_{P-Pt}$ = 4177 Hz), 13.7 (d, $^1J_{P-Ag}$ = 894.4 Hz, $^1J_{P-Ag}$ = 697.3 Hz), 12.6 (d, $^1J_{P-Ag}$ = 576.4 Hz, $^1J_{P-Ag}$ = 501.9 Hz) ppm. MS ESI+: m/z = 1072.13 [{Pt(CNC-H){$PPh_2(C_6H_4$-*o*-O)}}Ag(PPh_3)]-H]$^+$.

Preparation of the complex [{Pt(CNC-H){PPh$_2$(C$_6$H$_4$-*o*-COO)}}$_2$Ag](ClO$_4$) (5). To a solution of [Pt(CNC-H){PPh$_2$(C$_6$H$_4$-*o*-COO)}] (**1**) (0.070 g, 0.096 mmol) in 30 mL of acetone at room temperature, AgClO$_4$ (0.010 g, 0.048 mmol) was added. After 4 h of stirring in the dark, the resulting yellow solution was evaporated to dryness. The resulting yellow solid was extracted with 10 mL of *n*-hexane, filtered and air-dried (0.029 g, 36 %). Anal.Found: C,55.95; H, 4.98; N, 1.76. Anal.Calcd. for $C_{78}H_{82}AgN_2O_4P_2Pt_2$: C, 56.05; H, 4.94; N, 1.68. IR (ATR, cm^{-1}): 1699 (w, υ(C=O)), 1599 (w, υ(N-C)), 1588 (w, υ(N-C)), 1572 (m, υ(N-C)), 1538 (s, υ(N-C)), 1087 (s, υ(ClO_4^-)), 623 (s, υ(ClO_4^-)), 536 (s, υ(P-C)), 511 (s, υ(P-C)). ^{1}H NMR (400.132 MHz, CD_2Cl_2, 293K, see Chart 1 for the H numbering scheme): δ = 7.96 (2H, ddd, $^3J_{Ha\text{-}Hb}$ = 7.9 Hz, $^4J_{Ha\text{-}P}$ = 4.4 Hz, $^4J_{Ha\text{-}Hc}$ = 1.2 Hz, Ha), 7.63 (20H, m, H9, Hb, H5, *o*-PPh$_2$, *p*-PPh$_2$ y H6), 7.52 (8H, m, *m*-PPh$_2$), 7.48 (2H, tpt, $^3J_{Hc\text{-}Hd}$ = $^3J_{Hc\text{-}Hb}$ = 7.7 Hz, $^4J_{H\text{-}H}$ = 1.2 Hz, Hc), 7.40 (2H, t, $^3J_{H7\text{-}H8}$ = $^3J_{H7\text{-}H6}$ = 7.9 Hz, H7), 7.20 (2H, tt, $^3J_{H11\text{-}H10}$ = 7.7 Hz, H11, overlapped with H4), 7.16 (2H, td, $^3J_{H4\text{-}H5}$ = $^3J_{H4\text{-}H3}$ = 7.7 Hz, $^4J_{H4\text{-}H2}$ = 1.0 Hz, H4, overlapped with H11), 7.02 (4H, t, $^3J_{H10\text{-}H9}$ = $^3J_{H10\text{-}H11}$ = 7.7 Hz, H10), 6.89 (2H, d, $^3J_{H8\text{-}H7}$ = 7.9 Hz, H8, overlapped with Hd), 6.85 (2H, m, Hd, overlapped with H8), 6.72 (2H, td, $^3J_{H3\text{-}H4}$ = $^3J_{H3\text{-}H2}$ = 7.7 Hz, $^4J_{H3\text{-}H5}$ = 1.5 Hz, H3), 6.53 (2H, ddd, $^3J_{H2\text{-}H3}$ =7.7 Hz, $^4J_{H2\text{-}P}$ = 3.4 Hz, $^4J_{H2\text{-}H4}$ = 1.0 Hz, $^3J_{H2\text{-}Pt}$ = 55.0 Hz, H2) ppm. ^{31}P{^{1}H} NMR (161.923 MHz, CD_2Cl_2, 293K): δ = 11.7 (s, $^1J_{P\text{-}Pt}$ = 4382 Hz) ppm. ^{13}C{^{1}H} NMR with HSQC y HMBC (100.624 MHz, CD_2Cl_2, 293K, see Chart 1 for the C numbering scheme): δ = 170.3 (s, COO), 164.0 (s, C14), 159.7(s, C13), 146.3 (s, C15), 140.0 (s, C7), 138.5 (s, C12), 138.1 (s, Cf), 137.2 (d, $^3J_{C\text{-}P}$ = 5.9 Hz, C2), 134.5 (d, $^2J_{C\text{-}P}$ = 11.9 Hz, *o*-PPh$_2$), 133.8 (d, $^2J_{C\text{-}P}$ = 5.5 Hz, Cd), 133.6 (d, $^3J_{C\text{-}P}$ = 8.7 Hz, Ca), 132.8 (d, $^4J_{C\text{-}P}$ = 2.8 Hz, *p*-PPh$_2$), 131.9 (d, $^4J_{C\text{-}P}$ = 2.4 Hz, Cb), 131.6 (d, $^3J_{C\text{-}P}$ = 9.8 Hz, Cc), 131.4 (s, C1), 130.2 (s, C11), 130.0 (s, C3), 129.7 (d, $^3J_{C\text{-}P}$ = 12.1 Hz, *m*-PPh$_2$), 128.5 (s, C10), 128.4 (s, C9), 127.2 (d, $^1J_{C\text{-}P}$ = 62.7 Hz, *q*-PPh$_2$), 127.0 (d, $^1J_{C\text{-}P}$ = 59.8 Hz, Ce), 125.3 (d, $^4J_{C\text{-}P}$ = 3.9 Hz, C8), 125.1 (s, C5), 125.0 (s, C4), 117.9 (d, $^4J_{C\text{-}P}$ = 2.2 Hz, C6) ppm. MS MALDI+ DCTB: m/z = 1569.4 [{Pt(CNC-H){PPh$_2$(C$_6$H$_4$-*o*-COO)}}$_2$Ag]$^+$.

Preparation of the complex [{Pt(CNC-H){PPh$_2$(C$_6$H$_4$-*o*-O)}}$_2$Ag](ClO$_4$) (6). To a solution of [Pt(CNC-H){PPh$_2$(C$_6$H$_4$-*o*-O)}] (**2**) (0.100 g, 0.142 mmol) in 30 mL of acetone at room temperature AgClO$_4$ (0.015 g, 0.071 mmol) was added.

After 1 h of stirring in the dark, the resulting yellow solution was evaporated to dryness. The resulting orange-yellow solid was extracted with 10 mL of *n*-hexane, filtered and air-dried (0.073 g, 73 %). Anal.Found: C,56.39; H, 5.22; N, 1.73. Anal.Calcd. for $C_{76}H_{82}AgN_2O_2P_2Pt_2$: C, 56.51; H, 5.12; N, 1.73. IR (ATR, cm^{-1}): 1596 (w, υ(N-C)), 1579 (w, υ(N-C)), 1552 (w, υ(N-C)), 1090 (s, υ(ClO_4^-)), 619 (s υ(ClO_4^-)), 531 (s, υ(P-C)), 507 (s, υ(P-C)), 487 (s, υ(P-C)). ^{1}H NMR (400.132 MHz, CD_2Cl_2, 293K, see Chart 1 for the H numbering scheme): δ = 7.77 (2H, d, $^3J_{H6-H7}$ = 8.1 Hz, H6), 7.65 (16H, m, H7, H5, *o*-PPh_2), 7.46 (4H, t, $^3J_{Hp-Hm}$ = 6.75 Hz, *p*-PPh_2), 7.34 (8H, m, *m*-PPh_2), 7.27 (2H, tt, $^3J_{H11-H10}$ = 7.8 Hz, $^4J_{H11-H9}$ = 1.1 Hz, H11), 7.16 (2H, td, $^3J_{H4-H3}$ = $^3J_{H4-H5}$ = 7.6 Hz, $^4J_{H4-H2}$ = 1.0 Hz, H4 overlapped with H8), 7.12 (2H, d, $^3J_{H8-H7}$ = 7.7 Hz, H8 overlapped with H4), 7.0 (10H, m, H10, Ha, H2 y Hc), 6.82 (2H, td, $^3J_{H3-H4}$ = $^3J_{H3-H2}$ = 7.6 Hz, $^4J_{H3-H5}$ = 1.0 Hz, H3), 6.55 (2H, tpt, $^3J_{Hb-Ha}$ = $^3J_{Hb-Hc}$ = 7.3 Hz, J_{H-H} = 2.0 Hz, Hb), 5.65 (2H, m, Hd) ppm. ^{31}P{^{1}H} NMR (161.923 MHz, CD_2Cl_2, 293K): δ = 20.3 (s, $^1J_{P-Pt}$ = 4216 Hz) ppm. ^{13}C{^{1}H} NMR with HSQC y HMBC (100.624 MHz, CD_2Cl_2, 293K, see Chart 1 for the C numbering scheme): δ = 174.1 (s, Cf), 164.9 (s, C14), 160.8 (s, C13), 147.4 (s, C15), 140.8 (s, C7), 138.5 (d, $^3J_{C-P}$ = 5.8 Hz, C2), 137.6 (s, C12), 134.4 (s, Ca o Cc), 134.0 (d, $^2J_{C-P}$ = 11.5 Hz, *o*-PPh_2), 132.3 (d, $^4J_{C-P}$ = 2.4 Hz, *p*-PPh_2), 132.2 (s, Ca o Cc), 130.6 (d, $^4J_{C-P}$ = 1.8 Hz, C3), 130.3 (s, C11), 129.5 (d, $^3J_{C-P}$ = 12.1 Hz, *m*-PPh_2), 129.0 (s, C1), 128.3 (s, C9), 128.2 (s, C10), 126.0 (s, C4), 125.8 (s, C5), 124.9 (d, $^4J_{C-P}$ = 3.6 Hz, C8), 119.9 (d, $^2J_{C-P}$ = 8.9 Hz, Cb), 116.9 (s, Ce) ppm. MS ESI+: m/z = 1513.2 [{Pt(CNC-H){$PPh_2(C_6H_4$-*o*-O)}}$_2$Ag]$^+$.

Preparation of the complex [{Pt(CNC-H){$PPh_2(C_6H_4$-*o*-COO)}}Au(PPh_3)](ClO_4) (7). Over a solution of [Au(Cl)(PPh_3)] (0.064 g, 0.130 mmol) in 20 mL of THF kept under Ar atmosphere, $AgClO_4$ (0.027 g, 0.130 mmol) was added. The resulting suspensión was stirred for 1h in absence of light at -65ºC. Then, [Pt(CNC-H){$PPh_2(C_6H_4$-*o*-COO)}] (**1**) (0.086 g, 0.120 mmol) was added along 10 mL of THF and the suspension was stirred for one more hour in absence of light until it reached a temperature of -15ºC. The AgCl solid was then filtered off under Ar atmosphere and the resulting yellow solution was evaporated to dryness. The resulting yellow solid was extracted with 10 mL of *n*-hexane, filtered and air-dried (0.105 g, 68 %). Anal.Found: C, 55.94; H, 4.89; N, 1.86. Anal.Calcd. for $C_{60}H_{65}AuNO_2P_2Pt$: C, 56.03; H, 5.09; N, 1.09. IR (ATR, cm^{-1}):

1601 (w, υ(N-C)), 1582 (w, υ(N-C)), 1568 (s, υ(N-C)), 1551 (m, υ(N-C)), 1084 (s, υ(ClO_4^-)), 618 (s, υ(ClO_4^-)), 541 (s, υ(P-C)), 509 (s, υ(P-C)). ^{1}H NMR (400.132 MHz, CD_2Cl_2, 293K, see Chart 1 for the H numbering scheme): δ = 8.03 (2H, m, H6 y H7), 7.93 (1H, ddd, $^3J_{Ha-Hb}$ = 7.8 Hz, $^4J_{Ha-Hc}$ = 4.2 Hz, $^4J_{Ha-P}$= 1.2 Hz, Ha), 7.77 (1H, ddd, $^3J_{H5-H4}$ = 8.1 Hz, $^3J_{H5-H3}$ = 1.1 Hz, H5), 7.68 (3H, m, H9 y Hb), 7.58 (10H, m, *o*-PPh_2, *p*-PPh_2, *p*-PPh_3, Hc), 7.46 (16H, m, *o*-PPh_3, *m*-PPh_3, *m*-PPh_2), 7.37 (1H, tt, $^3J_{H11-H10}$ = 7.5 Hz, $^4J_{H11-H9}$ = 1.2 Hz, H11), 7.13 (4H, m, H4, H8 y H10), 7.01 (1H, m, Hd), 6.71 (1H, td, $^3J_{H3-H2}$ = $^3J_{H3-H4}$ = 7.8 Hz, $^4J_{H3-H5}$ = 1.1 Hz, H3), 6.39 (1H, ddd, $^3J_{H2-H3}$ = 7.9 Hz, $^4J_{H2-P}$ = 3.1 Hz, $^4J_{H2-H4}$ = 1.0 Hz, H2) ppm. $^{31}P\{^1H\}$ NMR (161.923 MHz, CD_2Cl_2, 293K): δ = 26.7 (s, P(Au)), 13.3 (s, $^1J_{P-Pt}$ = 4339 Hz, P(Pt)) ppm. $^{13}C\{^1H\}$ NMR with HSQC y HMBC (100.624 MHz, CD_2Cl_2, 293K, see Chart 1 for the C numbering scheme): δ = 171.4 (s, COO), 164.3 (s, C14), 160.0 (s, C13), 146.5 (s, C15), 138.3 (s, C12), 136.8 (s, C2), 134.8 (d, $^2J_{C-P}$ = 12.1 Hz, *o*-PPh_2), 134.5 (d, $^2J_{C-P}$ = 7.7 Hz, Cd), 134.3 (d, $^2J_{C-P}$ = 13.5 Hz, *o*-PPh_3(Au)), 133.8 (d, $^3J_{C-P}$ = 7.6 Hz, Ca), 132.9 (m, *p*-PPh_3 (Au), *p*-PPh_2), 132.4 (d, $^3J_{C-P}$ = 8.0 Hz, Cc), 131.9 (s, Cb), 130.5 (s, C11), 130.0 (s, C3), 129.8 (d, $^3J_{C-P}$ = 12.3 Hz, *m*-PPh_3(Au)), 129.7 (d, $^3J_{C-P}$ = 11.6 Hz, *m*-PPh_2), 129.5 (s, C9), 129.0 (s, C10), 126.7 (d, $^1J_{C-P}$ = 63.5 Hz, *q*-PPh_2), 125.3 (s, C5 y C8), 124.6 (s, C4), 117.8 (s, C6) ppm. MS MALDI+ DCTB: m/z = 1190.6 [{Pt(CNC-H){PPh_2(C_6H_4-*o*-COO)}}Au(PPh_3)]+H]$^+$.

Preparation of the complex [{Pt(CNC-H){PPh_2(C_6H_4-*o*-O)}}Au(PPh_3)](ClO_4) (8). Over a solution of [Au(Cl)(PPh_3)] (0.068 g, 0.140 mmol) in 20 mL of THF kept under Ar atmosphere, $AgClO_4$ (0.029 g, 0.140 mmol) was added. The resulting suspensión was stirred for 1h in absence of light at -65ºC. Then, [Pt(CNC-H){PPh_2(C_6H_4-*o*-O)}] (**2**) (0.088 g, 0.125 mmol) was added along 10 mL of THF and the suspension was stirred for one more hour in absence of light until it reached a temperature of -15ºC. The AgCl solid was then filtered off under Ar atmosphere and the resulting yellow solution was evaporated to dryness. The resulting yellow solid was extracted with 10 mL of *n*-hexane, filtered and air-dried (0.115 g, 73 %). Anal.Found: C, 56.12; H, 4.99; N, 1.43. Anal.Calcd. for $C_{59}H_{65}AgNOP_2Pt$: C, 56.32; H, 5.21; N, 1.11. IR (ATR, cm^{-1}): 1599 (w, υ(N-C)), 1580 (m, υ(N-C)), 1561 (w, υ(N-C)), 1552 (w, υ(N-C)), 1082 (s, υ(ClO_4^-)), 618 (s, υ(ClO_4^-)), 539 (s, υ(P-C)), 498 (s, υ(P-C)). ^{1}H NMR (400.132 MHz, CD_2Cl_2,

293K, see Chart 1 for the H numbering scheme): δ = 7.93 (2H, m, H7 y H6 overlapped), 7.85 (2H, d, $^{3}J_{H9-H10}$ = 7.1 Hz, H9), 7.72 (5H, m, H_{ar}), 7.53 (9H, m, H_{ar}), 7.39 (9H, m, H_{ar}), 7.19 (11H, m, H_{ar}), 6.93 (1H, td, $^{3}J_{H3-H2}$ = $^{3}J_{H3-H4}$ = 7.5 Hz, $^{4}J_{H3-H5}$ = 1.4 Hz, H3), 6.81 (1H, t, $^{3}J_{Ha-Hb}$ = $^{4}J_{Ha-P}$ = 7.3 Hz, Hb), 6.25 (1H, m, Hd) ppm. $^{31}P\{^{1}H\}$ RMN (161.923 MHz, CD_2Cl_2, 293K): 25.9 (s, P(Au)), 18.6 (s, $^{1}J_{P-Pt}$ = 4173 Hz, P(Pt)) ppm. MS MALDI+ DCTB: m/z =1161.6 [{Pt(CNC-H){$PPh_2(C_6H_4$-*o*-O)}}Au(PPh_3)]$^+$.

Preparation of the complex [{Pt(CNC-H){$PPh_2(C_6H_4$-*o*-COOH)}}{Pt(CNC-H){$PPh_2(C_6H_4$-*o*-COO)}}](ClO_4) (9). To a solution of [{Pt(CNC-H){$PPh_2(C_6H_4$-*o*-COO)}}$_2$Ag](ClO_4) (**5**) (0.041 g, 0.025 mmol) in 10 mL of CH_2Cl_2 at room temperature, 0.10 mL of a solution of HCl (0.24 M in MeOH, 0.025 mmol) were added. After 90 minutes of stirring in absence of light, the resulting yellow suspension was filtered off, to the yellow solution $MgSO_4$ was added and after 10 minutes of stirring was filtered off, and the solution was evaporated to dryness. The resulting yellow solid was extracted with 10 mL of *n*-hexane, filtered and air-dried (0.034 g, 89 %). Anal.Found: C, 60.05; H, 5.35; N, 1.64. Anal.Calcd. for $C_{78}H_{83}N_2O_4P_2Pt_2$: C, 59.88 H, 5.35; N, 1.79. IR (ATR, cm^{-1}): 1619 (w, υ(N-C)), 1578 (w, υ(N-C)), 1552 (w, υ(N-C)), 1084 (s, υ(ClO_4^-)), 620 (m, υ(ClO_4^-)), 539 (m, υ(P-C)), 509 (m, υ(P-C)), 437 (m, υ(P-C)).^{1}H NMR (400.132 MHz, CD_2Cl_2, 293K, see Chart 1 for the H numbering scheme): δ = 7.84 (2H, ddd, $^{3}J_{Ha-Hb}$= 8.0 Hz, $^{4}J_{Ha-P}$= 4.4 Hz, $^{4}J_{Ha-Hc}$= 1.2 Hz, Ha), 7.74 (2H, d , $^{3}J_{H9-H10}$= 7.6 Hz, H9), 7.69 (2H, dd, $^{3}J_{H5-H4}$= 7.6 Hz, $^{4}J_{H5-H3}$= 3.2 Hz, H5), 7.60 (12H, m, Hb, *o*-PPh_2, *p*-PPh_2, *m*-PPh_2 y H6), 7.49 (2H, t, $^{3}J_{H7-H6}$= $^{3}J_{H7-H8}$= 7.6 Hz, H7), 7.44 (2H, m, Hc), 7.16 (2H, td, $^{3}J_{H4-H5}$= $^{3}J_{H4-H3}$= 7.5 Hz, $^{4}J_{H4-H2}$= 1.0 Hz, H4, overlapped with H11), 7.12 (2H, t, $^{3}J_{H11-H10}$= $^{3}J_{H11-H9}$= 7.4 Hz, H11, overlapped with H4), 6.89 (2H, d, $^{3}J_{H8-H7}$= 7.9 Hz, H8, overlapped with Hd), 6.83 (2H, m, Hd, overlapped with H8), 6.75 (2H, td, $^{3}J_{H3-H4}$= $^{3}J_{H3-H2}$= 7.5 Hz, $^{4}J_{H3-H5}$= 1.4 Hz, H3), 6.50 (2H, ddd, $^{3}J_{H2-H3}$= 7.5 Hz, $^{4}J_{H2-P}$= 3.5 Hz, $^{4}J_{H2-H4}$= 1.0 Hz, $^{3}J_{H2-Pt}$= 51.0 Hz, H2) ppm.$^{31}P\{^{1}H\}$ NMR (161.923 MHz, CD_2Cl_2, 293K): δ = 12.6 (s, $^{1}J_{P-Pt}$ = 4339 Hz) ppm. $^{13}C\{^{1}H\}$ NMR with HSQC y HMBC (100.624 MHz, CD_2Cl_2, 293K, see Chart 1 for the C numbering scheme): δ = 169.1 (s, COO), 163.4 (s, C14), 160.3 (s, C13), 139.8 (s, C7), 138.4 (s, C12), 136.9 (d, $^{3}J_{C-P}$ = 5.8 Hz, C2), 133.9 (d, $^{3}J_{C-P}$ = 12.1 Hz, m-PPh_2), 133.7 (d, $^{4}J_{C-P}$= 4.2, C8), 133.0 (s, Ca), 129.8 (d, $^{3}J_{C-P}$= 10.8 Hz, Cc),

129.4 (s, C11), 129.3 (s, C3), 127.7 (s, Cd), 124.8 (s, C5), 124.7 (s, C4), 117.3 (s, C9) ppm. MS MALDI+ DIT: m/z = 731.4 [{Pt(CNC-H){$PPh_2(C_6H_4$-*o*-COOH)}}]$^+$.

Preparation of the complex [{Pt(CNC-H){$PPh_2(C_6H_4$-*o*-OH)}}{Pt(CNC-H){$PPh_2(C_6H_4$-*o*-O)}}](ClO_4) (10). To a solution of [{Pt(CNC-H){$PPh_2(C_6H_4$-*o*-O)}}$_2$Ag](ClO_4) (**6**) (0.072 g, 0.045 mmol) in 10 mL of CH_2Cl_2 at room temperature, 0.20 mL of a solution of HCl (0.24 M in MeOH, 0.045 mmol) were added. After 30 minutes of stirring in absence of light, the resulting yellow suspension was filtered off, to the yellow solution $MgSO_4$ was added and after 10 minutes of stirring was filtered off, and the solution was evaporated to dryness. The resulting yellow solid was extracted with 10 mL of *n*-hexane, filtered and air-dried (0.039 g, 58 %). Anal.Found: C, 60.47; H, 5.47; N, 1.91. Anal.Calcd. for $C_{76}H_{83}N_2O_2P_2Pt_2$: C, 60.51; H, 5.55; N, 1.86. IR (ATR, cm^{-1}): 1596 (w, υ(N-C)), 1578 (w, υ(N-C)), 1564 (w, υ(N-C)), 1098 (s, υ(ClO_4^-)), 623 (m, υ(ClO_4^-)), 530 (m, υ(P-C)), 509 (m, υ(P-C)), 488 (m, υ(P-C)).^{1}H NMR (400.132 MHz, CD_2Cl_2, 293K, see Chart 1 for the H numbering scheme): δ = 7.96 (2H, t, $^3J_{H7-H6}$= $^3J_{H7-H8}$= 7.7 Hz, H7), 7.83 (16H, m, *o*-PPh_2, H6 o H8 y H9), 7.62 (2H, d, $^3J_{H5-H4}$= 7.5 Hz, H5), 7.44 (20H, m, *m*-PPh_2, *p*-PPh_2, Ha, H6 o H8 y H10), 7.11 (2H, ddd, $^3J_{H2-H3}$= 7.7 Hz, $^4J_{H2-P}$= 3.4 Hz, $^4J_{H2-H4}$= 1.8 Hz, $^3J_{H2-Pt}$= 47.0 Hz, H2, overlapped with H4), 7.06 (2H, t, $^3J_{H4-H3}$= $^3J_{H4-H5}$= 7.5 Hz, H4, overlapped with H2), 6.95 (2H, tpt, $^3J_{H11-H9}$= $^3J_{H11-H10}$= 7.9 Hz, J_{H-H}= 1.0 Hz, H11), 6.83 (4H, m, H3 y Hc), 6.35 (2H, tpt, $^3J_{Hb-Ha}$= $^3J_{Hb-Hc}$= 7.4 Hz, J_{H-H}= 2.0 Hz, Hb), 5.79 (2H, m, Hd) ppm. ^{31}P{^{1}H} NMR (161.923 MHz, CD_2Cl_2, 293K): δ = 19.4 (s, $^1J_{P-Pt}$ = 4420 Hz) ppm.^{13}C{^{1}H} NMR with HSQC y HMBC (100.624 MHz, CD_2Cl_2, 293K, see Chart 1 for the C numbering scheme): δ = 173.9 (s, Cf), 165.0 (s, C14), 161.4 (s, C13), 146.2 (s, C15), 139.6 (s, C7), 139.0 (s, Ca), 138.2 (d, $^3J_{C-P}$= 7.5 Hz, C2), 133.8 (d, $^3J_{C-P}$= 12.7 Hz, *m*-PPh_2), 133.4 (s, C11), 133.0 (s, C9), 131.1 (s, Cc), 129.8 (d, $^4J_{C-P}$= 1.4 Hz, C3), 129.5 (d, $^4J_{C-P}$= 2.5 Hz, *p*-PPh_2), 129.3 (s, C5), 124.4 (s, C6 o C8), 123.3 (s, C4), 119.1 (d, $^2J_{C-P}$= 8.9 Hz, Cd), 117.2 (s, C1), 116.9 (s, Ce), 115.0 (s, Cb) ppm. MS MALDI+ DIT: m/z = 703.04 [{Pt(CNC-H){$PPh_2(C_6H_4$-*o*-OH)}}]$^+$.

2. IR, NMR and MS spectra of complexes

2.1. Spectra of complex [Pt(CNC-H){$PPh_2(C_6H_4$-*o*-COO)}] (1).

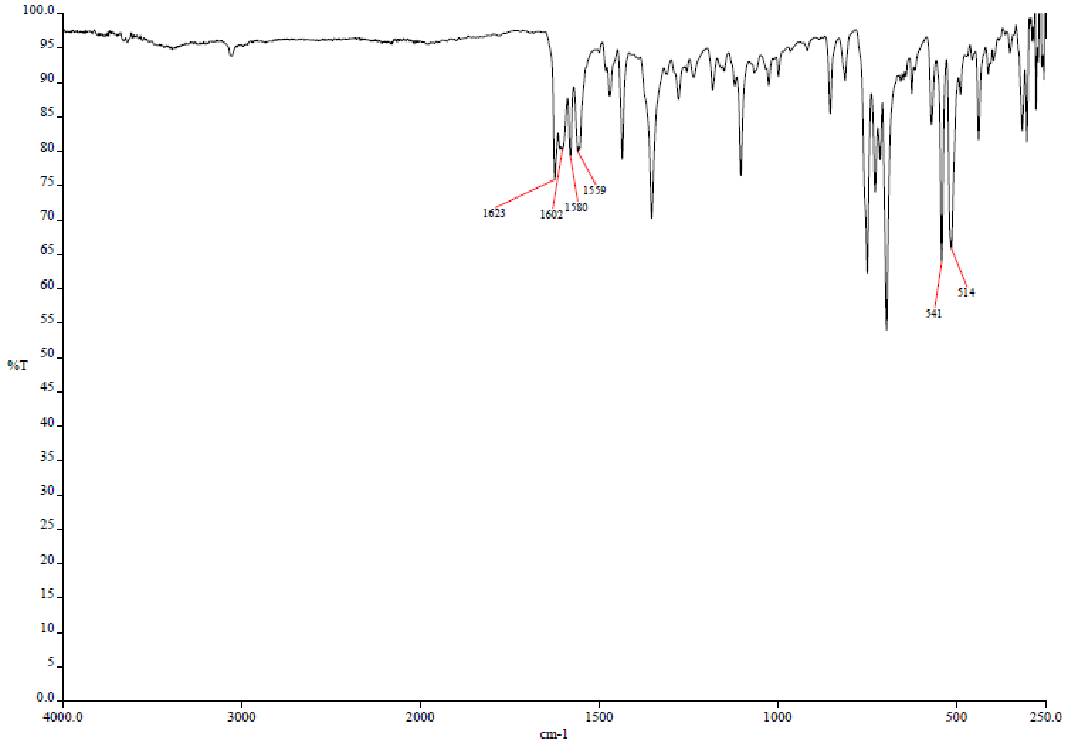

Figure S1. ATR-IR spectrum of complex **1**.

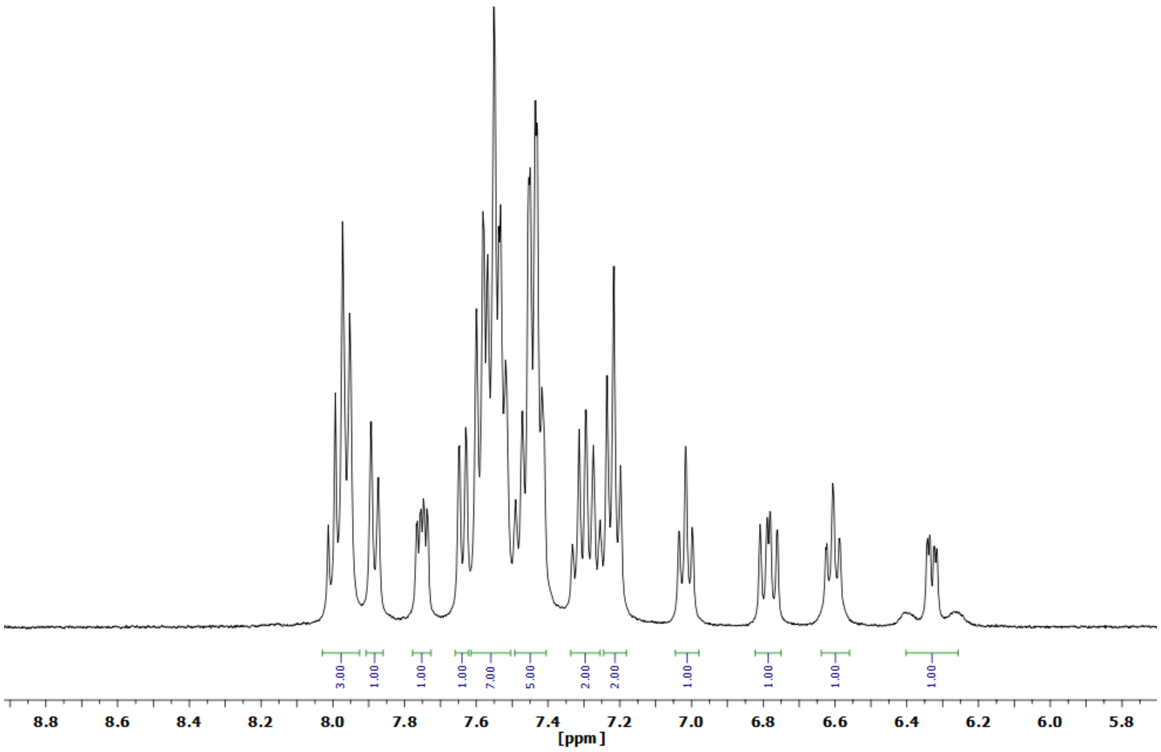

Figure S2. ^{1}H NMR spectrum (RT, CD_2Cl_2) of complex **1**.

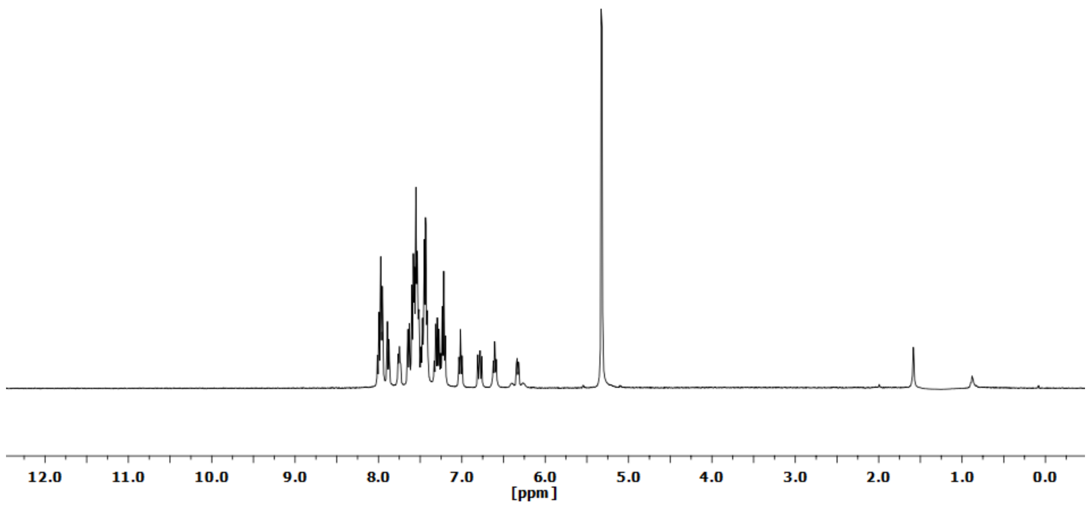

Figure S3. Full ^{1}H NMR spectrum (RT, CD_2Cl_2) of complex **1**.

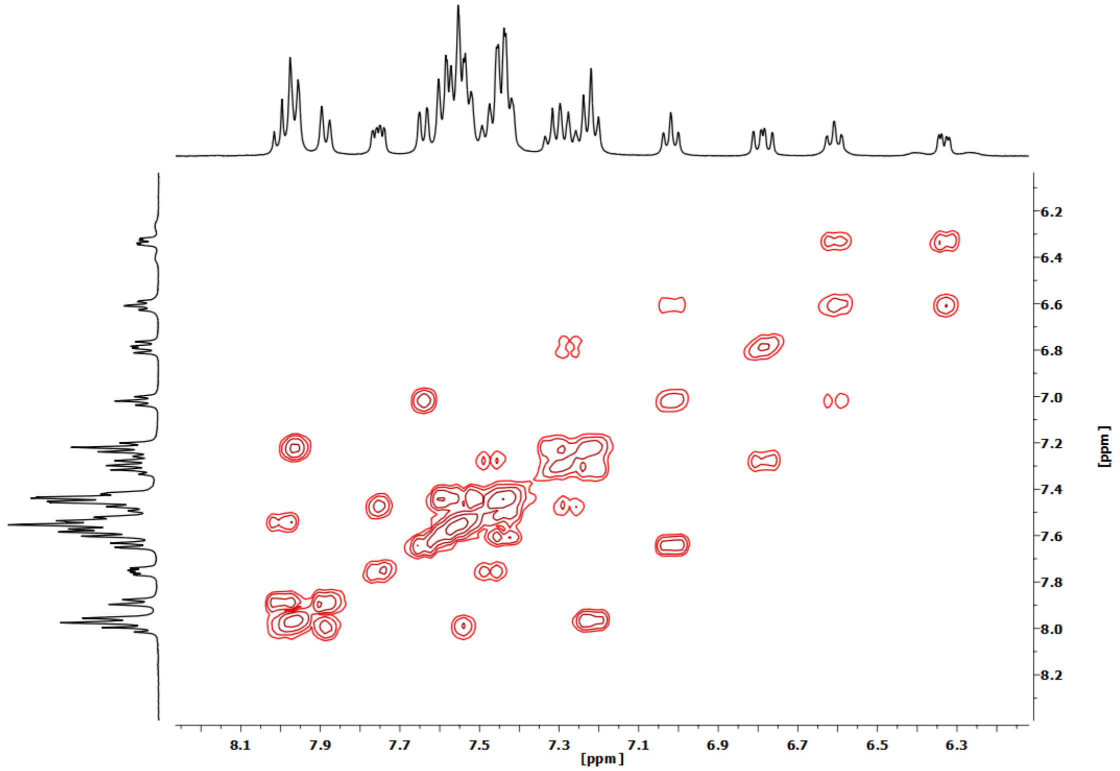

Figure S4. ^{1}H-^{1}H COSY NMR spectrum (RT, CD_2Cl_2) of complex **1**.

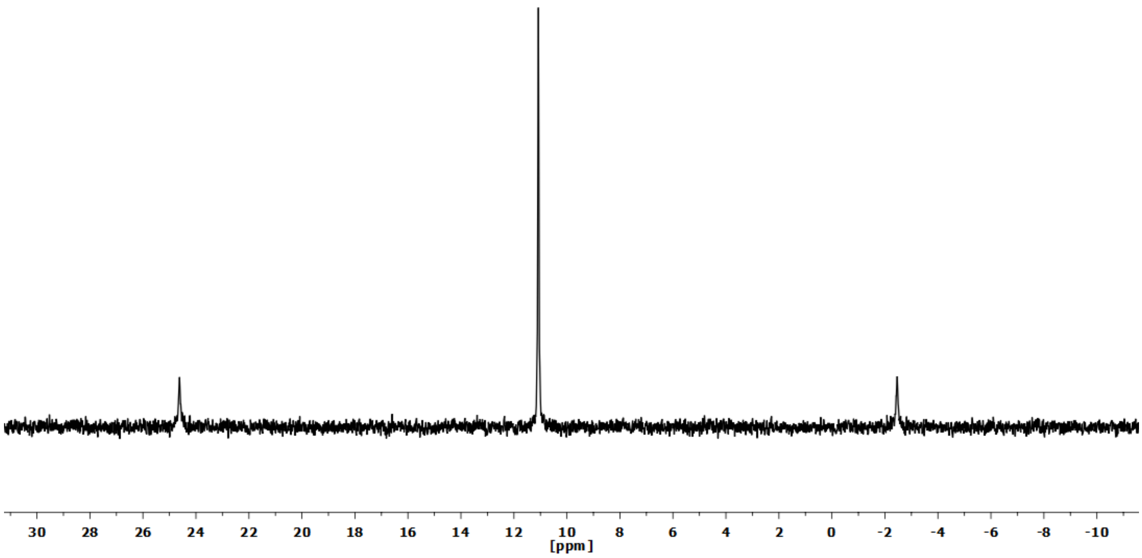

Figure S5. $^{31}P\{^1H\}$ NMR spectrum (RT, CD_2Cl_2) of complex **1**.

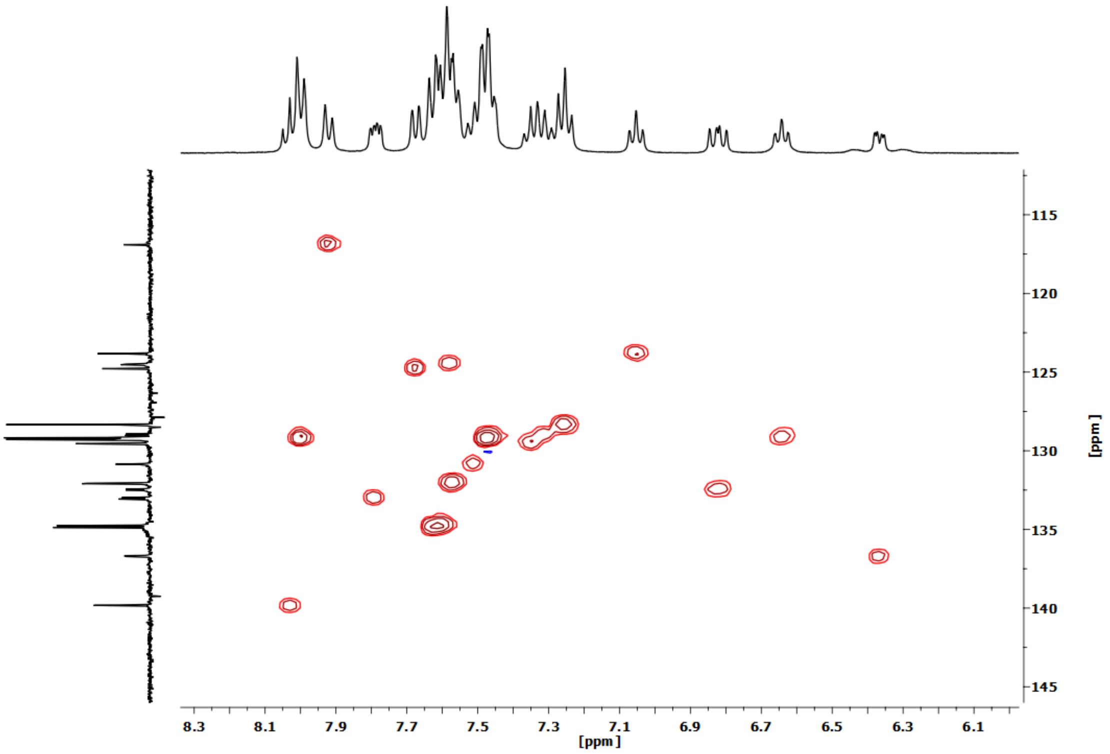

Figure S6. ^1H-^{13}C HSQC NMR spectrum (RT, CD_2Cl_2) of complex **1**.

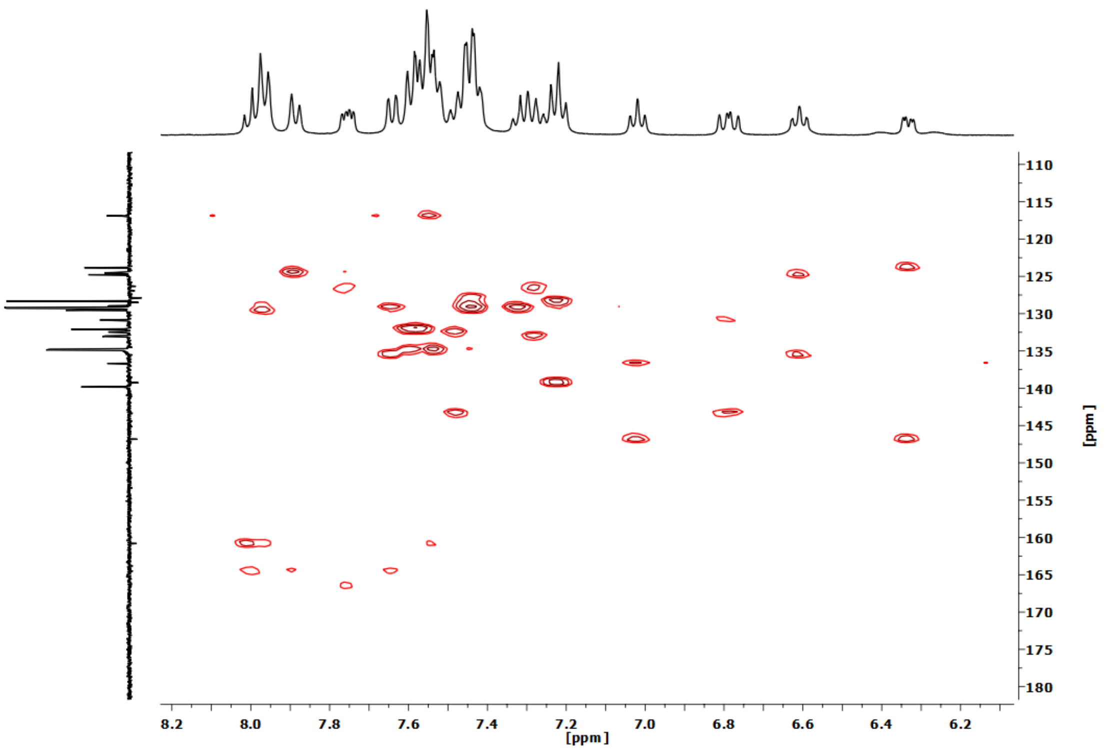

Figure S7. ^{1}H-^{13}C HMBC NMR spectrum (RT, CD_2Cl_2) of complex **1**.

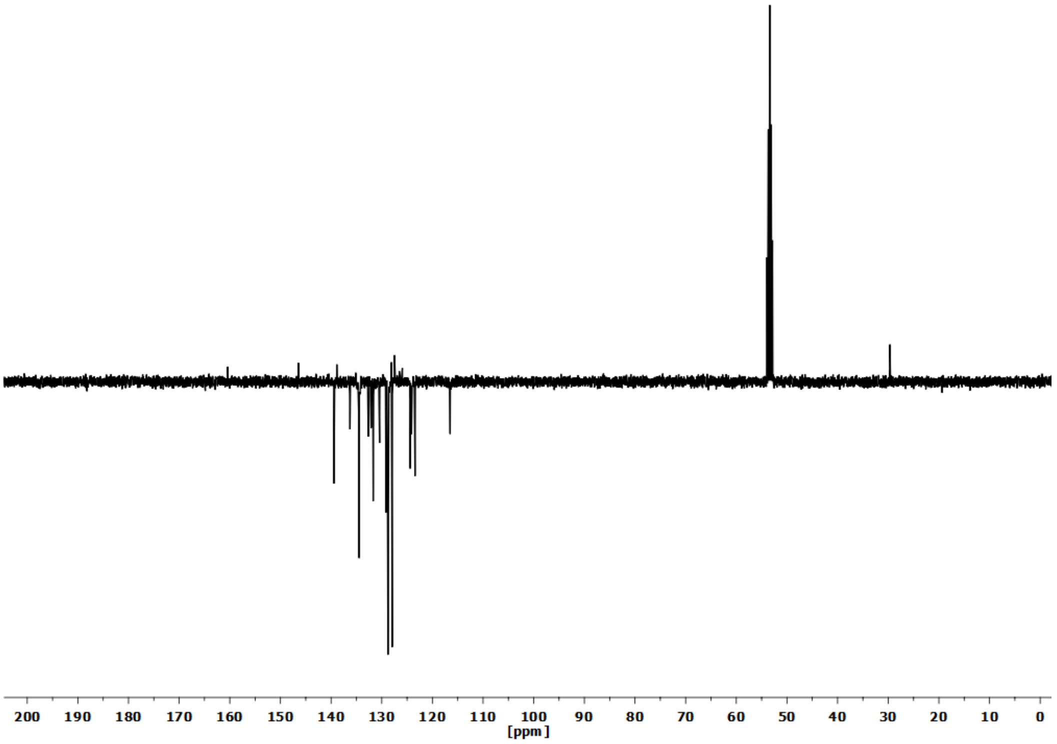

Figure S8. APT $^{13}C\{^{1}H\}$ NMR spectrum (RT, CD_2Cl_2) of complex **1**.

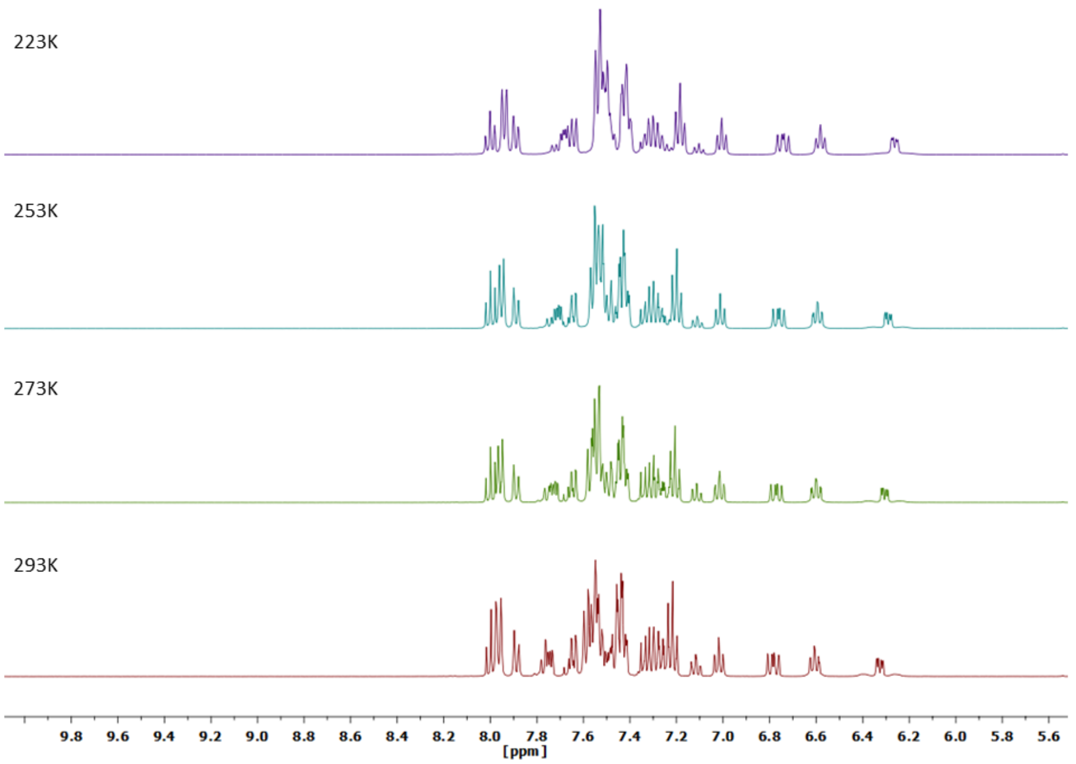

Figure S9. VT ^{1}H NMR spectrum (CD_2Cl_2) of reaction of complex **1**. Time lapse of measurements: 90 minutes.

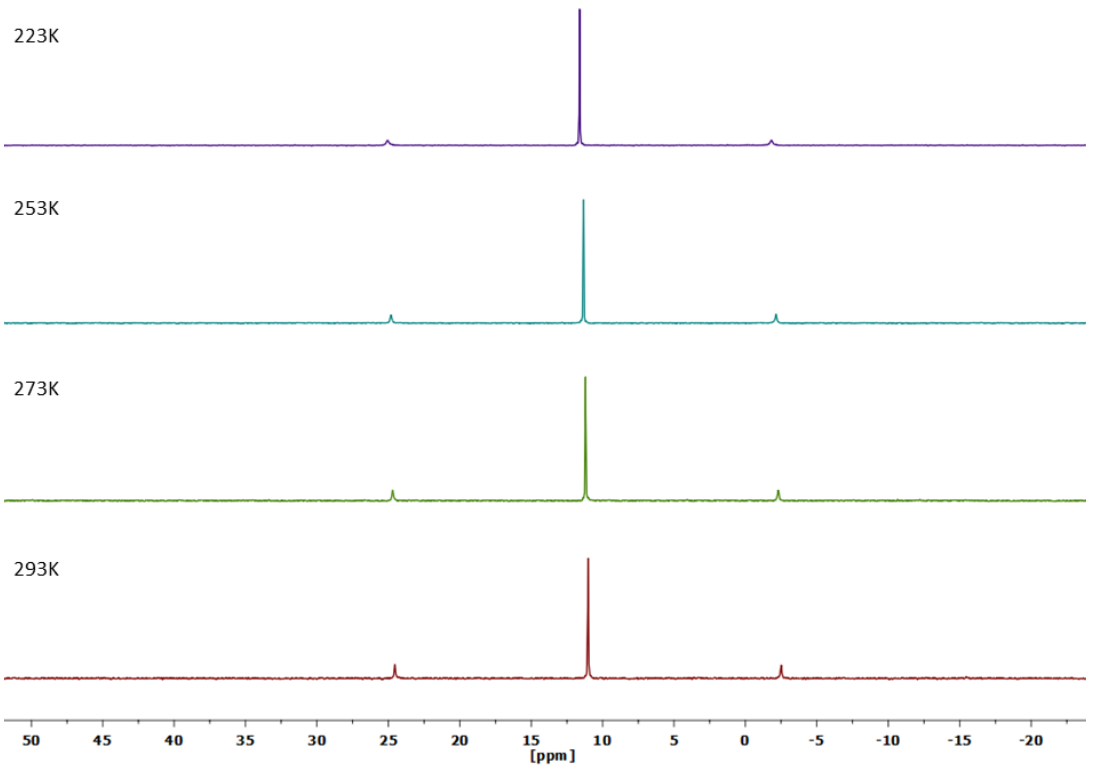

Figure S10. VT ^{31}P NMR spectrum (CD_2Cl_2) of reaction of complex **1**. Time lapse of measurements: 90 minutes.

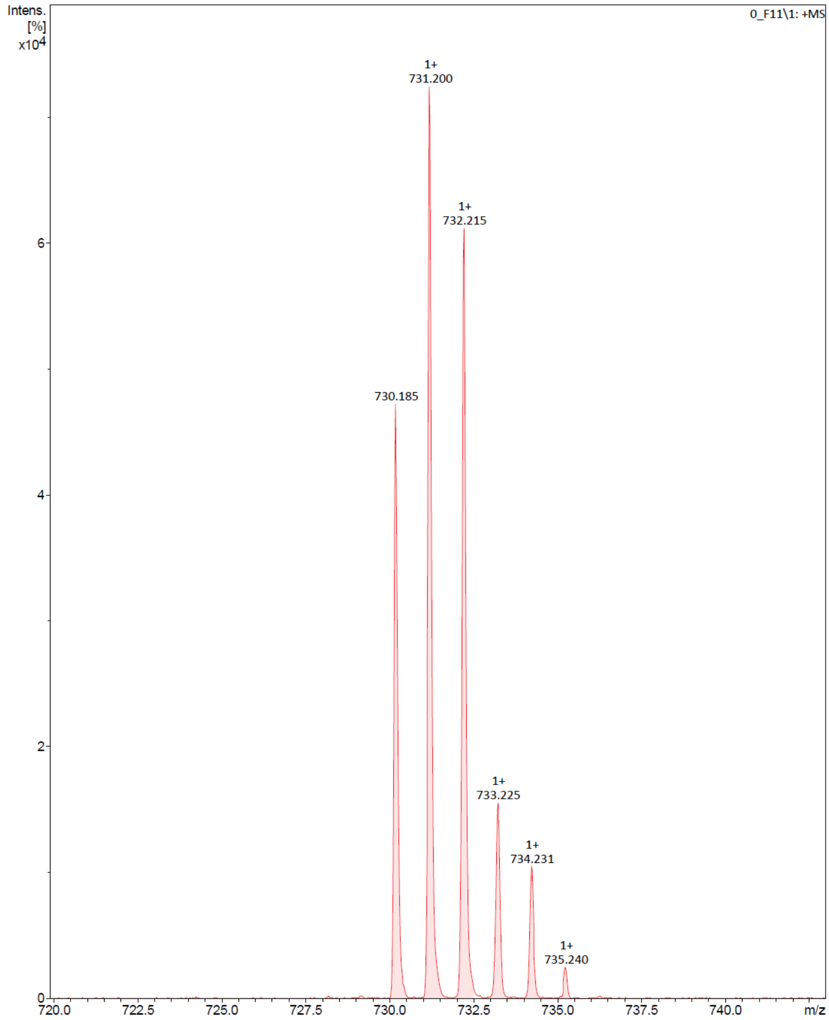

Figure S11. MS (MALDI+ DCTB) molecular peak of complex **1**.

2.2. Spectra of complex [Pt(CNC-H){PPh$_2$(C$_6$H$_4$-*o*-O)}] (2).

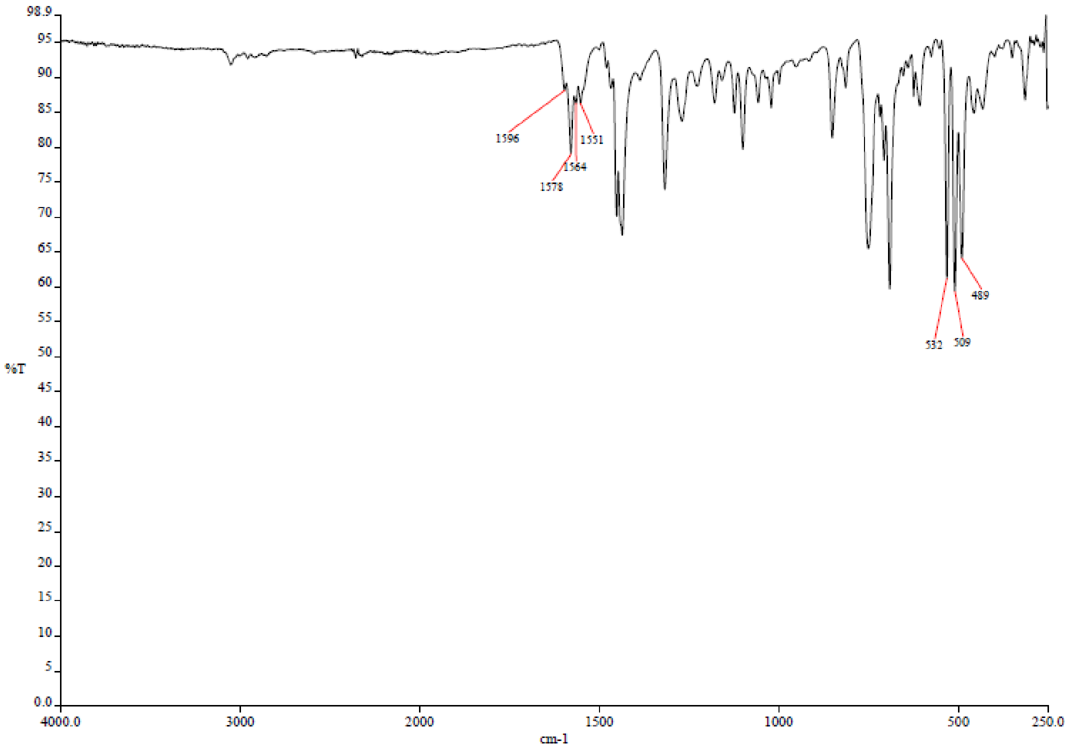

Figure S12. ATR-IR spectrum (RT, CD$_2$Cl$_2$) of complex **2**.

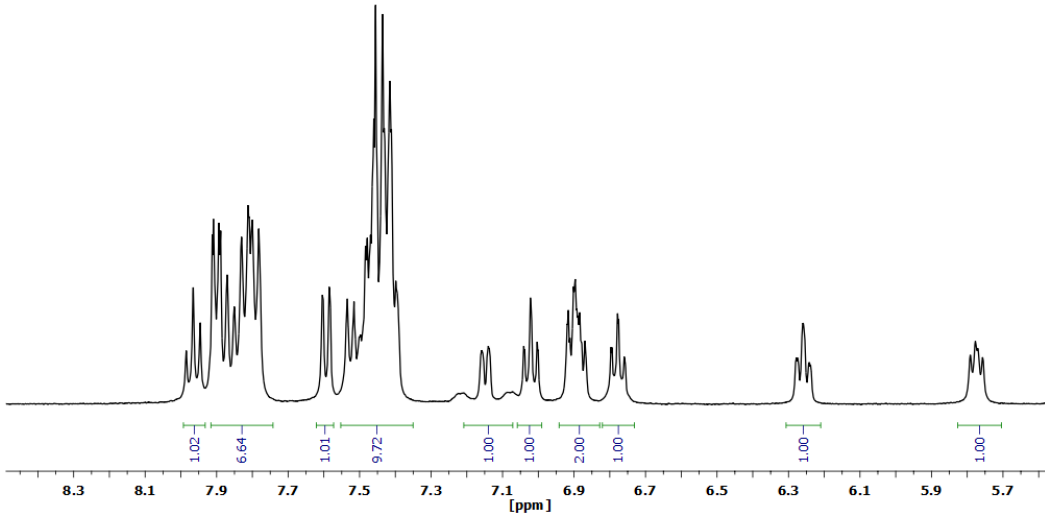

Figure S13. ^{1}H NMR spectrum (RT, CD$_2$Cl$_2$) of complex **2**.

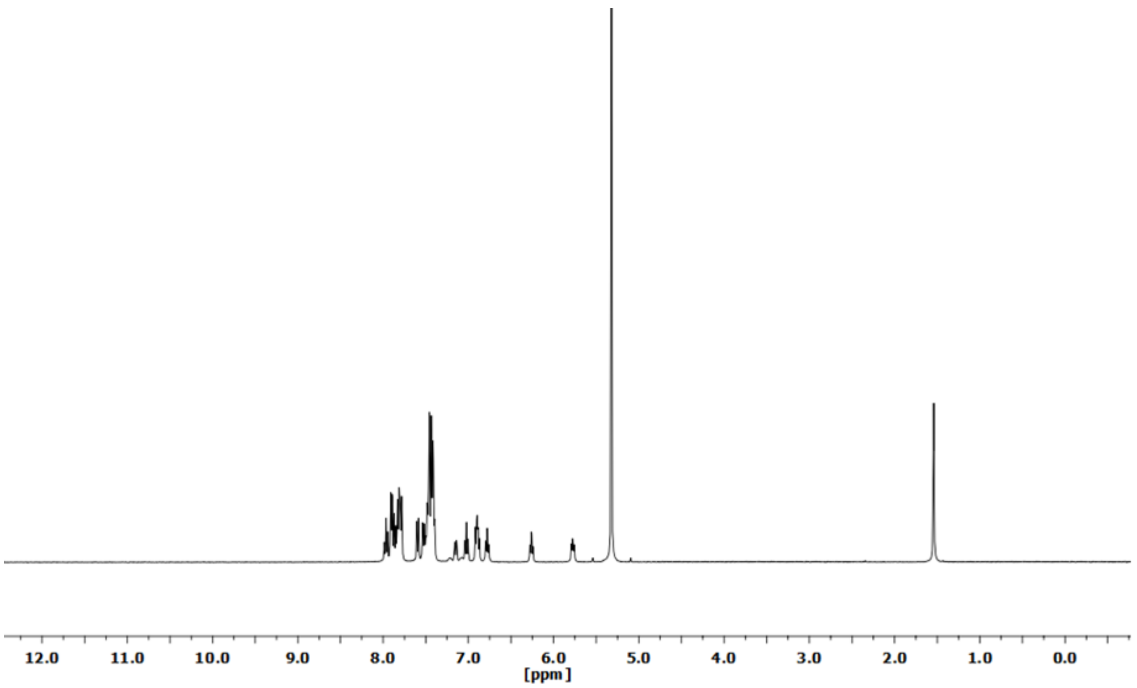

Figure S14. Full ^1H NMR spectrum (RT, CD_2Cl_2) of complex **2**.

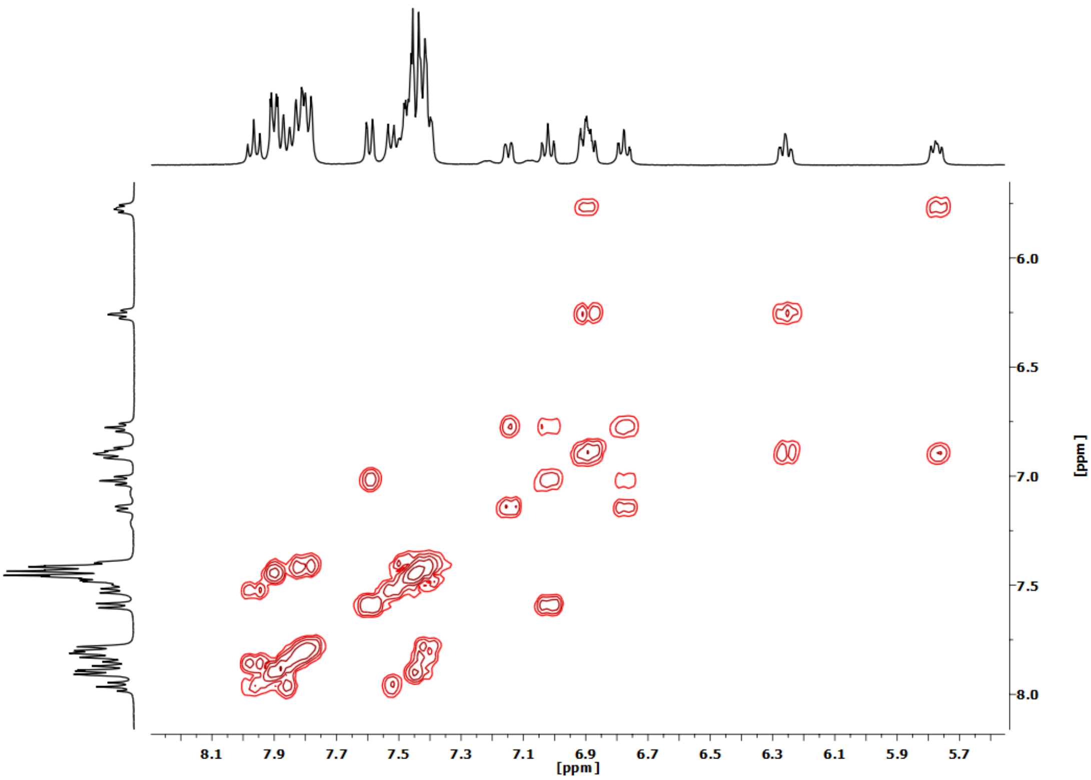

Figure S15. ^1H-^1H COSY NMR spectrum (RT, CD_2Cl_2) of complex **2**.

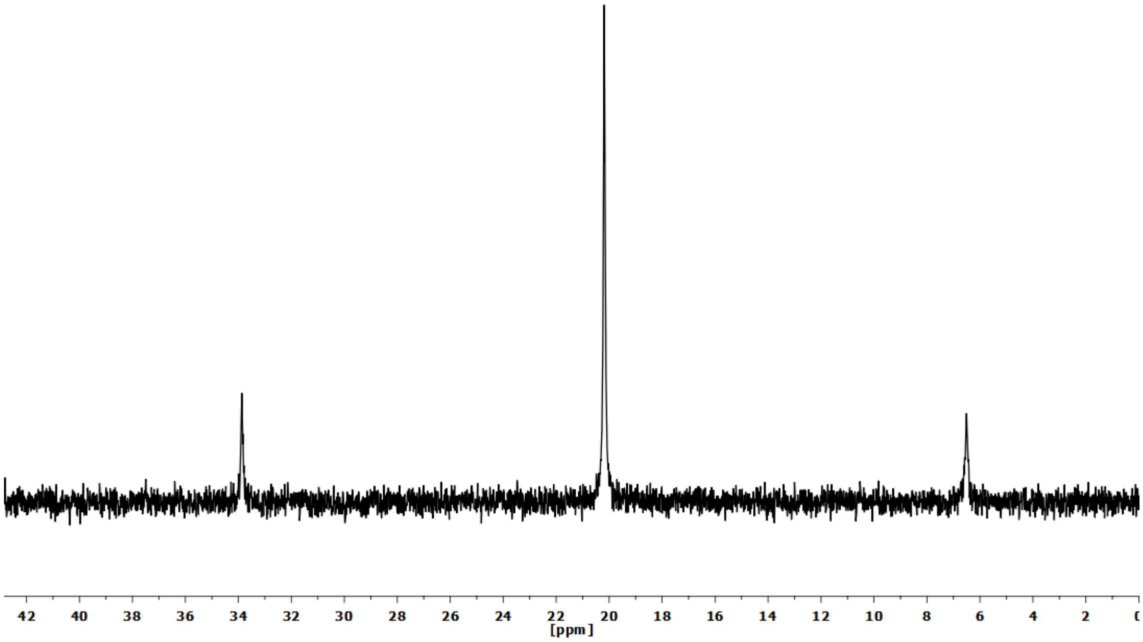

Figure S16. $^{31}P\{^{1}H\}$ NMR spectrum (RT, CD_2Cl_2) of complex **2**.

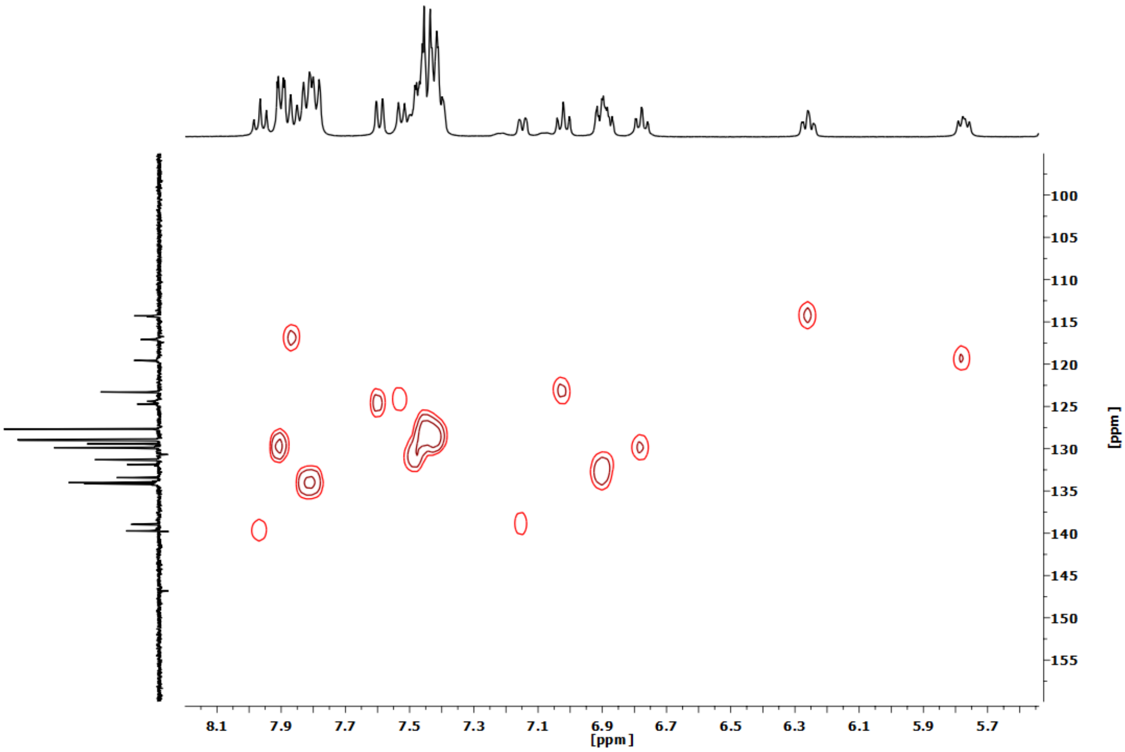

Figure S17. ^{1}H-^{13}C HSQC NMR spectrum (RT, CD_2Cl_2) of complex **2**.

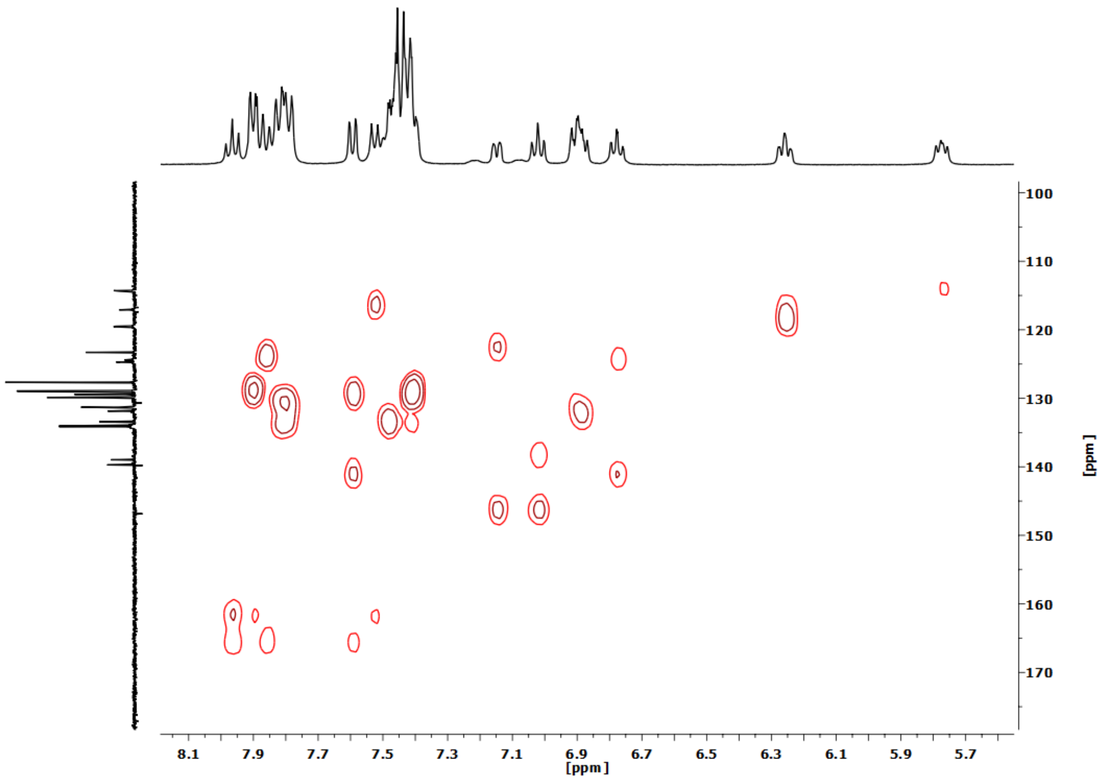

Figure S18. ^{1}H-^{13}C HMBC NMR spectrum (RT, CD_2Cl_2) of complex **2**.

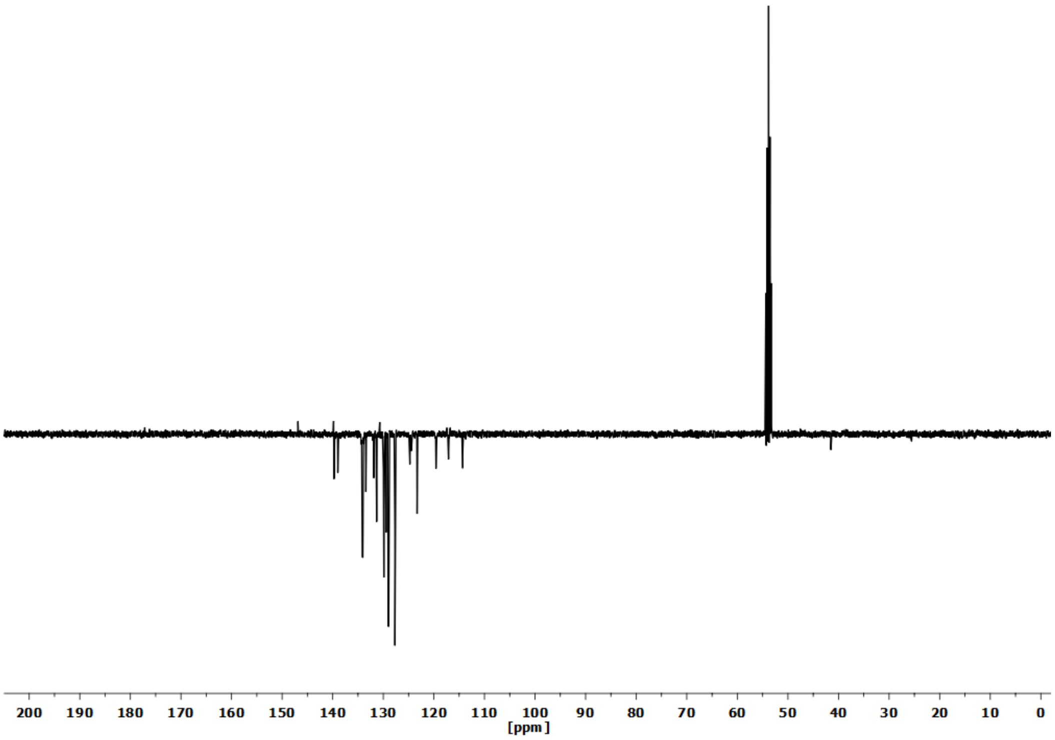

Figure S19. APT $^{13}C\{^{1}H\}$ NMR spectrum (RT, CD_2Cl_2) of complex **2**.

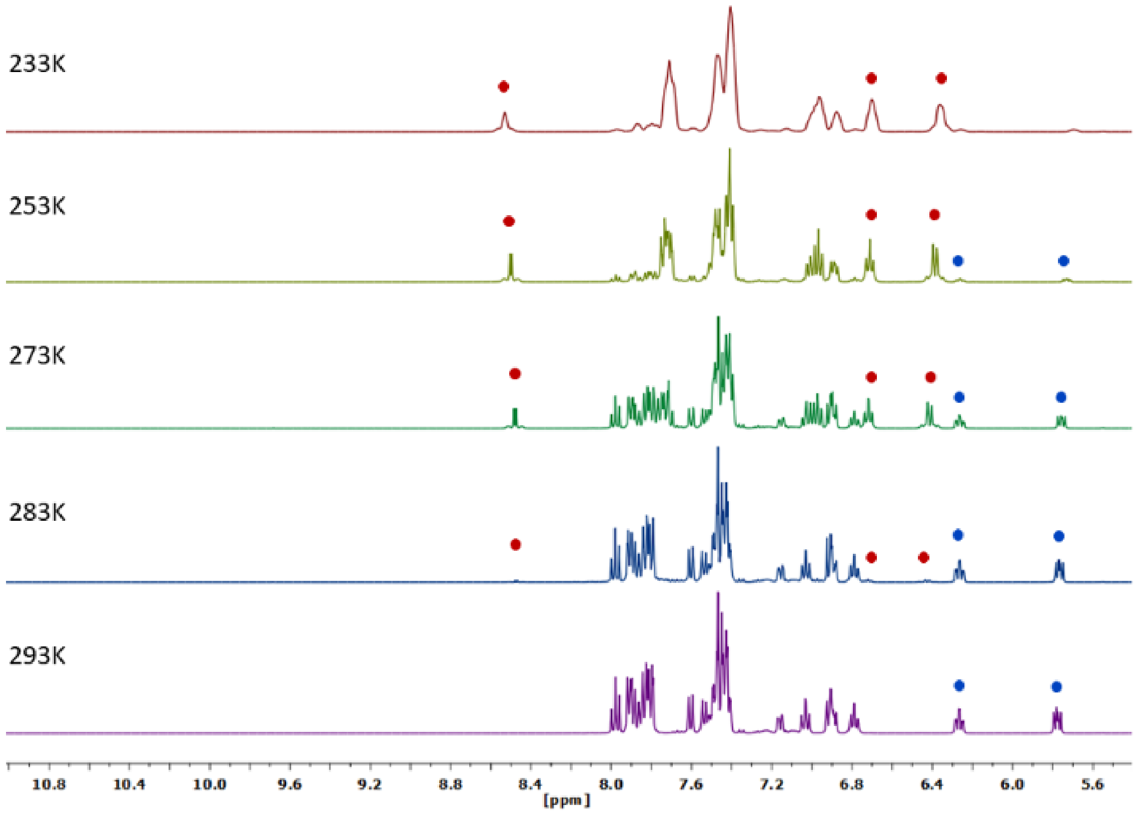

Figure S20. VT ^{1}H NMR spectrum (CD_2Cl_2) of reaction of complex **2**. Time lapse of measurements: 90 minutes.

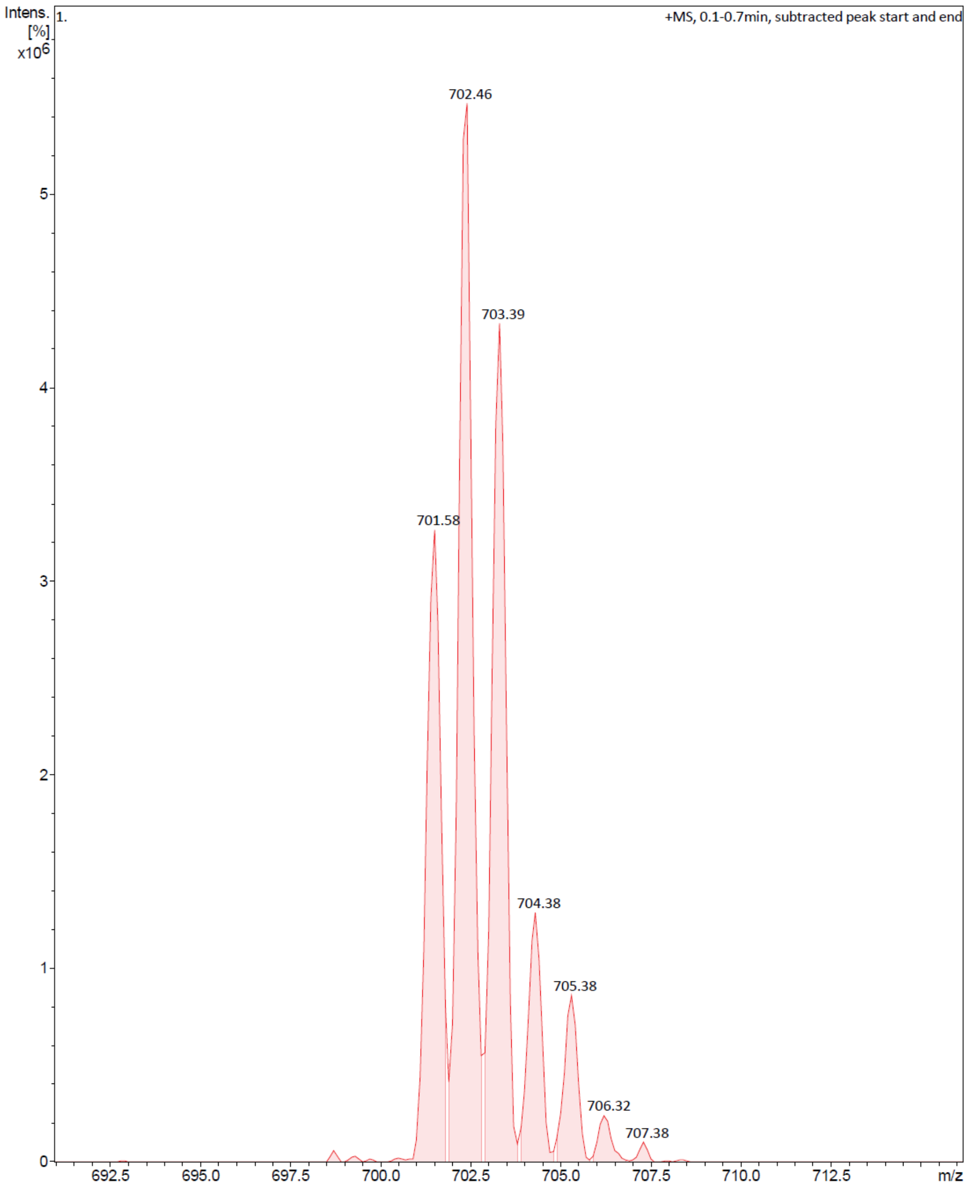

Figure S21. MS (ESI+) molecular peak of complex **2**.

2.3. Spectra of complex [Pt(CNC-H){$PPh_2(C_6H_4$-*o*-COO)}Ag(PPh_3)](ClO_4) (3).

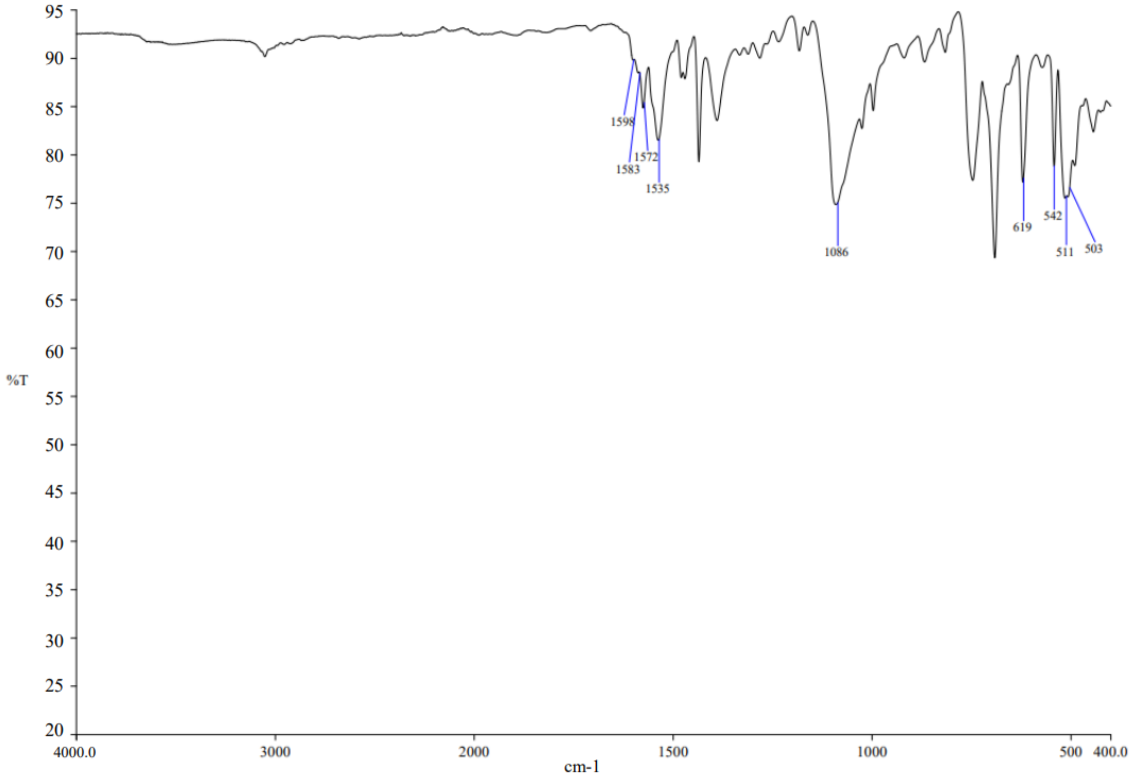

Figure S22. ATR-IR spectrum of complex **3**.

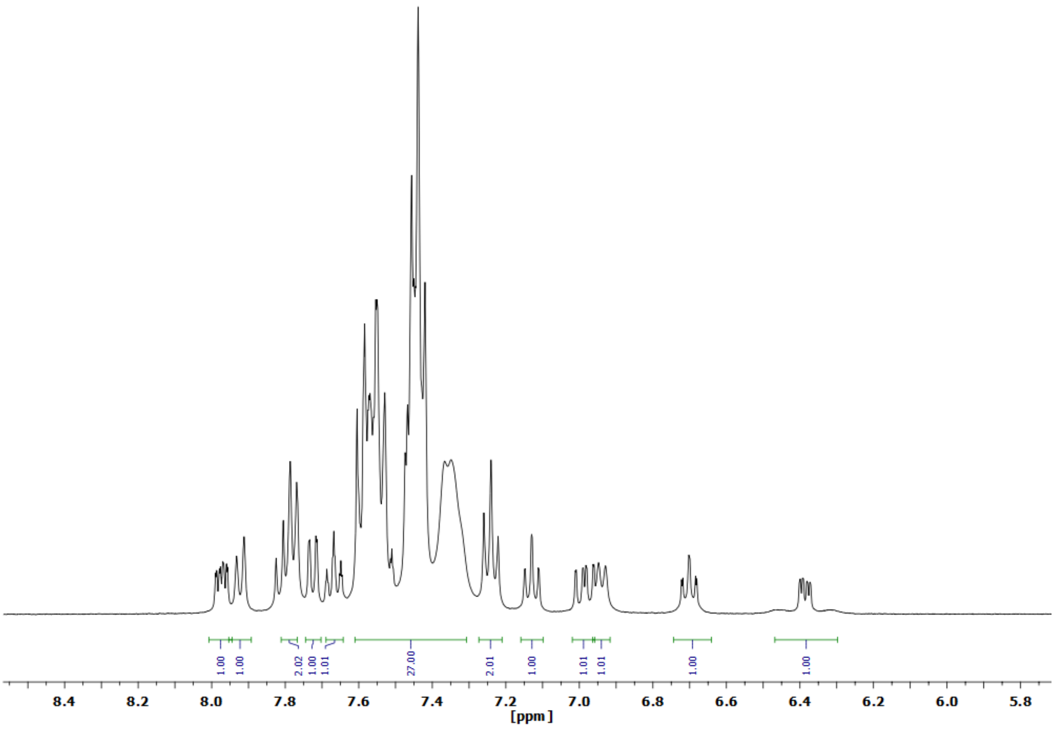

Figure S23. ^{1}H NMR spectrum (RT, CD_2Cl_2) of complex **3**.

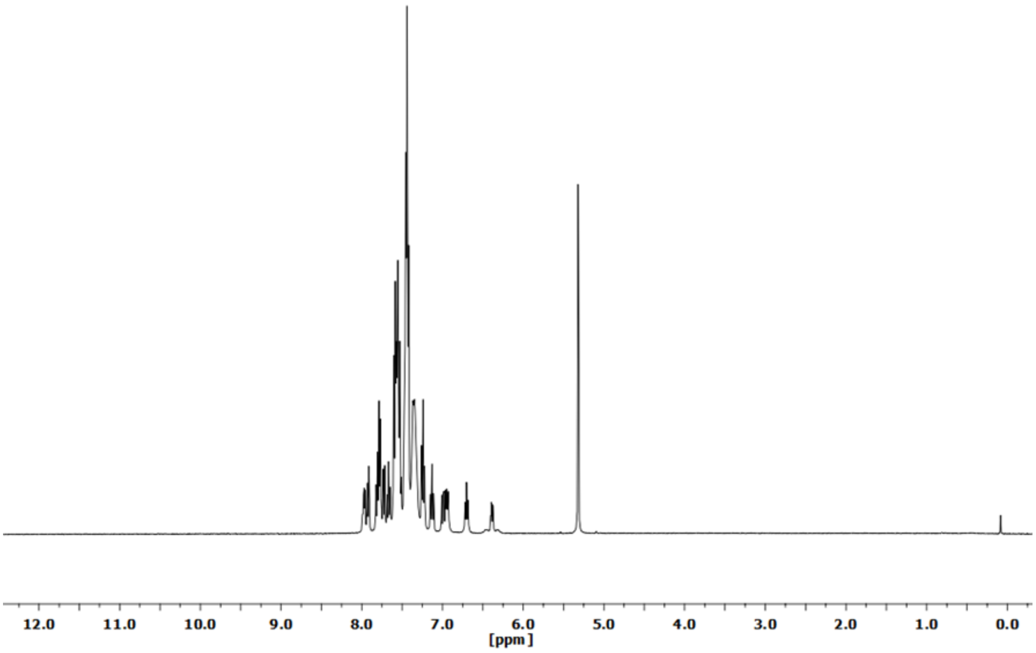

Figure S24. Full ^{1}H NMR spectrum (RT, CD_2Cl_2) of complex **3**.

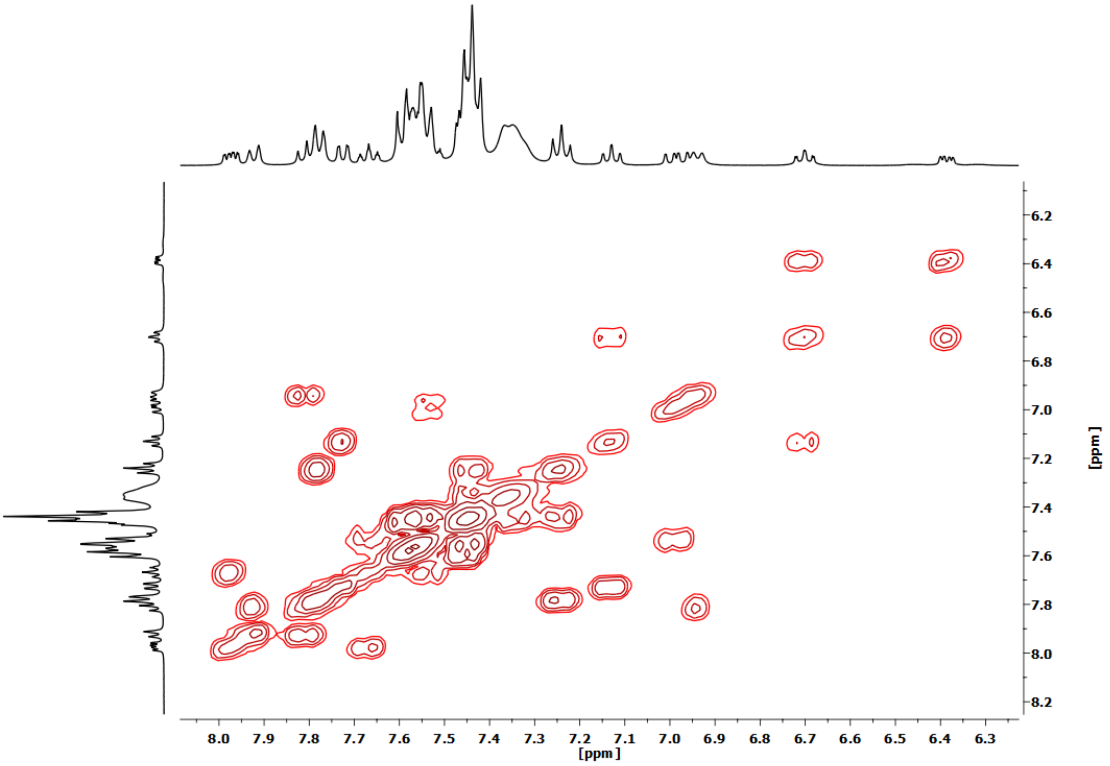

Figure S25. ^{1}H-^{1}H COSY NMR spectrum (RT, CD_2Cl_2) of complex **3**.

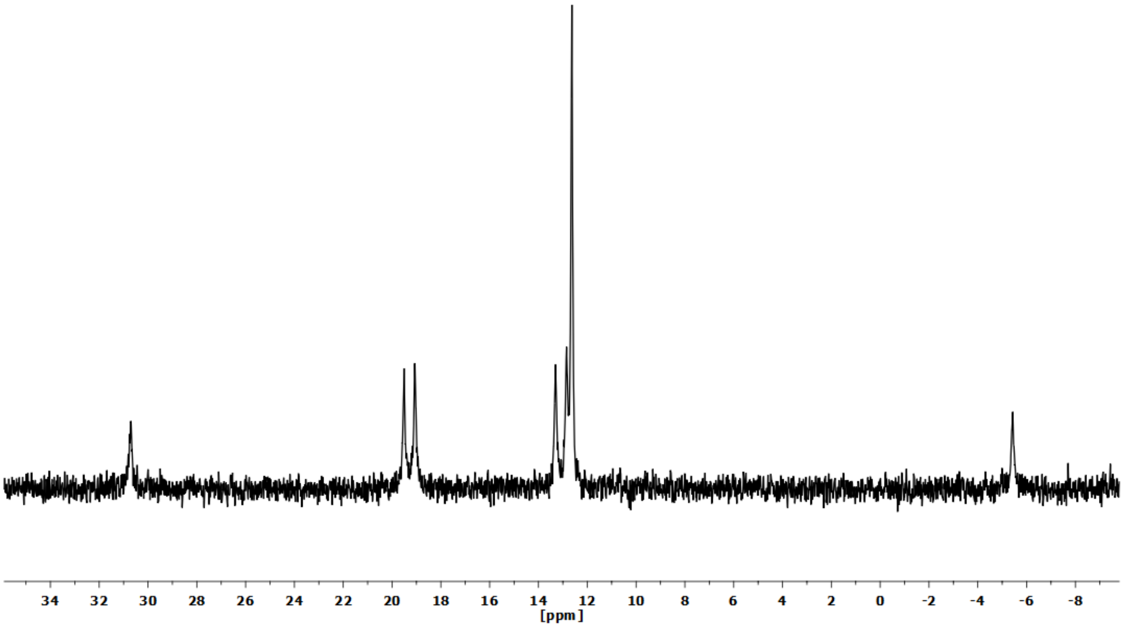

Figure S26. $^{31}P\{^{1}H\}$ NMR spectrum (RT, CD_2Cl_2) of complex **3**.

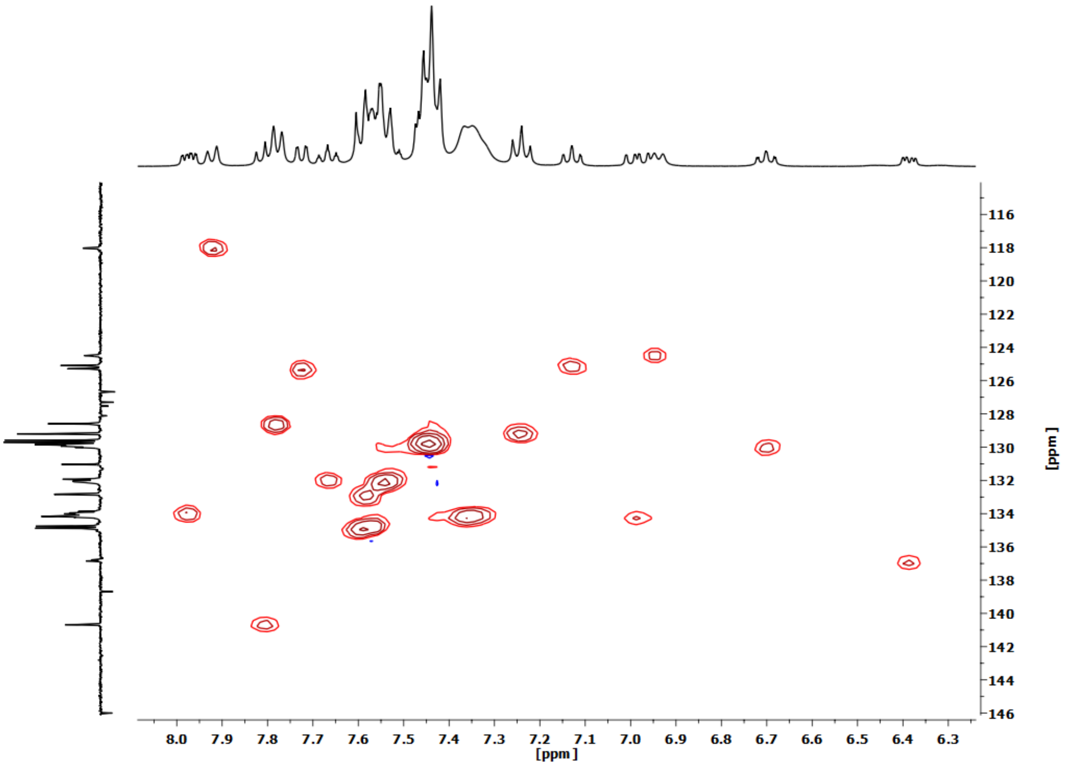

Figure S27. ^{1}H-^{13}C HSQC NMR spectrum (RT, CD_2Cl_2) of complex **3**.

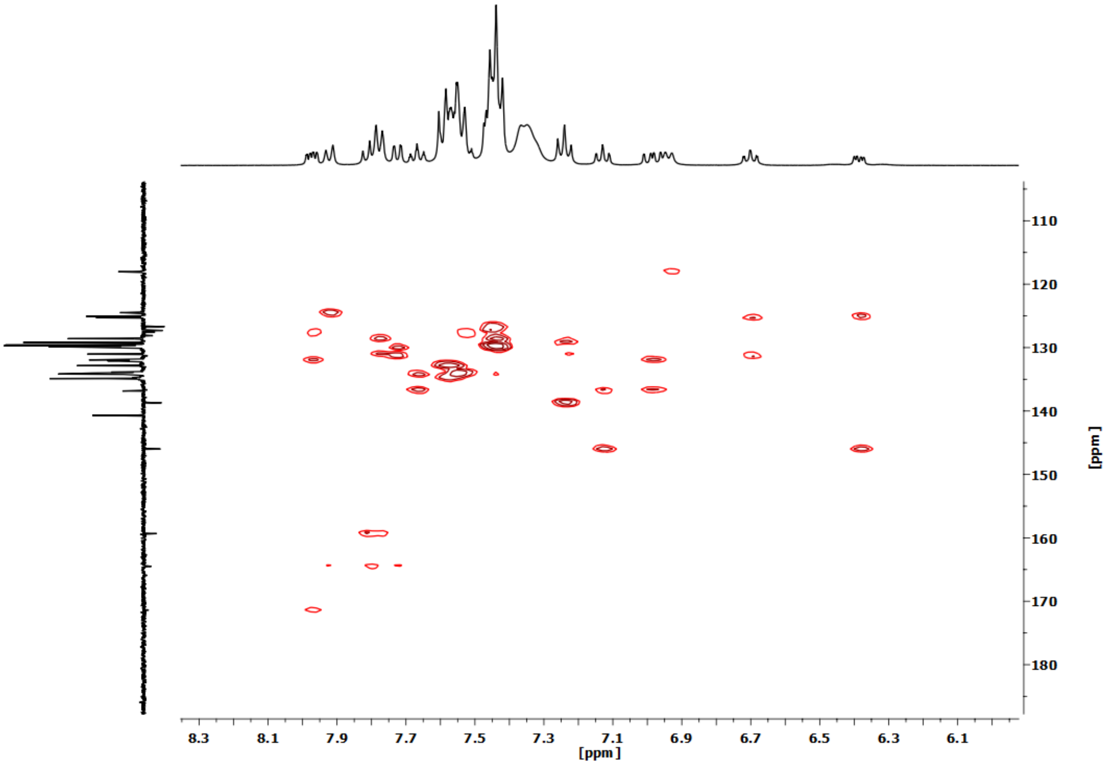

Figure S28. ^{1}H-^{13}C HMBC NMR spectrum (RT, CD_2Cl_2) of complex **3**.

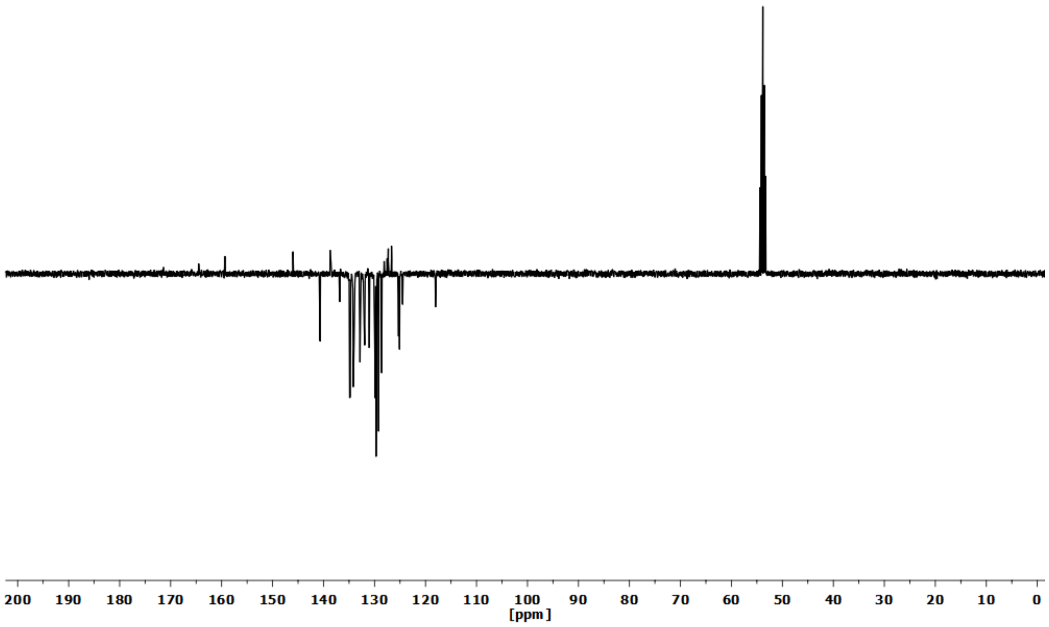

Figure S29. APT ^{13}C{^{1}H} NMR spectrum (RT, CD_2Cl_2) of complex **3**.

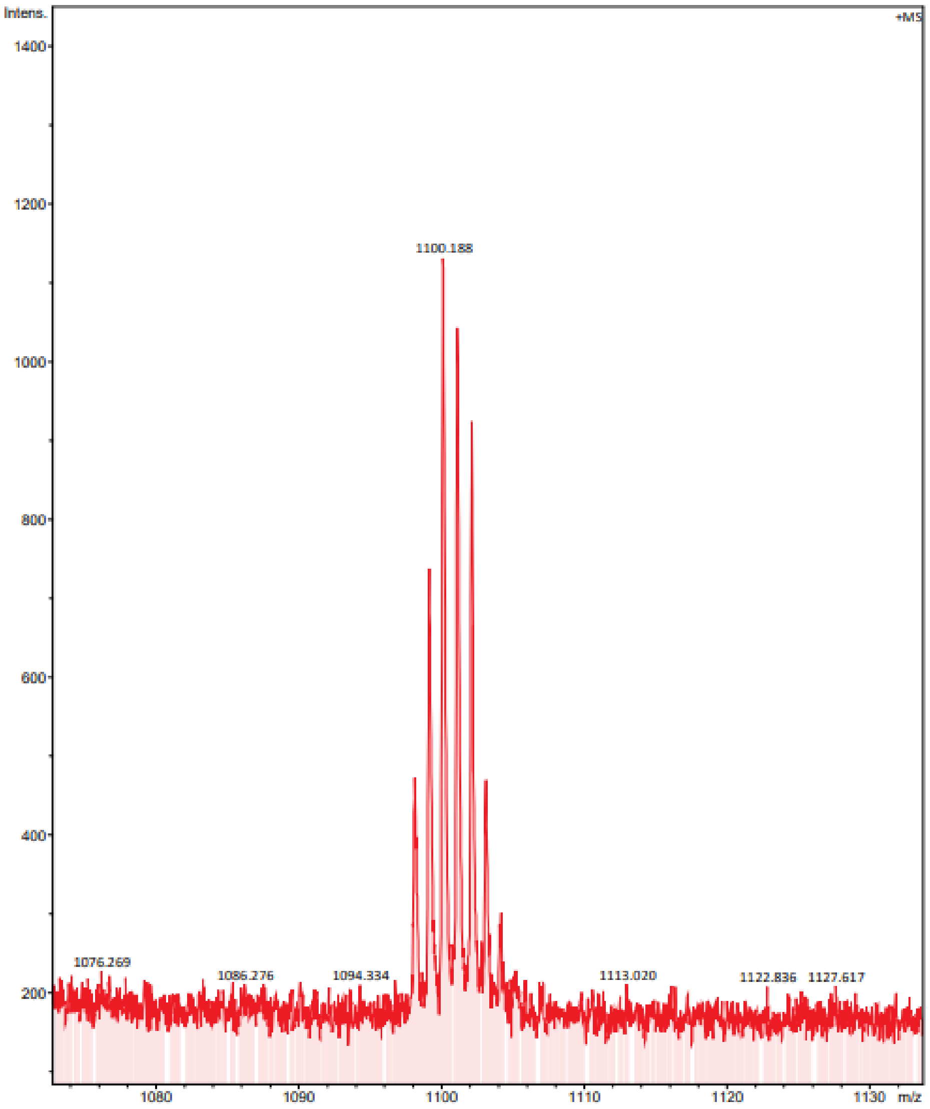

Figure S30. MS (MALDI+ DCTB) molecular peak of complex **3**.

2.4. Spectra of mixture 4*.

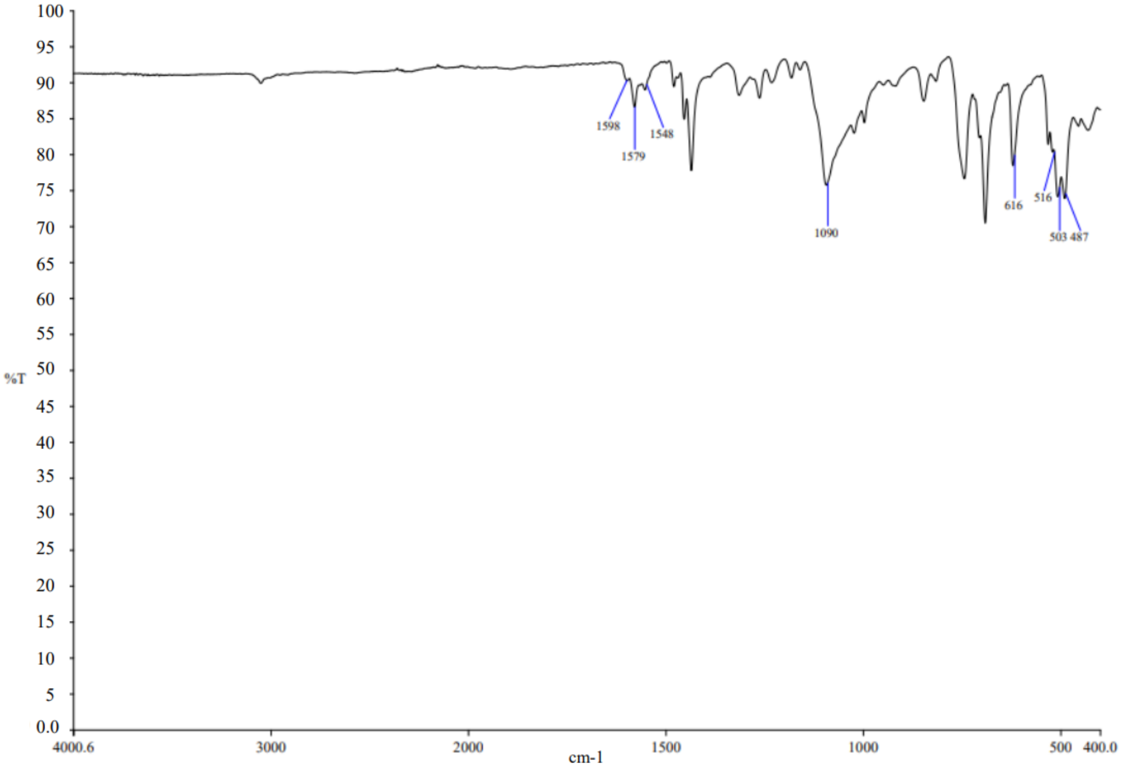

Figure S31. ATR-IR spectrum of **4***.

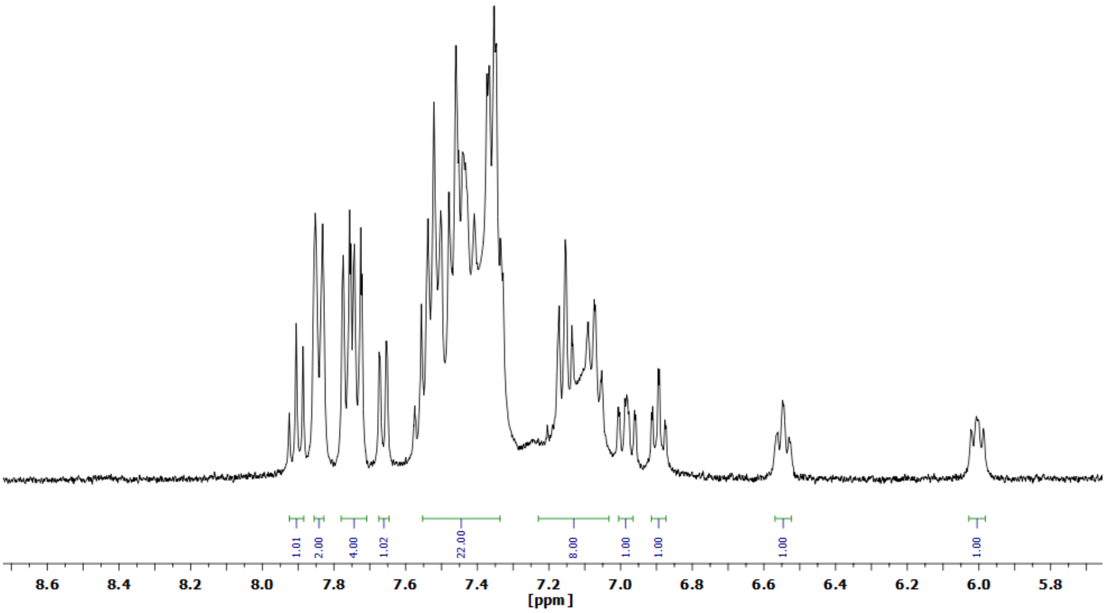

Figure S32. ^{1}H NMR spectrum (RT, CD_2Cl_2) of **4***.

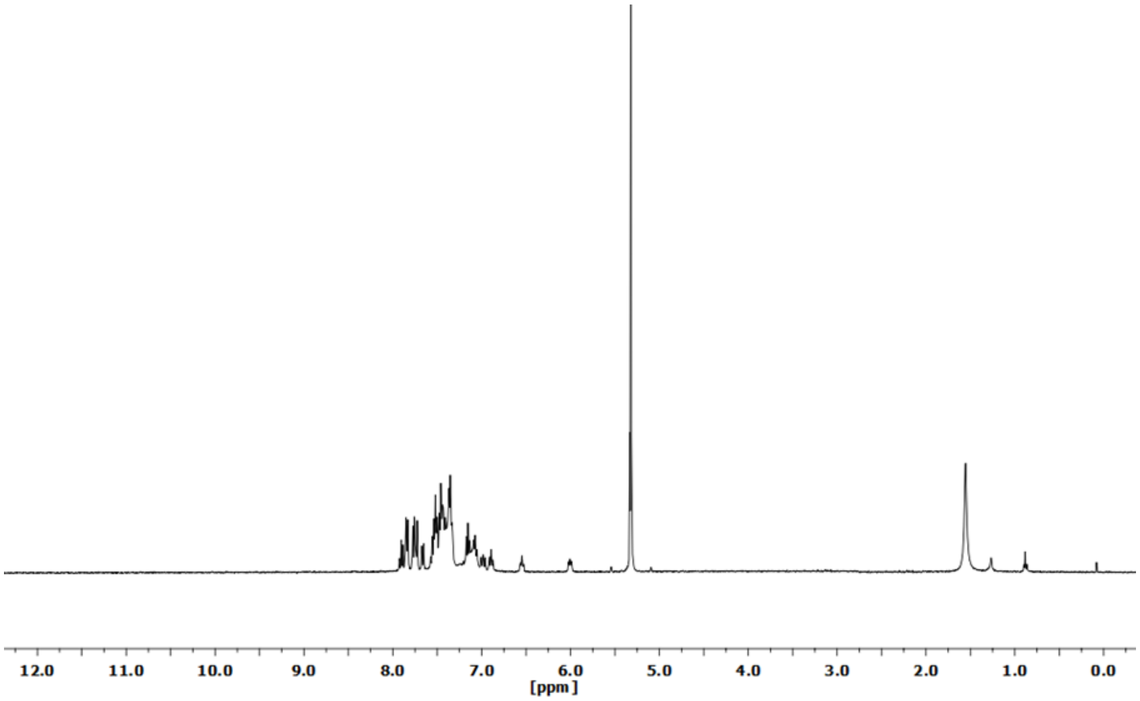

Figure S33. Full ^{1}H NMR spectrum (RT, CD_2Cl_2) of **4***.

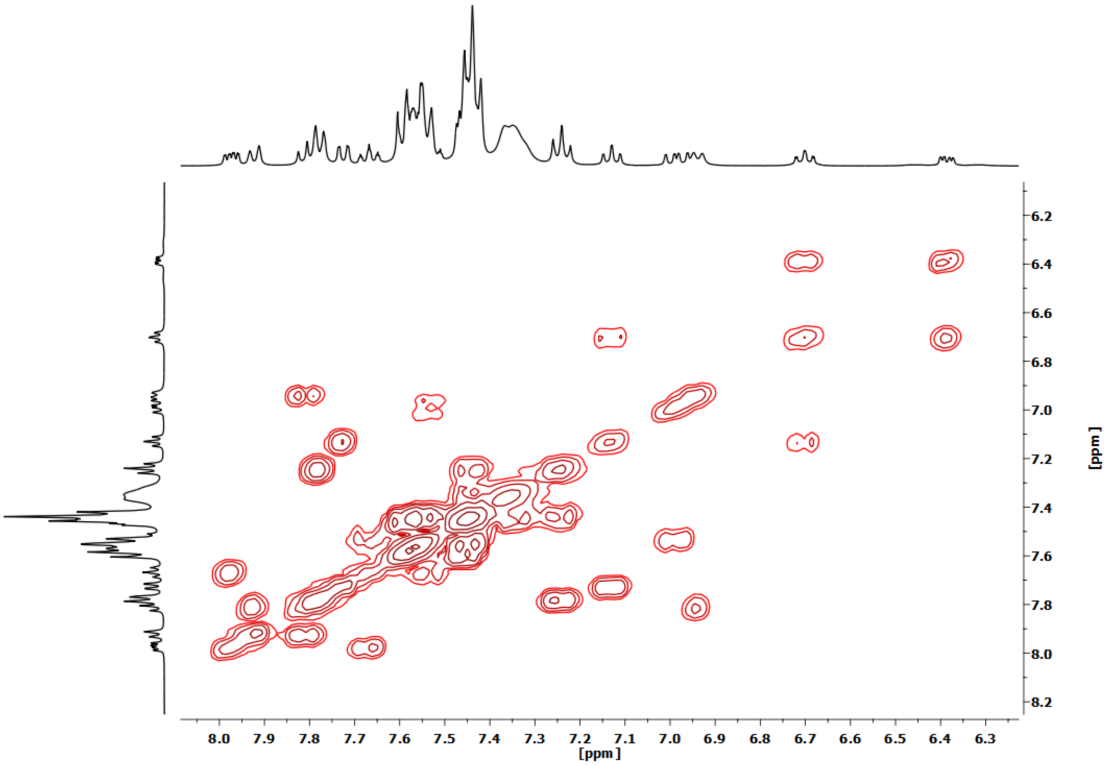

Figure S34. ^{1}H-^{1}H COSY NMR spectrum (RT, CD_2Cl_2) of **4**.

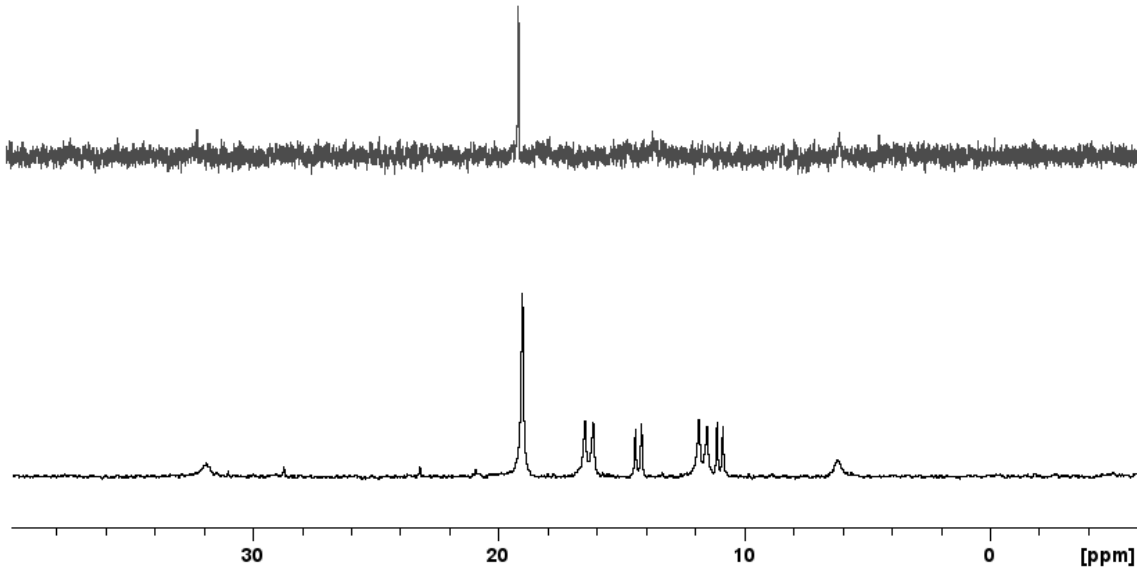

Figure S35. $^{31}P\{^{1}H\}$ NMR spectrum (CD_2Cl_2) of mixture **4***at RT (above) and 173K (below)

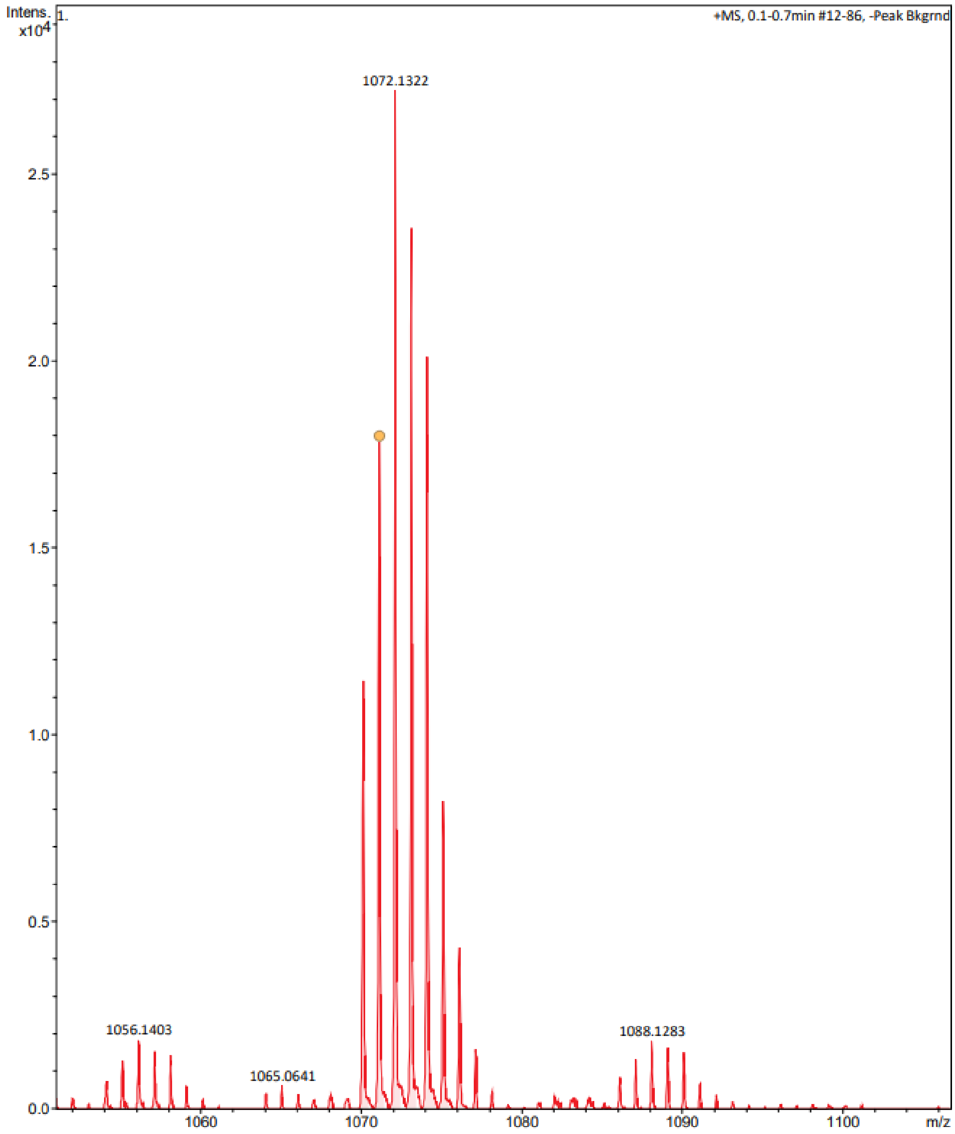

Figure S36. MS (ESI+) molecular peak of complex **4**.

2.5. Spectra of complex [{Pt(CNC-H){PPh$_2$(C$_6$H$_4$-*o*-COO)}}$_2$Ag](ClO$_4$) (5).

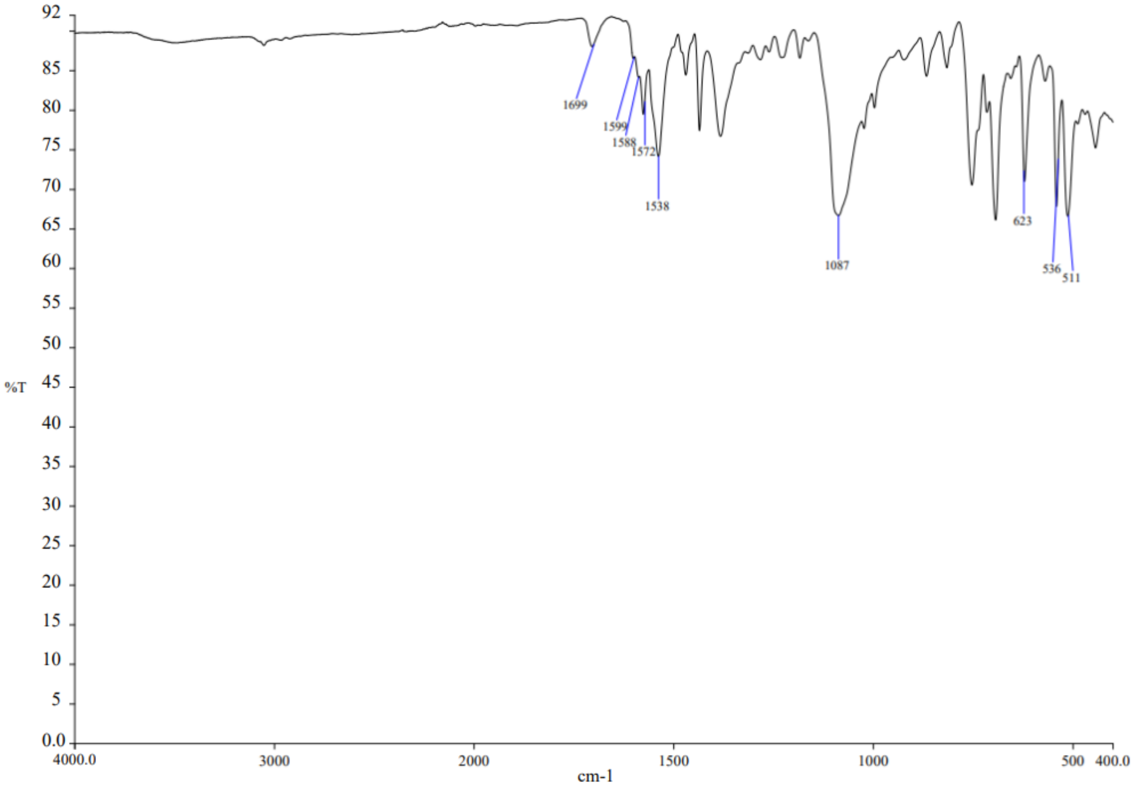

Figure S37. ATR-IR spectrum of complex **5**.

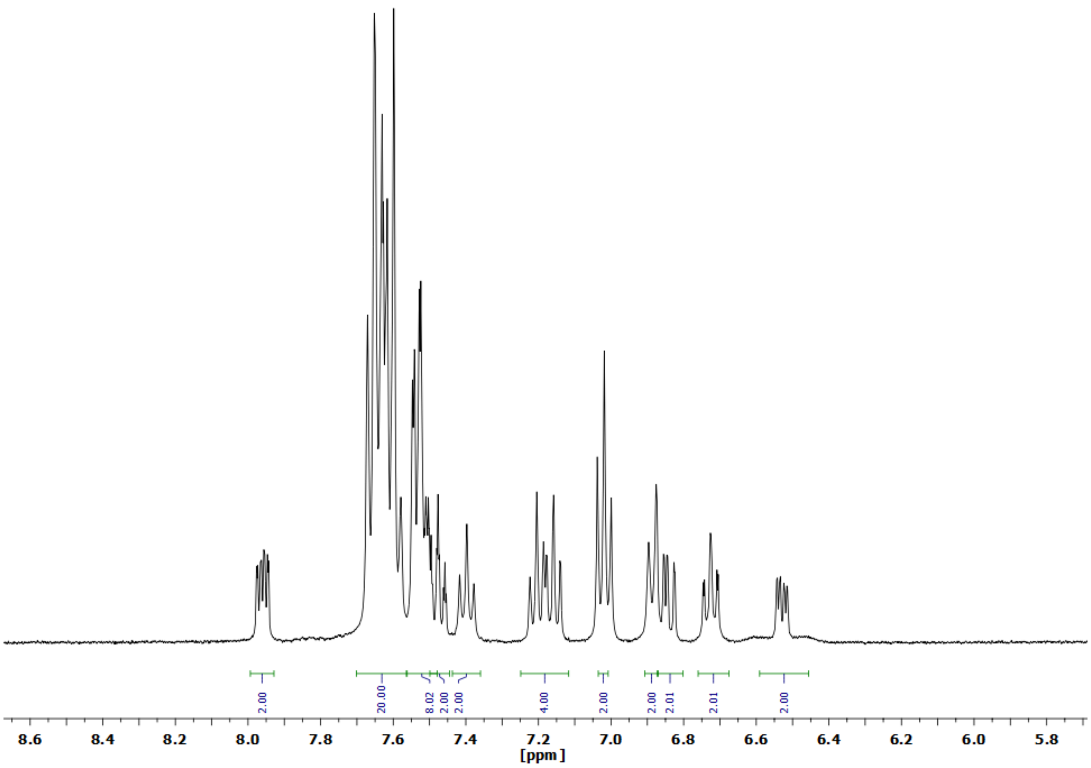

Figure S38. ^{1}H NMR spectrum (RT, CD$_2$Cl$_2$) of complex **5**.

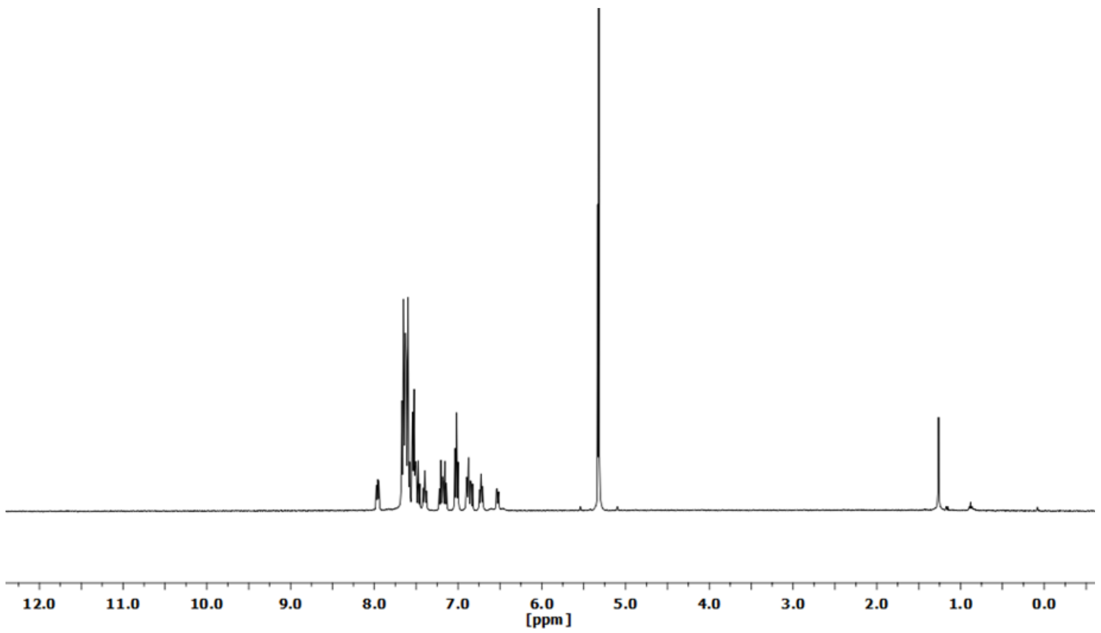

Figure S39. Full ^1H NMR spectrum (RT, CD_2Cl_2) of complex **5**.

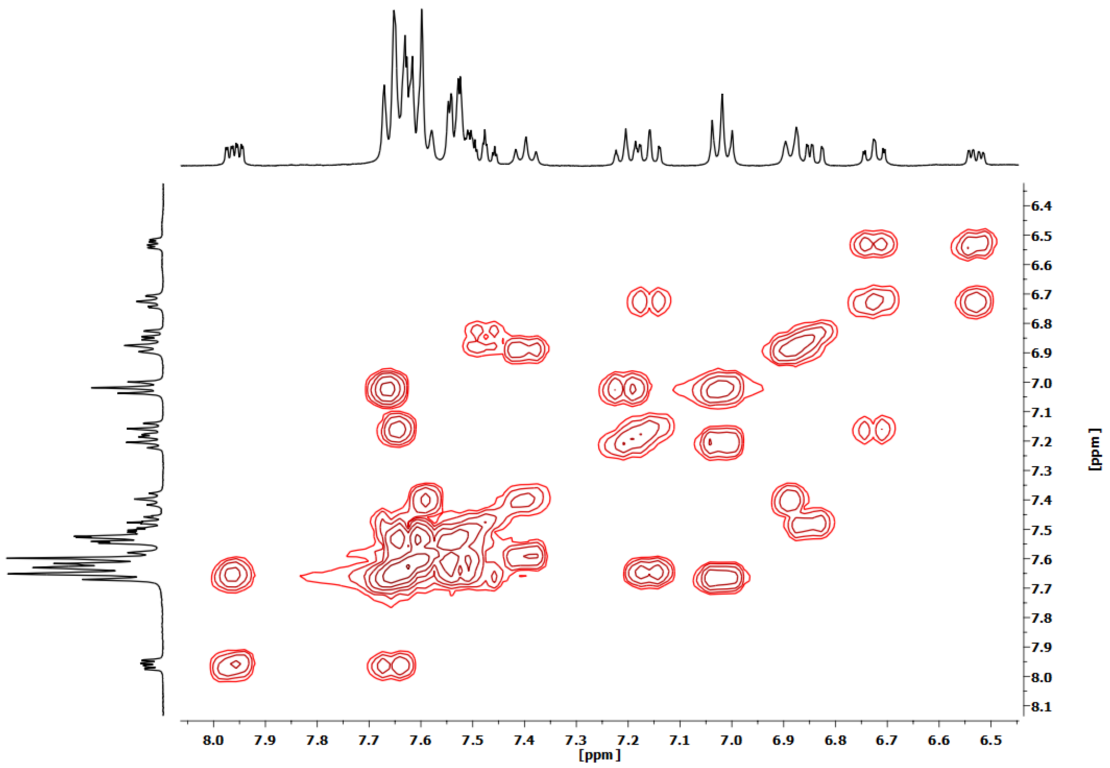

Figure S40. ^1H-^1H COSY NMR spectrum (RT, CD_2Cl_2) of complex **5**.

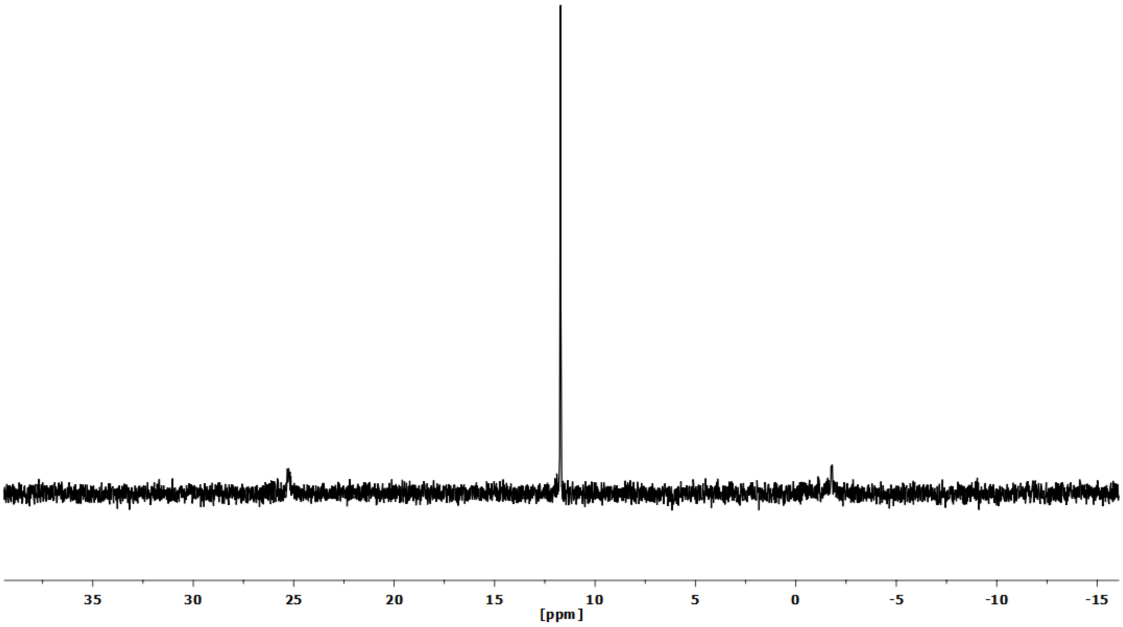

Figure S41. $^{31}P\{^{1}H\}$ NMR spectrum (RT, CD_2Cl_2) of complex **5**.

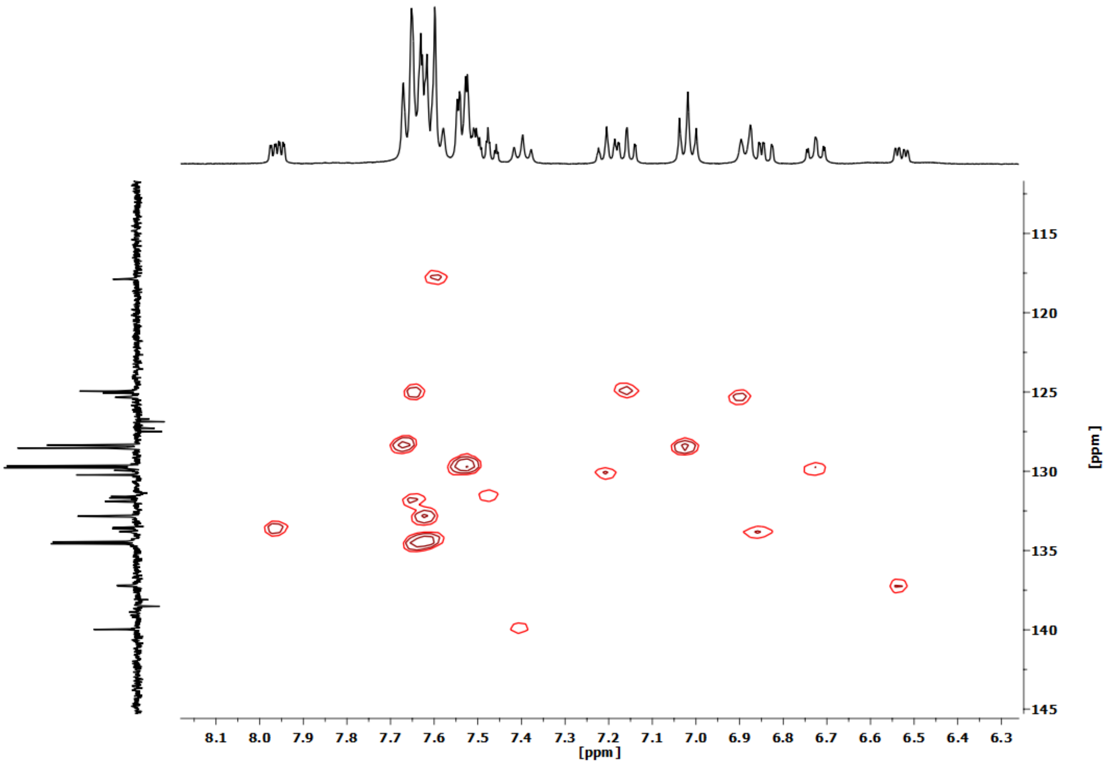

Figure S42. ^{1}H-^{13}C HSQC NMR spectrum (RT, CD_2Cl_2) of complex **5**.

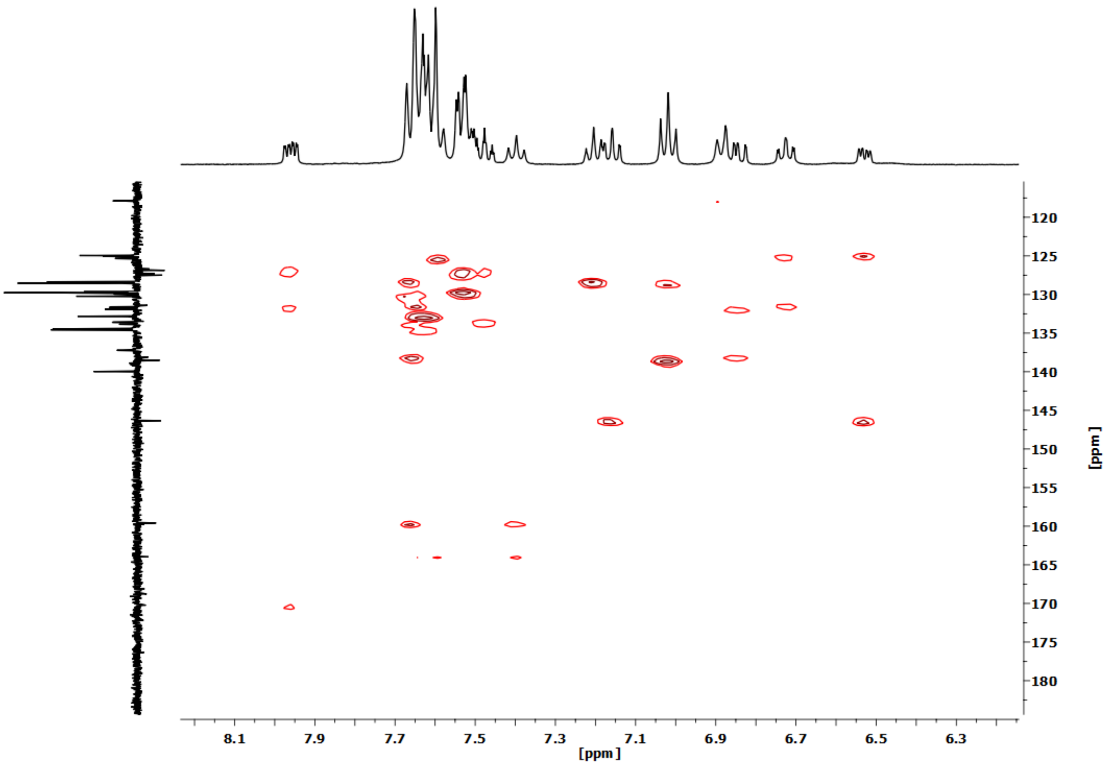

Figure S43. ^{1}H-^{13}C HMBC NMR spectrum (RT, CD_2Cl_2) of complex **5**.

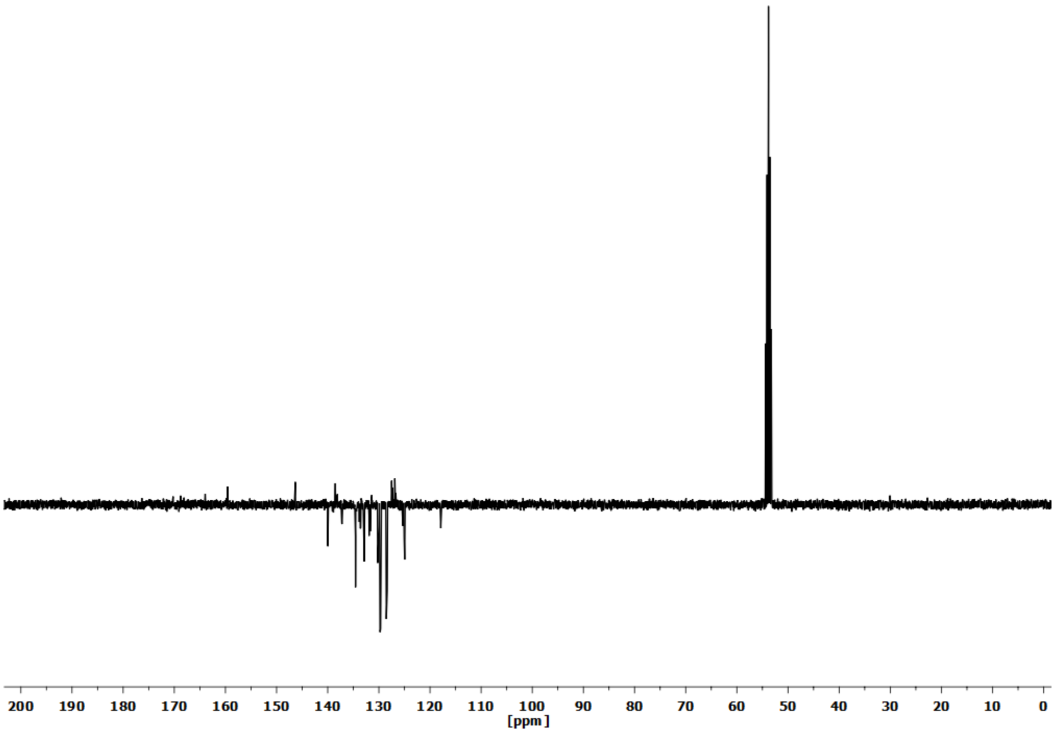

Figure S44. APT $^{13}C\{^{1}H\}$ NMR spectrum (RT, CD_2Cl_2) of complex **5**.

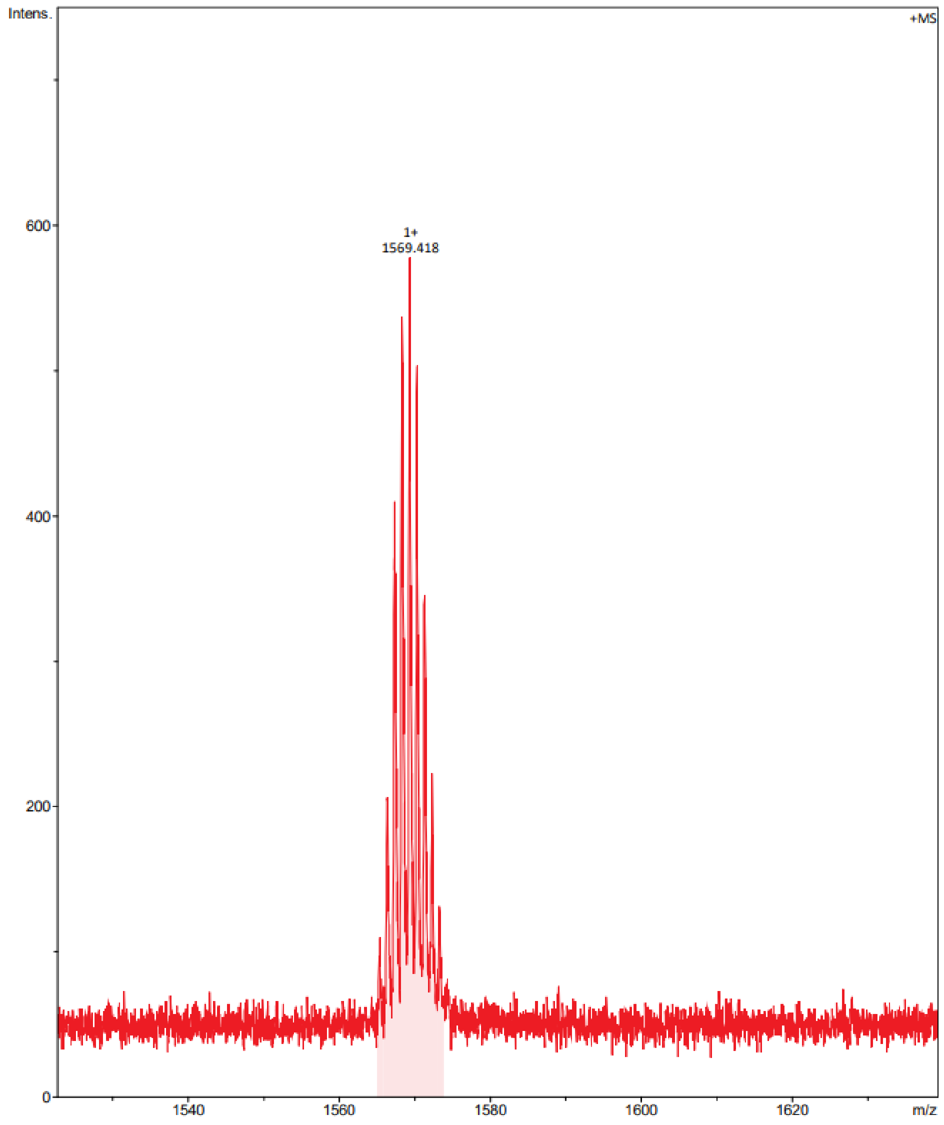

Figure S45. MS (MALDI+ DCTB) molecular peak of complex **5**.

2.6. Spectra of complex [{Pt(CNC-H){PPh$_2$(C$_6$H$_4$-*o*-O)}}$_2$Ag](ClO$_4$) (6)

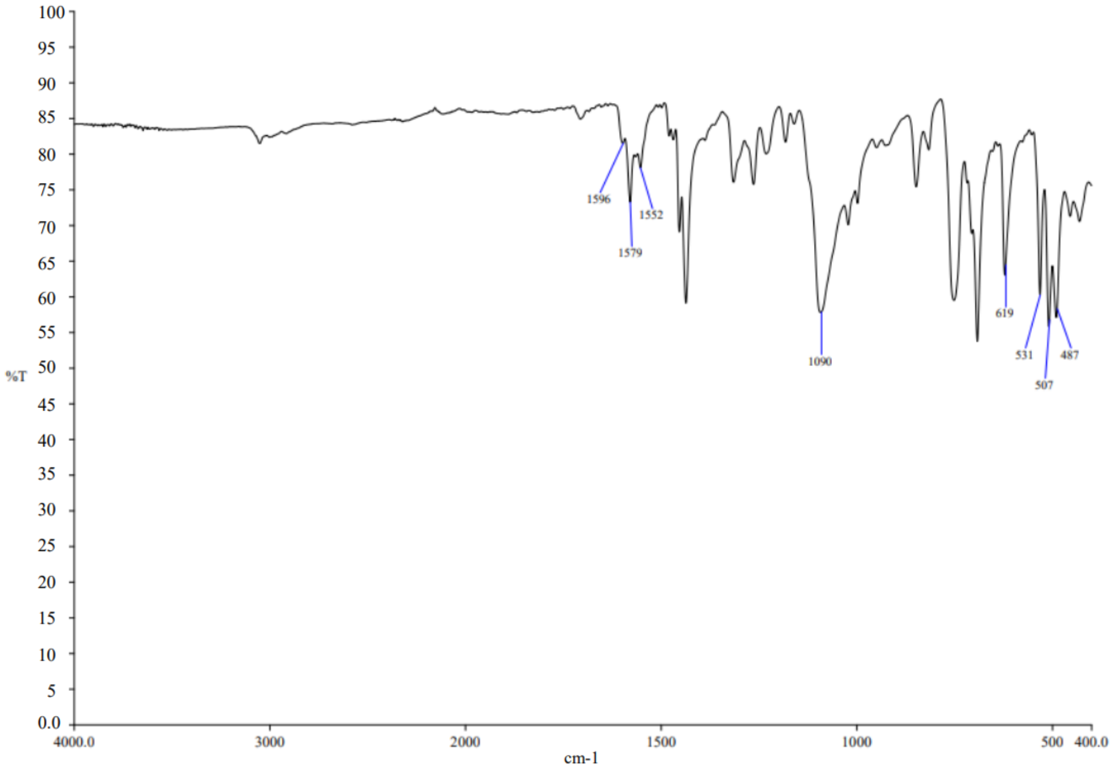

Figure S46. ATR-IR spectrum of complex **6**.

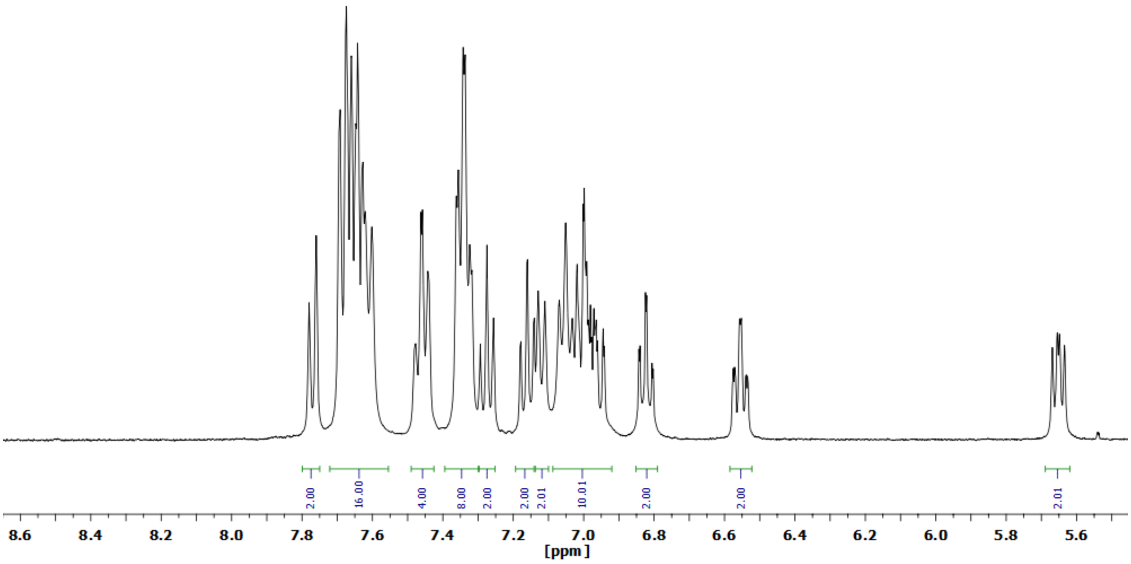

Figure S47. ^{1}H NMR spectrum (RT, CD$_2$Cl$_2$) of complex **6**.

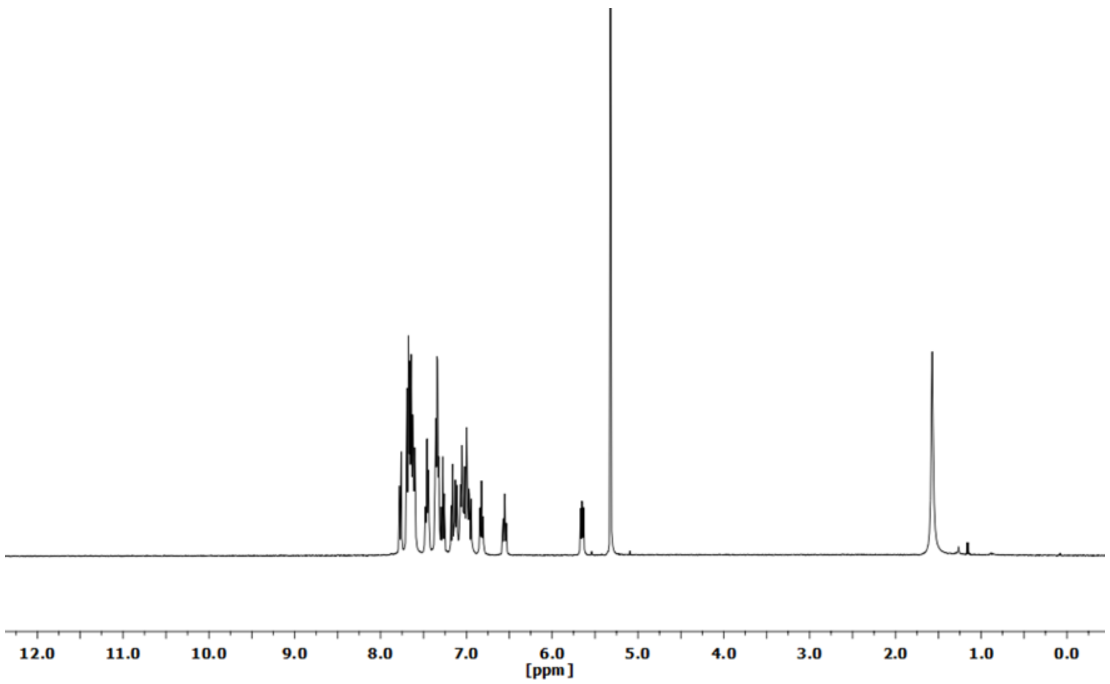

Figure S48. Full ^{1}H NMR spectrum (RT, CD_2Cl_2) of complex **6**.

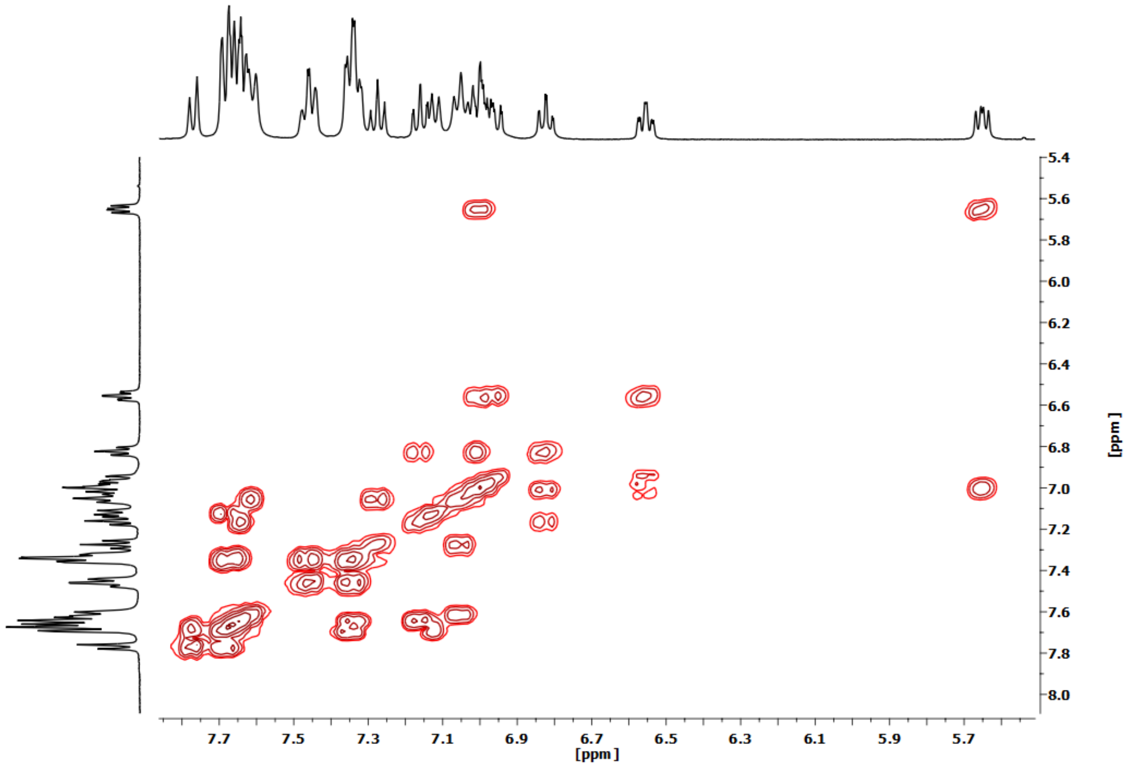

Figure S49. ^{1}H-^{1}H COSY NMR spectrum (RT, CD_2Cl_2) of complex **6.**

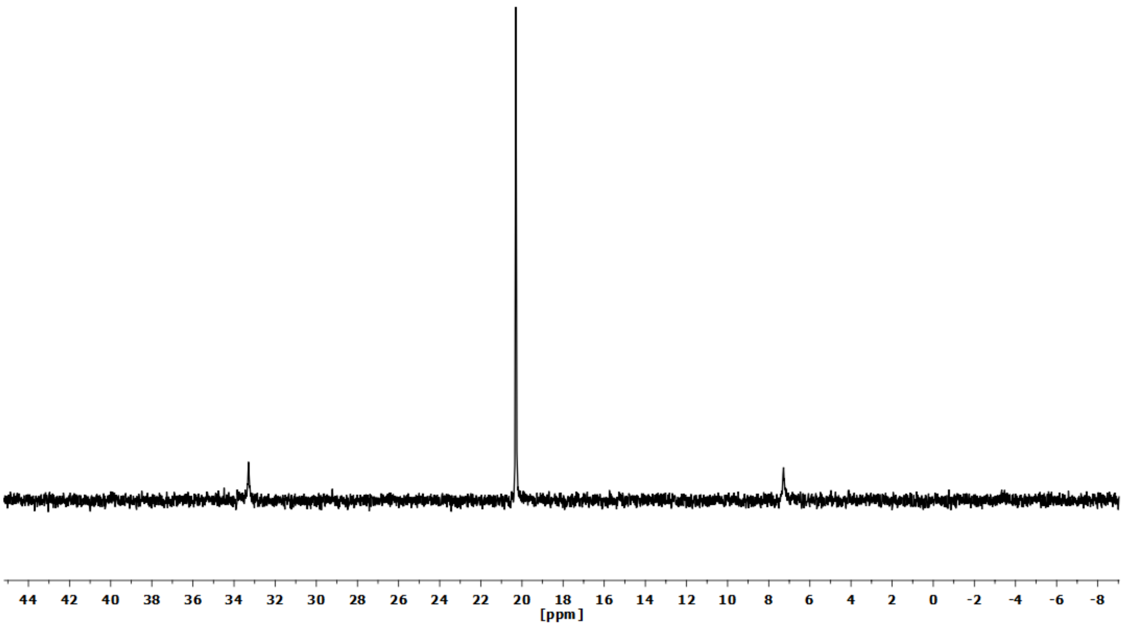

Figure S50. $^{31}P\{^{1}H\}$ NMR spectrum (RT, CD_2Cl_2) of complex **6**.

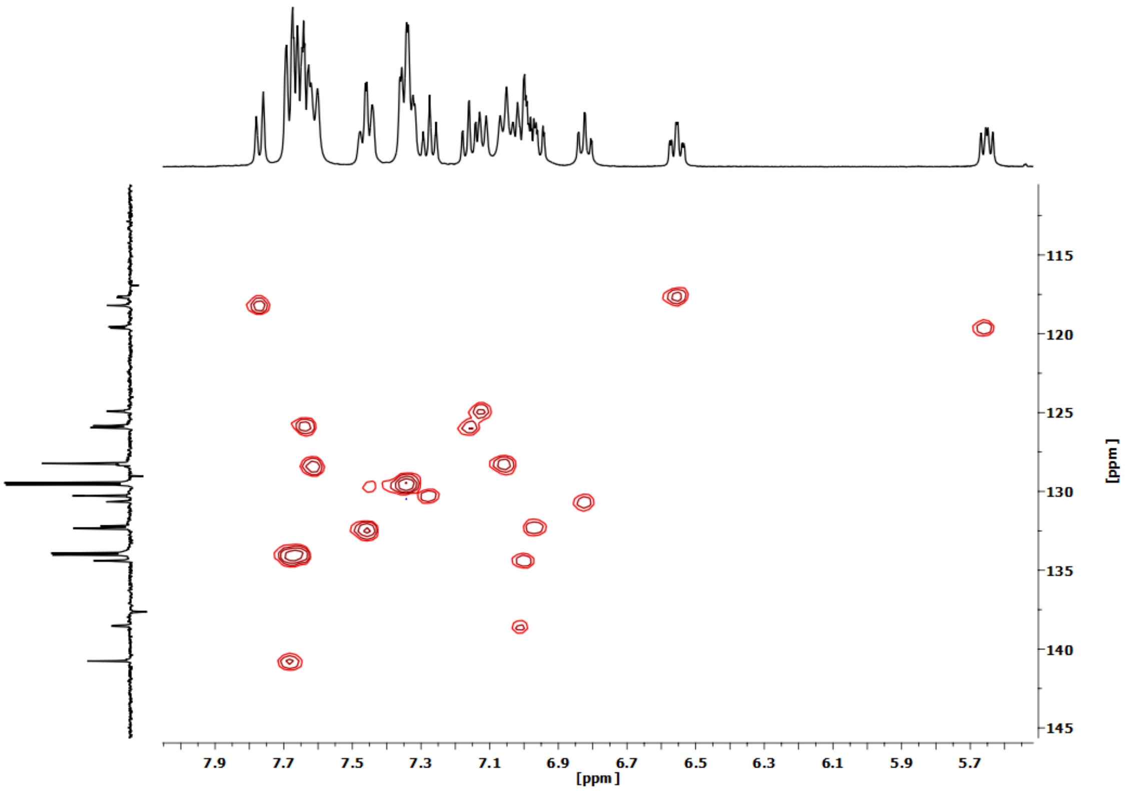

Figure S51. ^{1}H-^{13}C HSQC NMR spectrum (RT, CD_2Cl_2) of complex **6**.

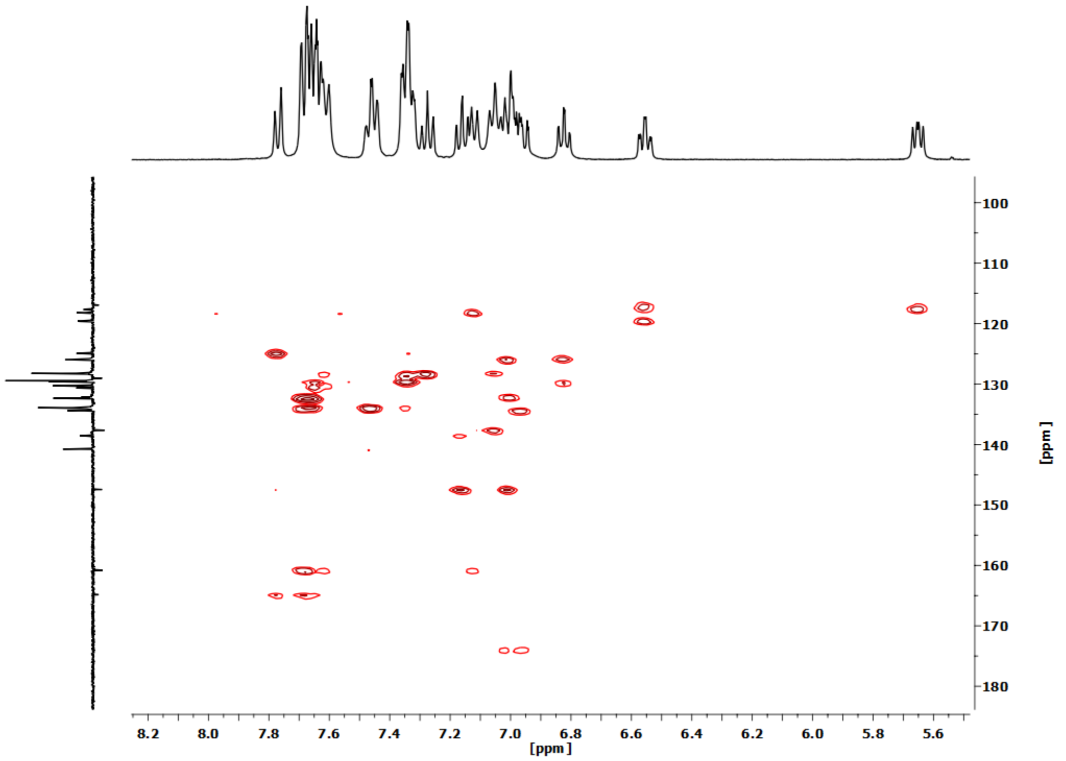

Figure S52. ^{1}H-^{13}C HMBC NMR spectrum (RT, CD_2Cl_2) of complex **6**.

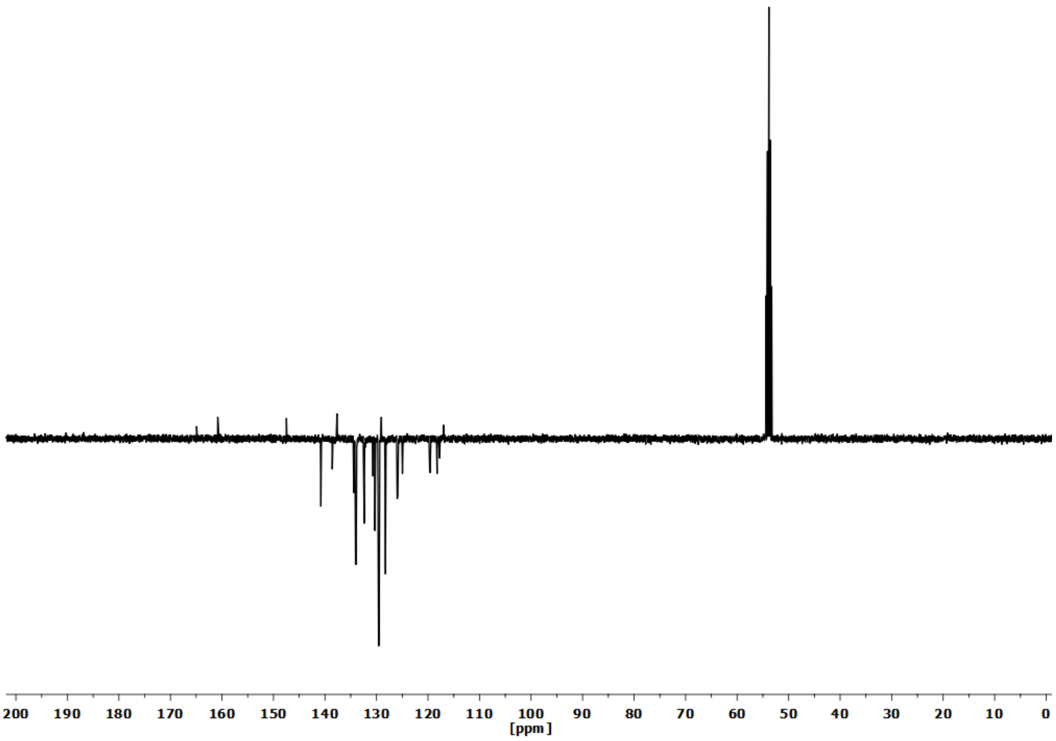

Figure S53. APT $^{13}C\{^{1}H\}$ NMR spectrum (RT, CD_2Cl_2) of complex **6**.

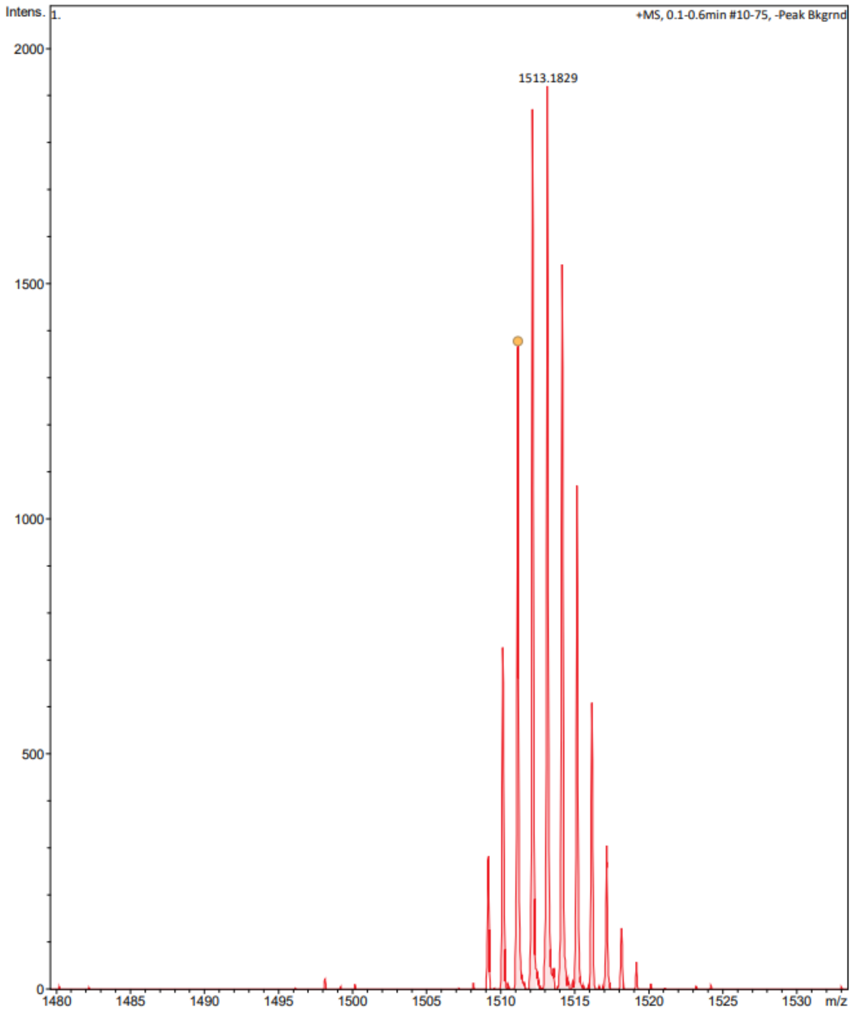

Figure S54. MS (MALDI+ DCTB) molecular peak of complex **6**.

2.7. Spectra of complex [Pt(CNC-H){PPh2(C6H4-*o*-COO)}Au(PPh3)](ClO4) (7)

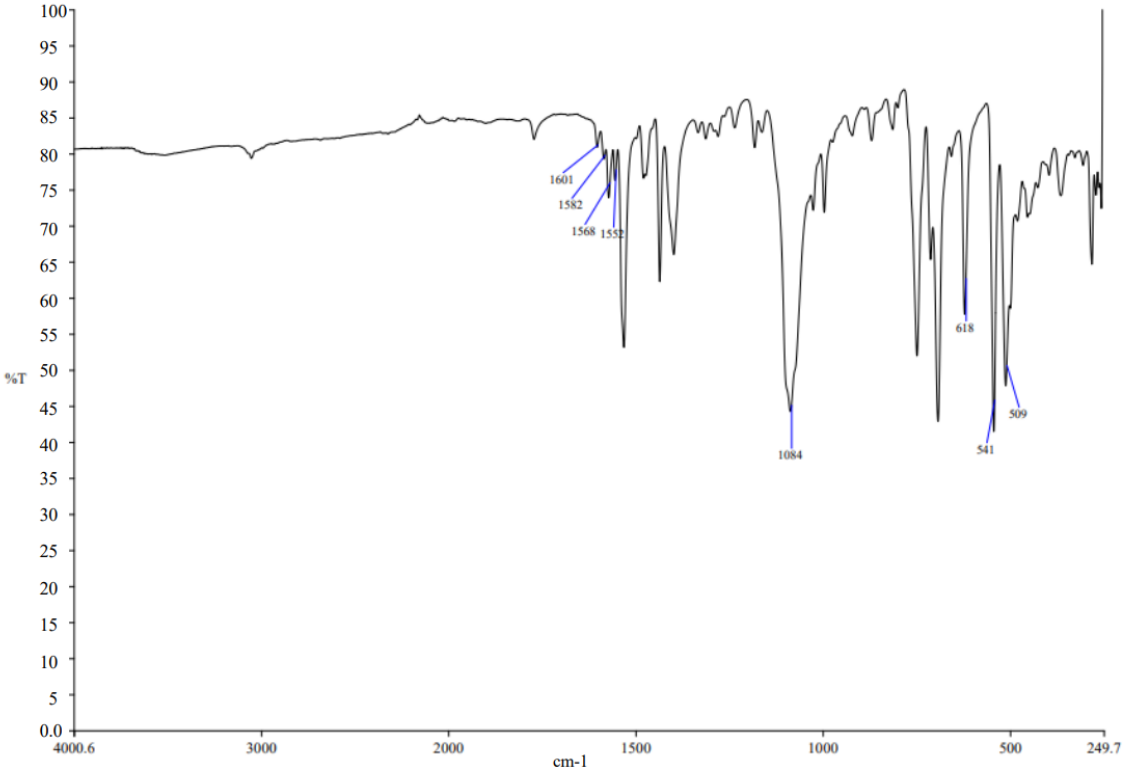

Figure S55. ATR-IR spectrum of complex **7**.

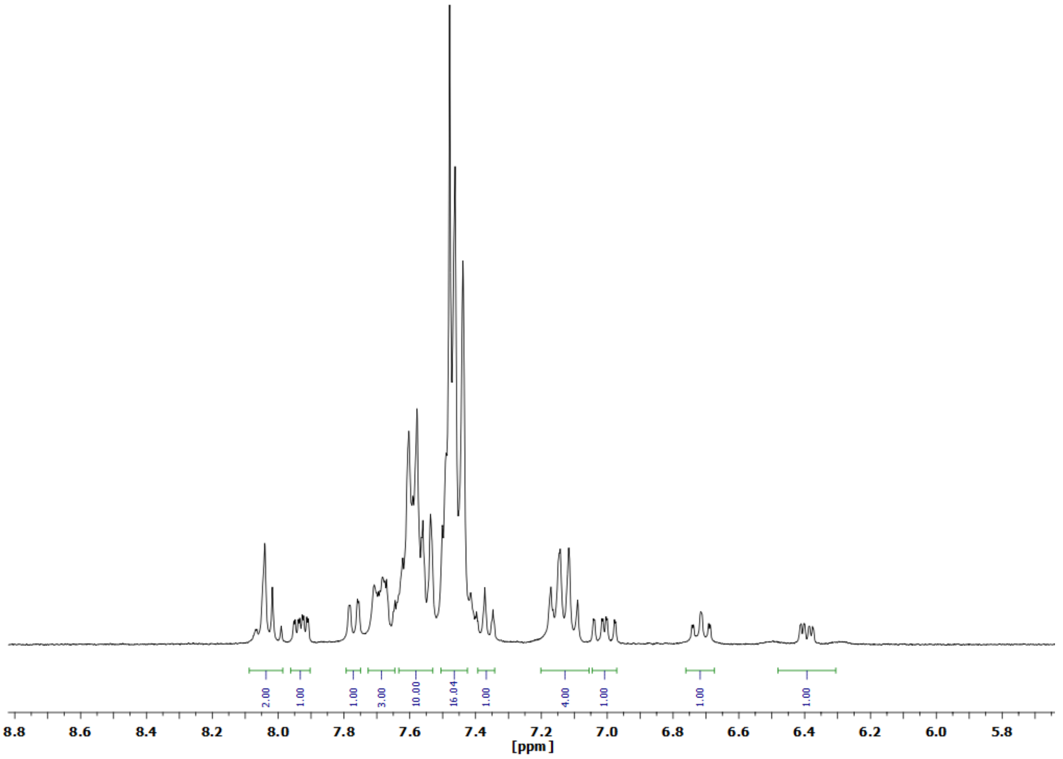

Figure S56. ^{1}H NMR spectrum (RT, CD_2Cl_2) of complex **7**.

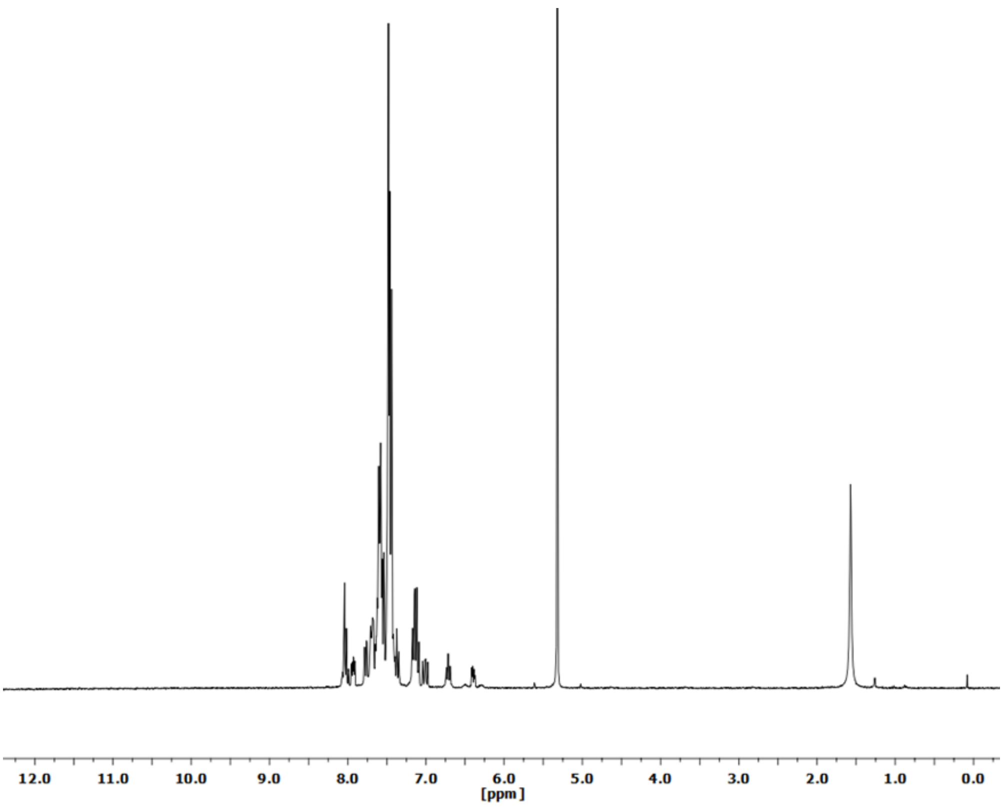

Figure S57. Full ^1H NMR spectrum (RT, CD_2Cl_2) of complex **7**.

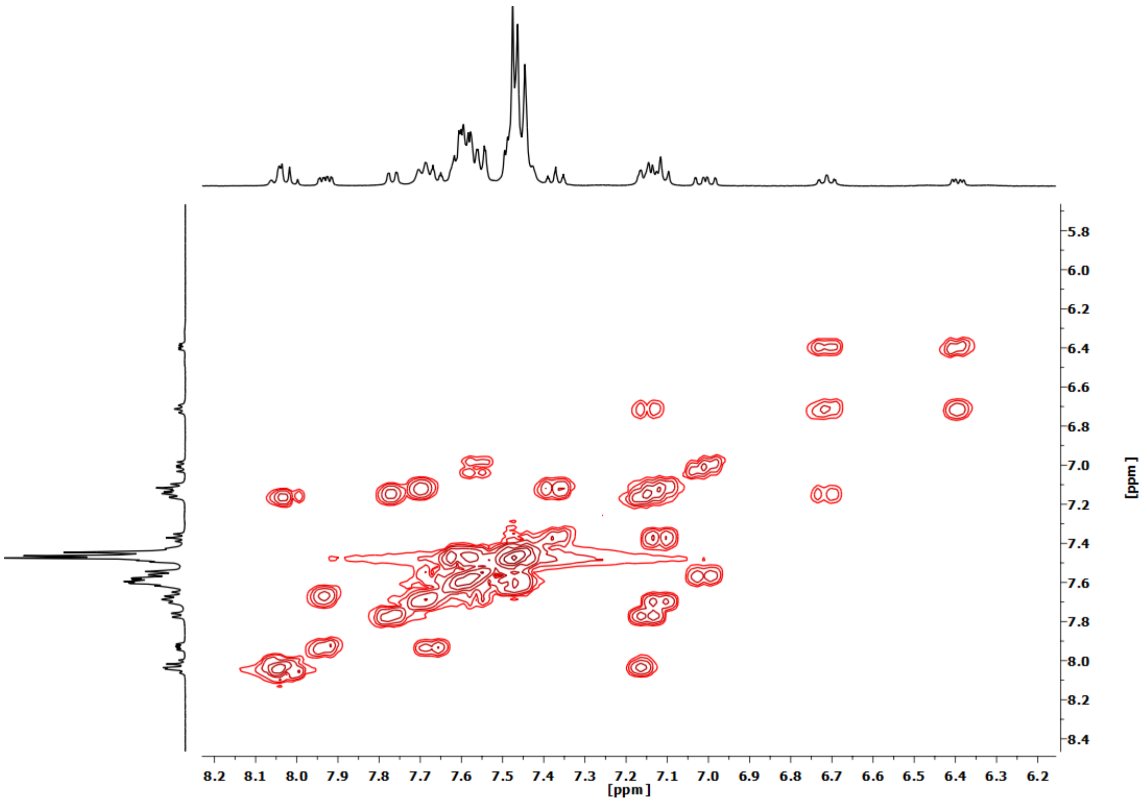

Figure S58. ^1H-^1H COSY NMR spectrum (RT, CD_2Cl_2) of complex **7.**

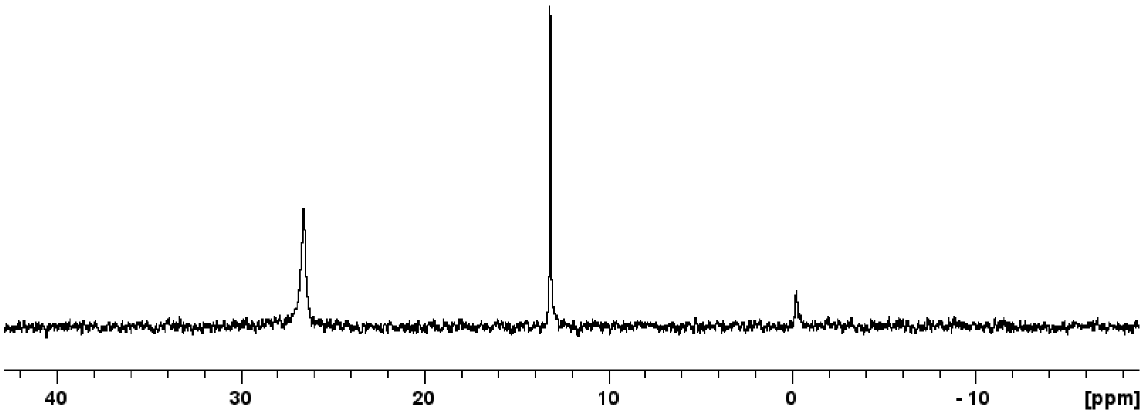

Figure S59. $^{31}P\{^{1}H\}$ NMR spectrum (RT, CD_2Cl_2) of complex **7**.

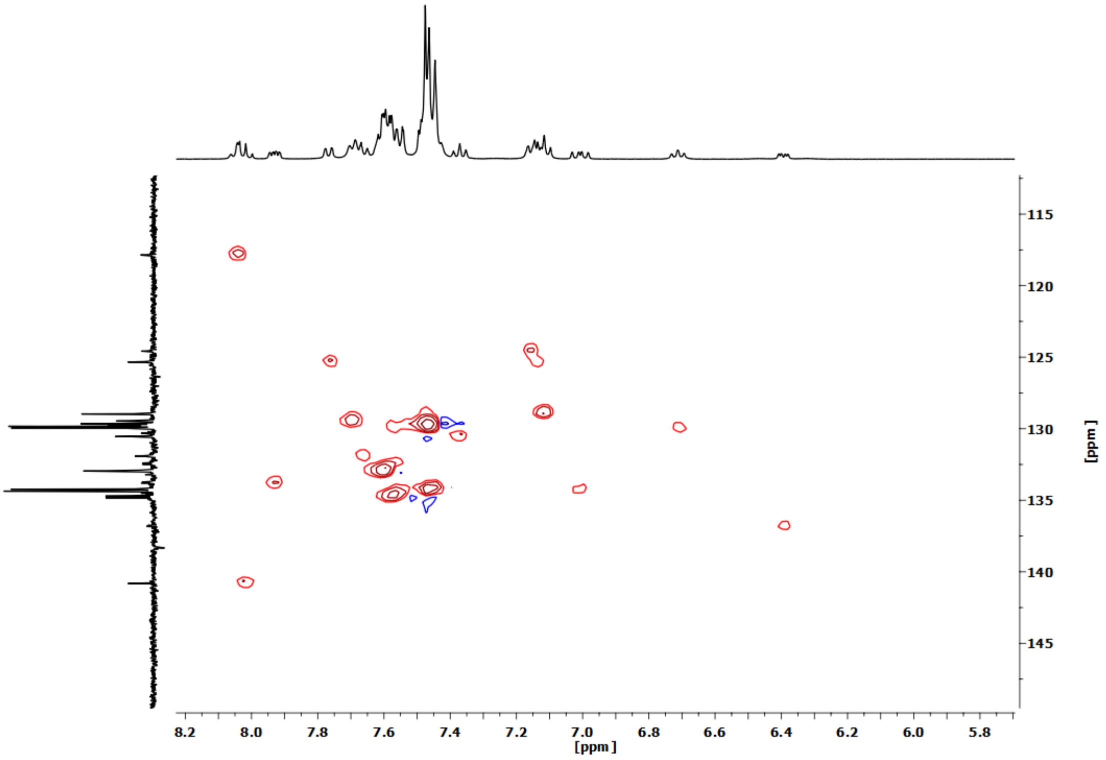

Figure S60. ^{1}H-^{13}C HSQC NMR spectrum (RT, CD_2Cl_2) of complex **7**.

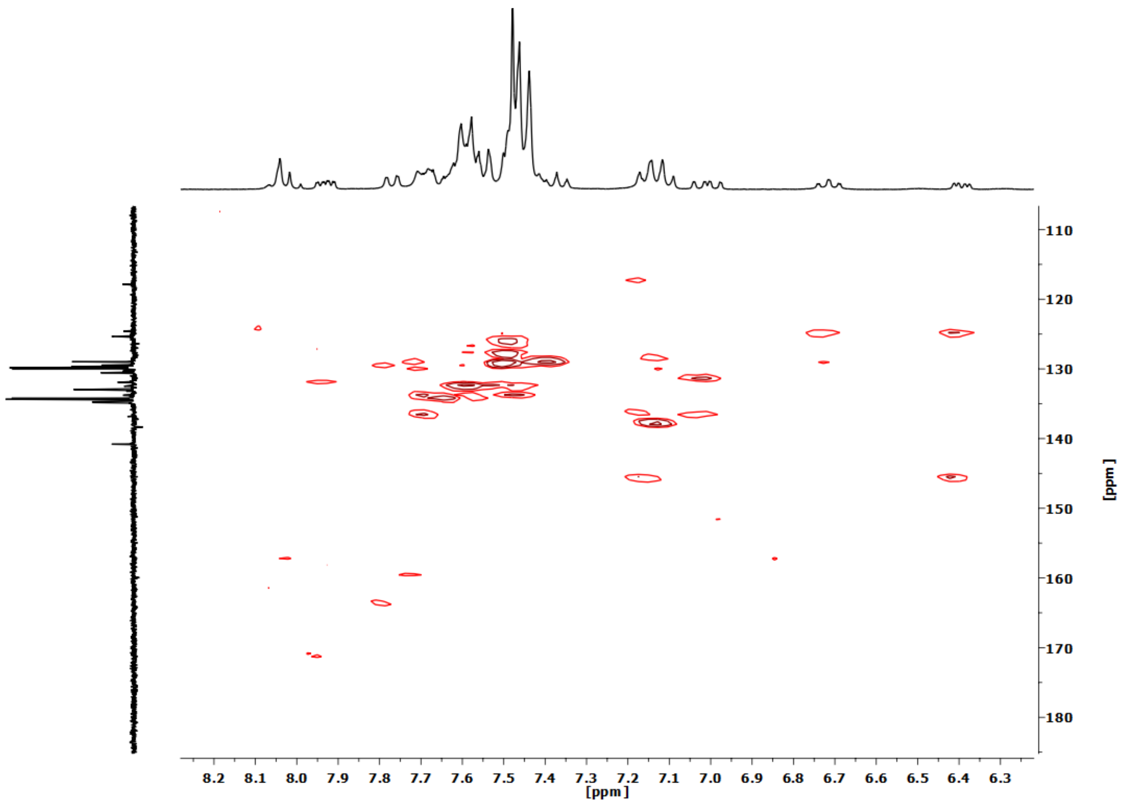

Figure S61. ^{1}H-^{13}C HMBC NMR spectrum (RT, CD_2Cl_2) of complex **7**.

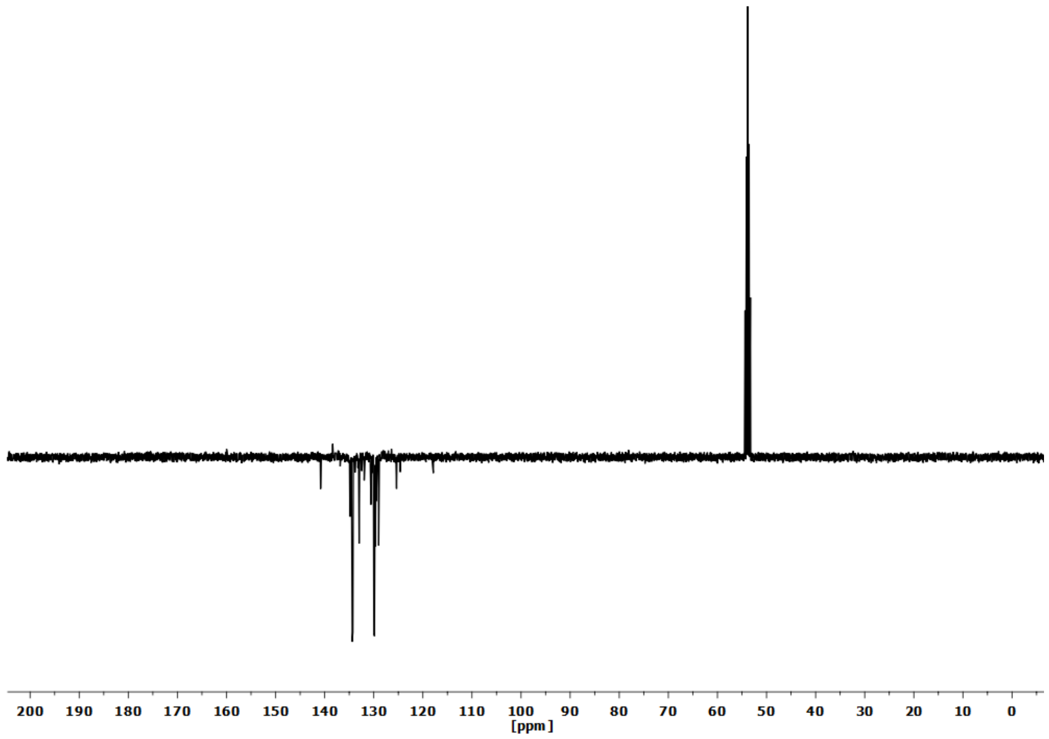

Figure S62. APT $^{13}C\{^{1}H\}$ NMR spectrum (RT, CD_2Cl_2) of complex **7**.

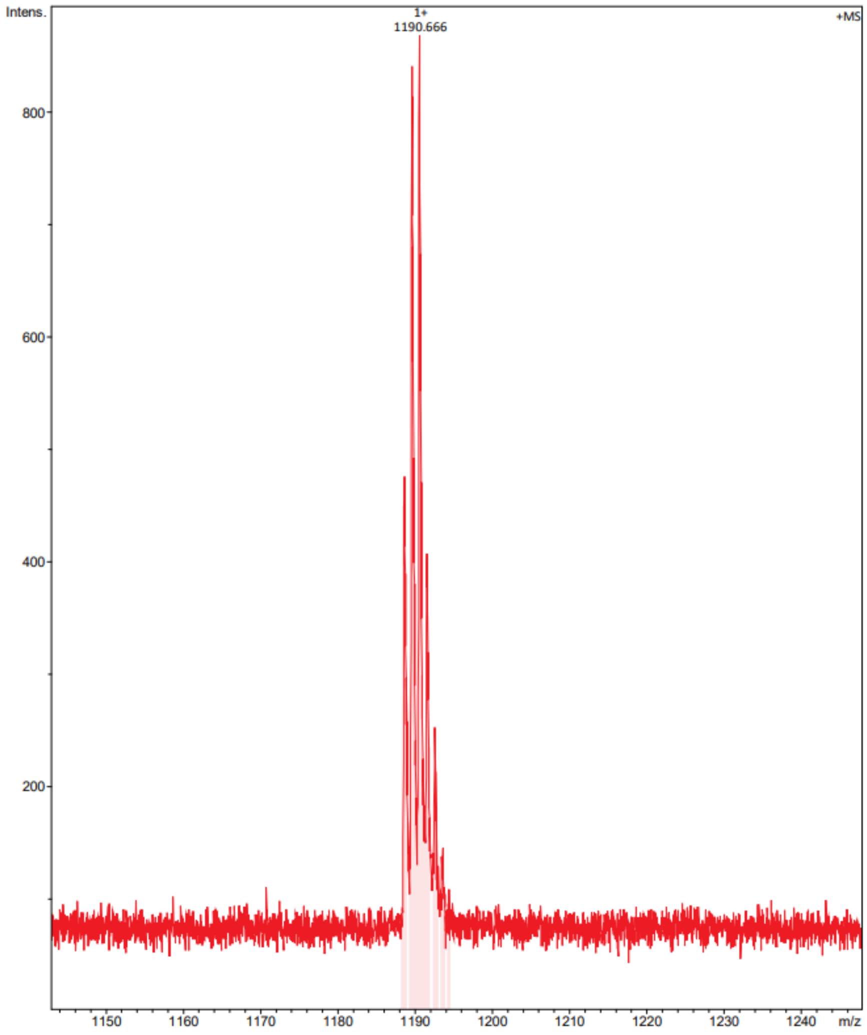

Figure S63. MS (MALDI+ DCTB) molecular peak of complex **7**.

2.8. Spectra of complex [{Pt(CNC-H){$PPh_2(C_6H_4$-*o*-O)}}Au(PPh_3)](ClO_4) (8)

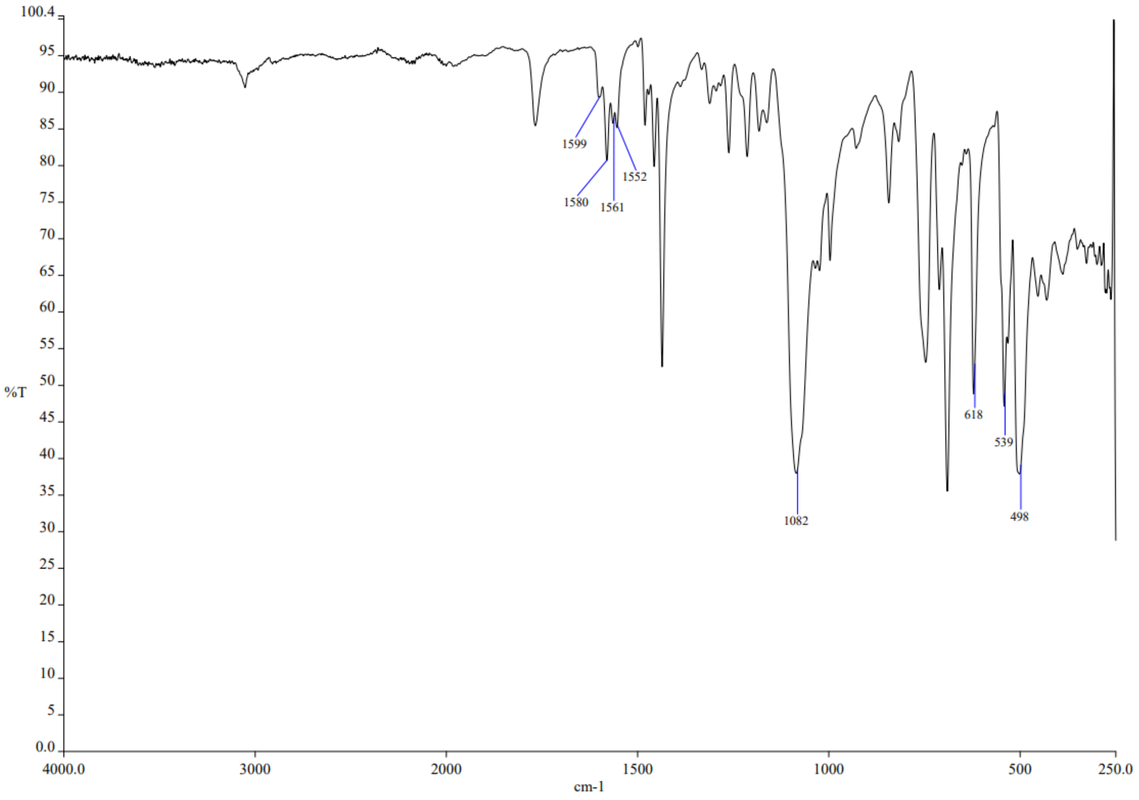

Figure S64. ATR-IR spectrum of complex **8**.

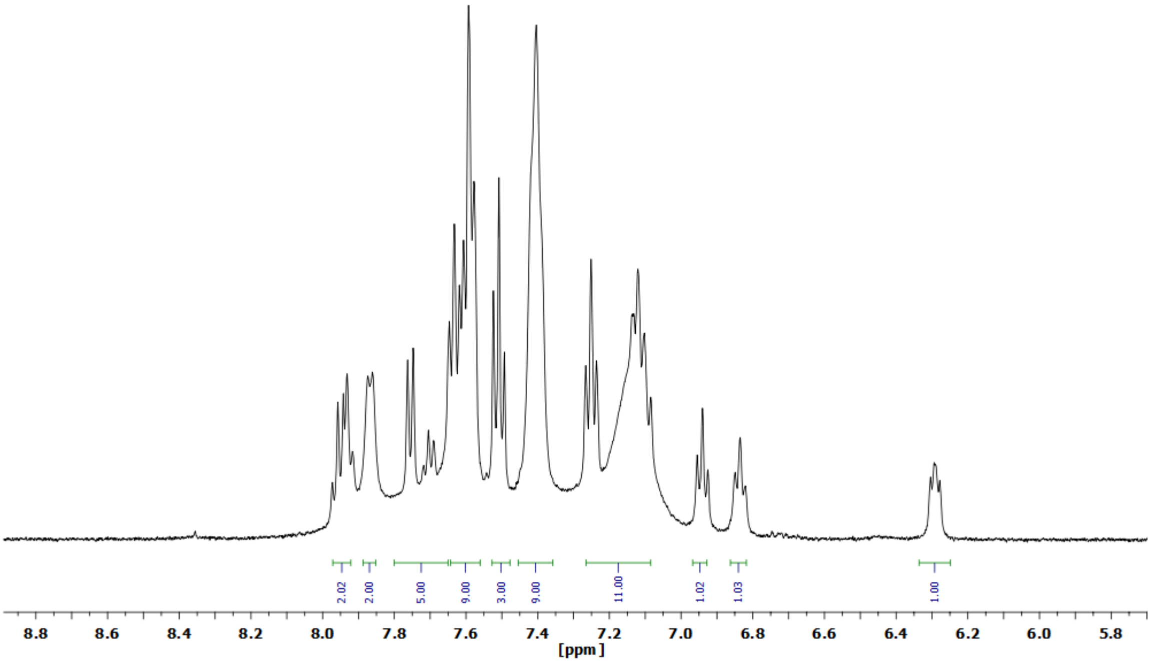

Figure S65. ^1H NMR spectrum (RT, CD_2Cl_2) of complex **8**.

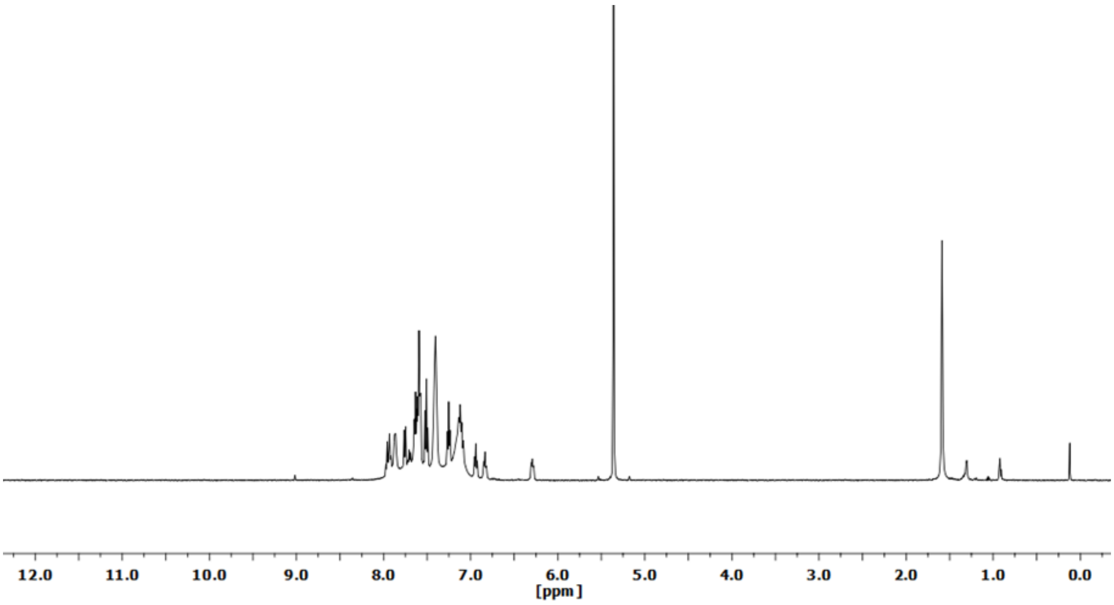

Figure S66. Full ^{1}H NMR spectrum (RT, CD_2Cl_2) of complex **8**.

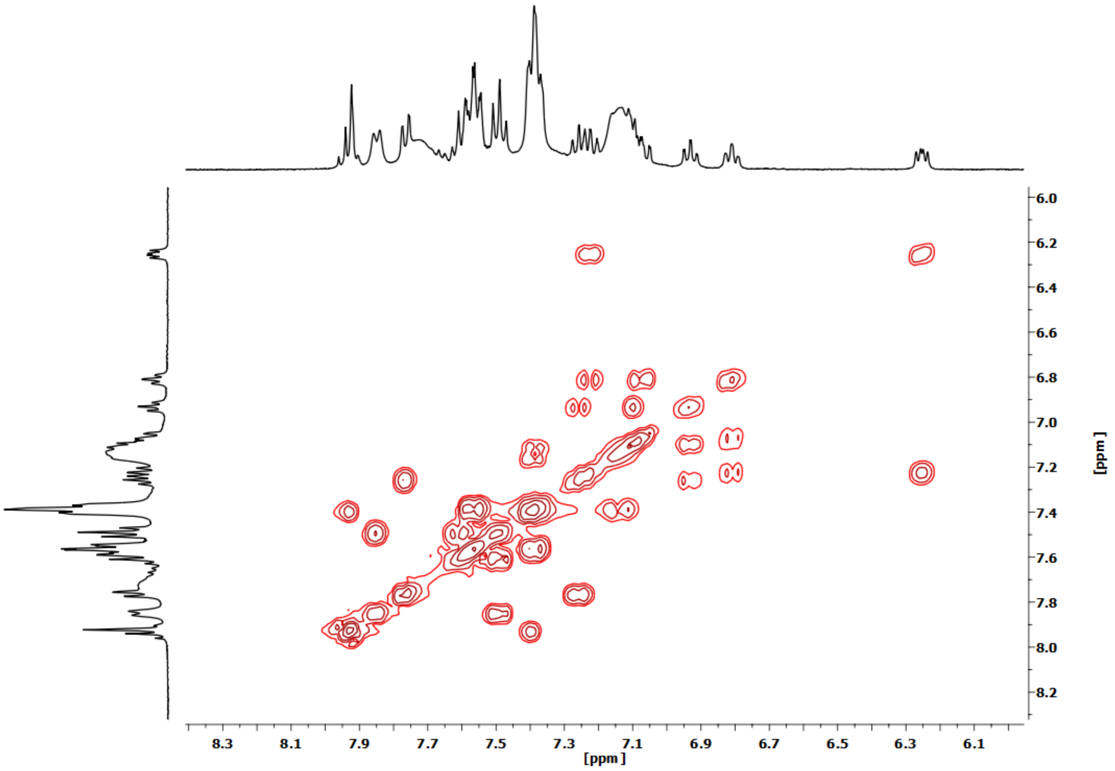

Figure S67. ^{1}H-^{1}H COSY NMR spectrum (RT, CD_2Cl_2) of complex **8.**

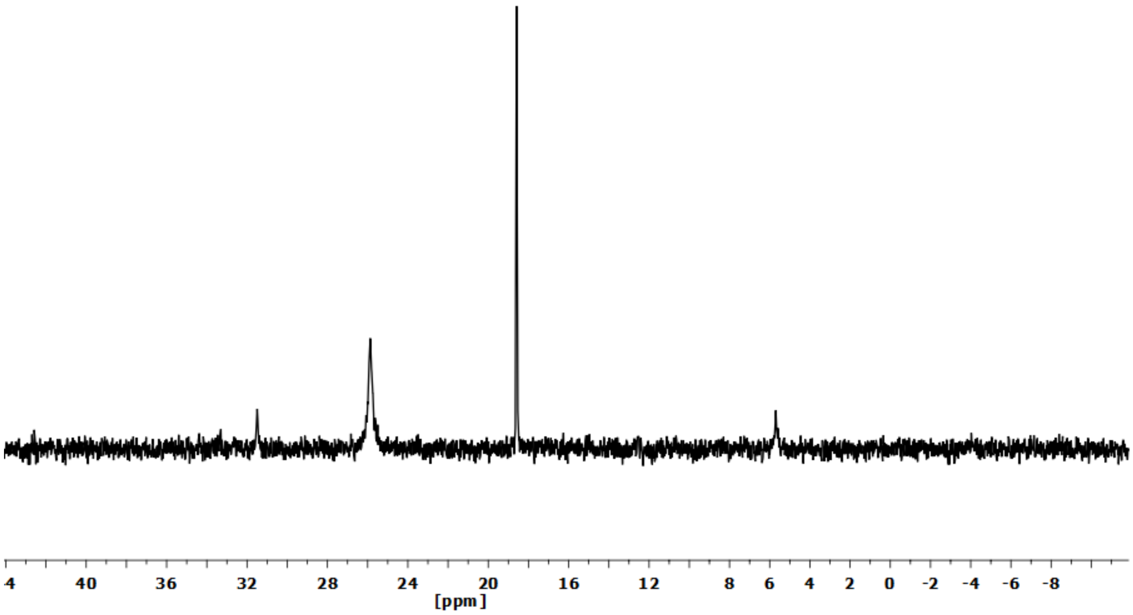

Figure S68. $^{31}P\{^{1}H\}$ NMR spectrum (RT, CD_2Cl_2) of complex **8**.

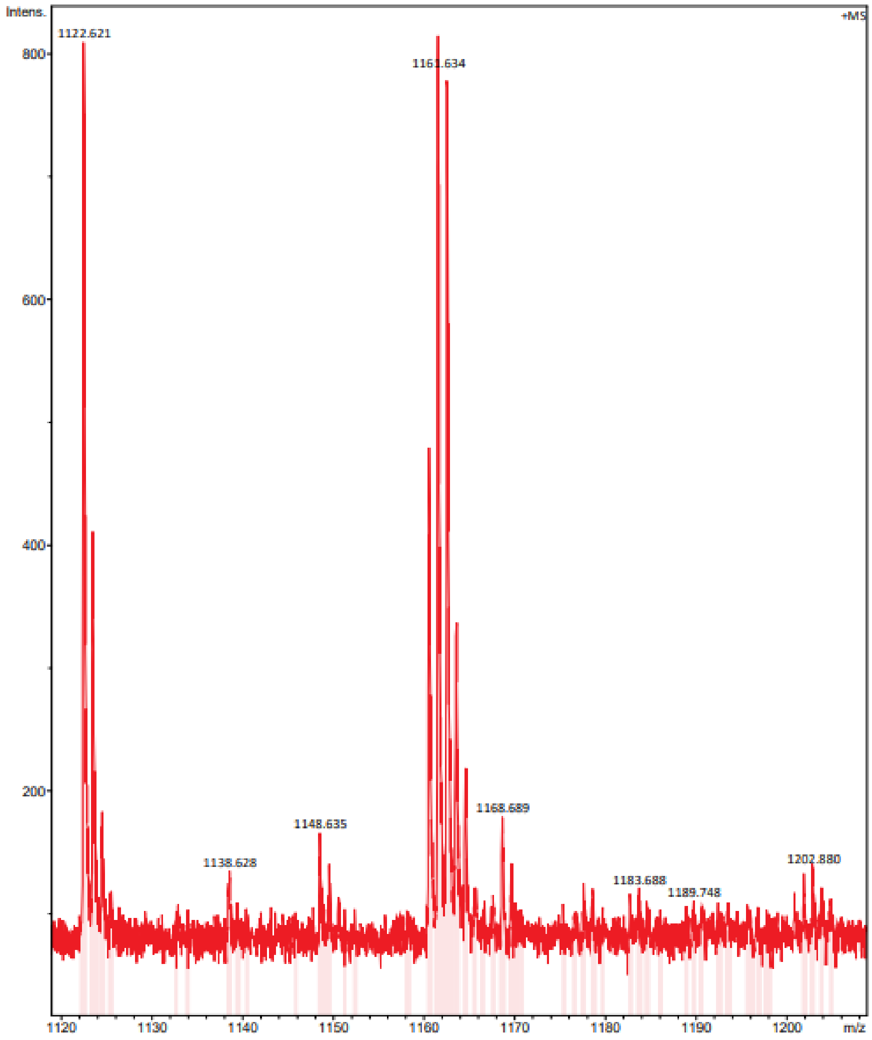

Figure S69. MS (MALDI+ DCTB) molecular peak of complex **8**.

2.9. Spectra of complex [{Pt(CNC-H){PPh2(C6H4-*o*-COOH)}}{Pt(CNC-H){PPh2(C6H4-*o*-COO)}}](ClO4) (9)

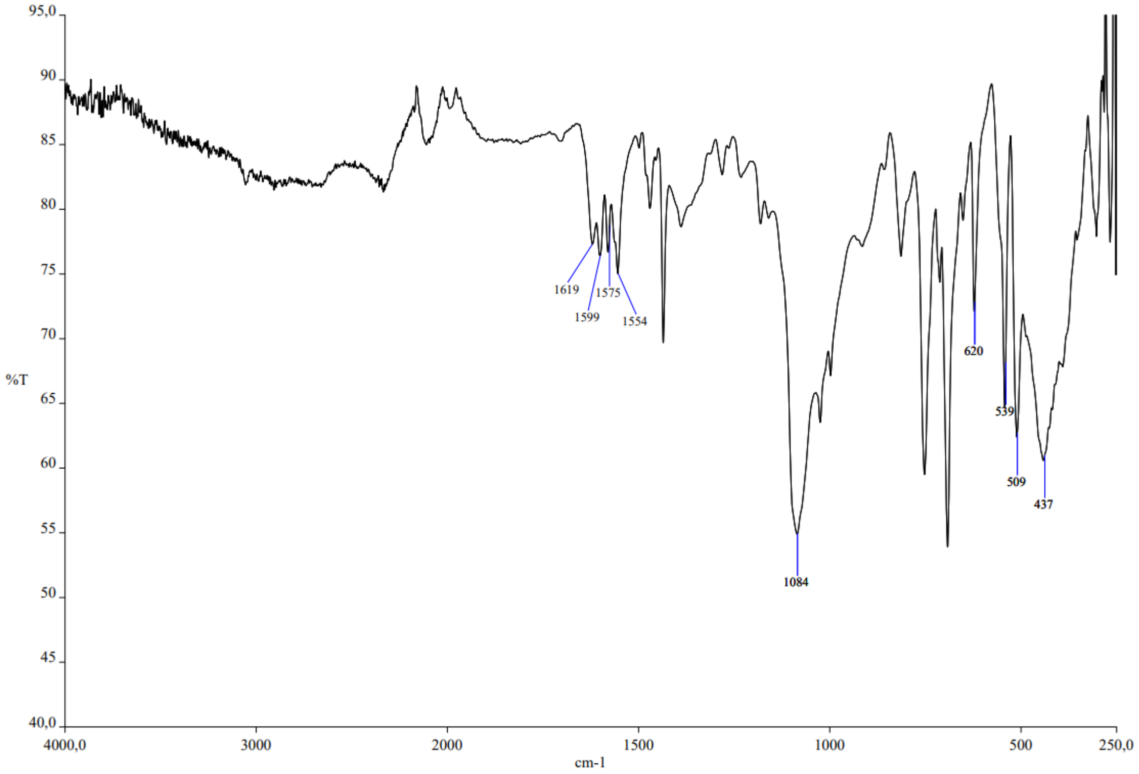

Figure S70. ATR-IR spectrum (RT, CD_2Cl_2) of complex **9**.

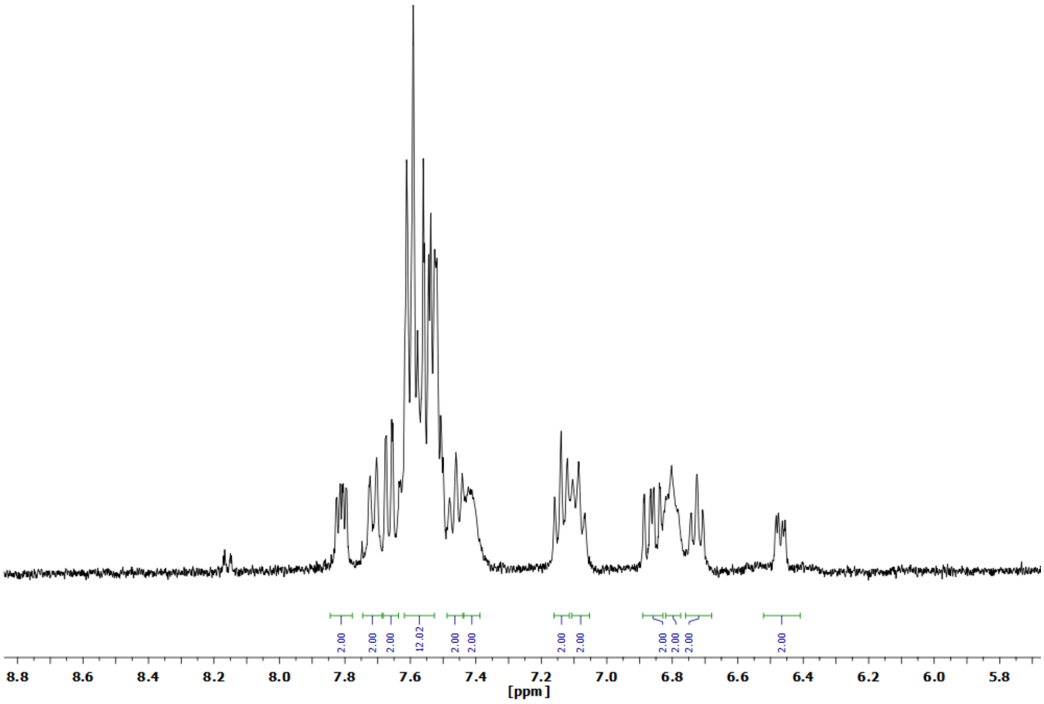

Figure S71. ^{1}H NMR spectrum (RT, CD_2Cl_2) of complex **9**.

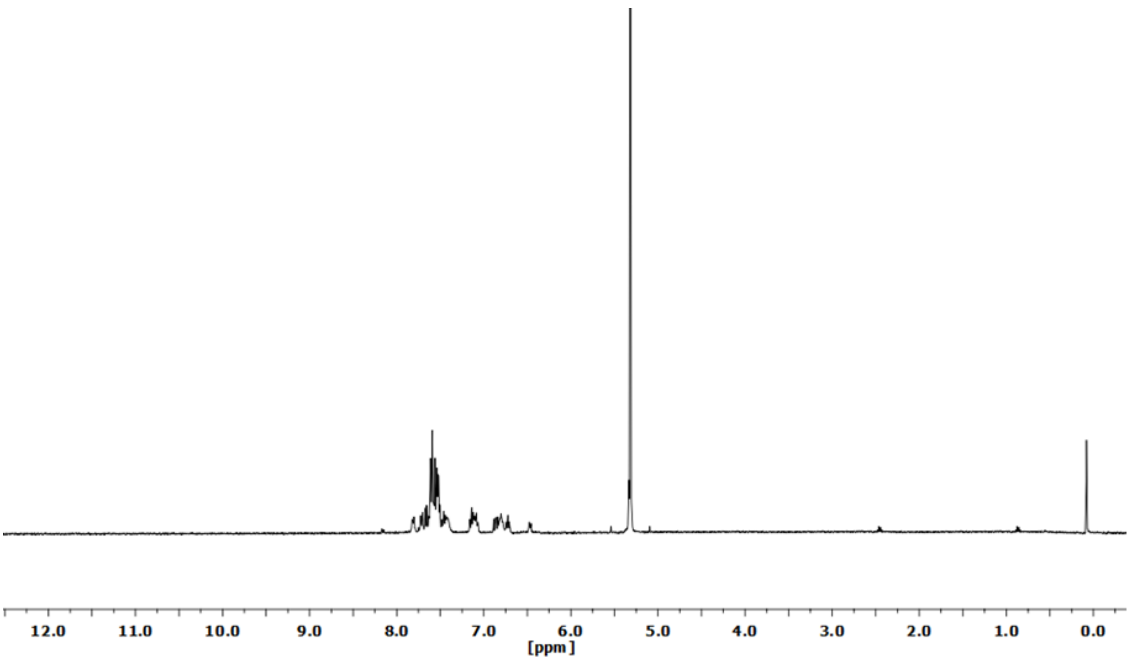

Figure S72. Full ^1H NMR spectrum (RT, CD_2Cl_2) of complex **9**.

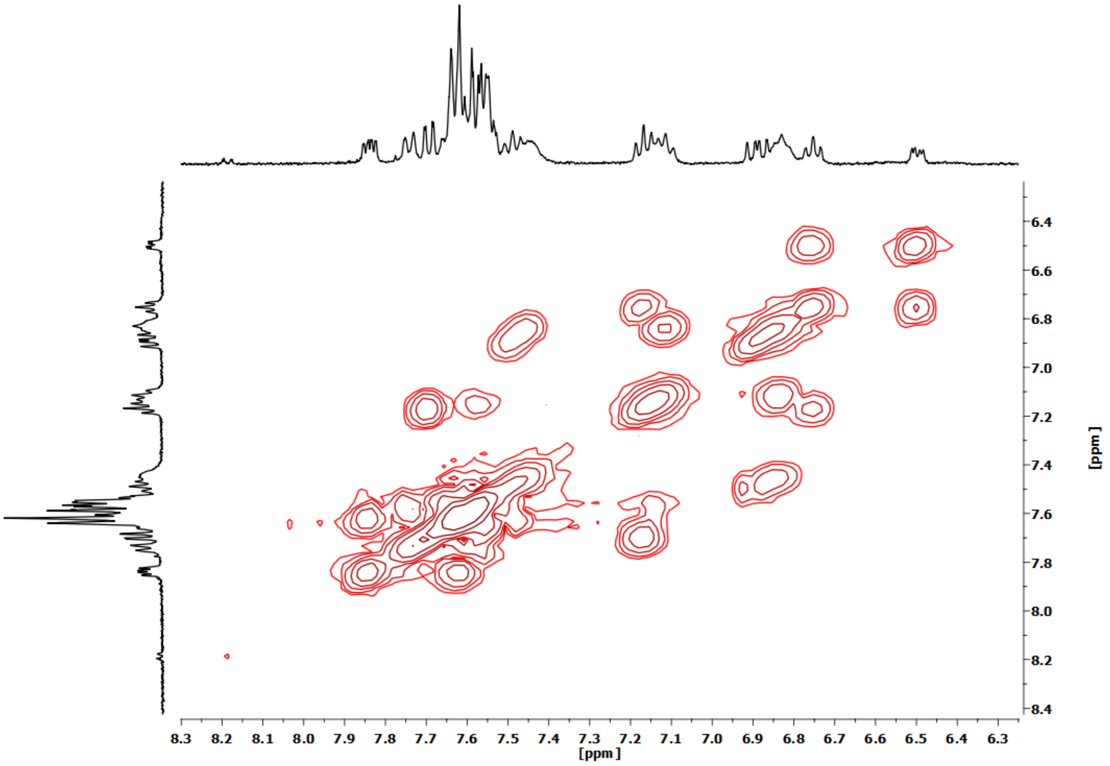

Figure S73. ^1H-^1H COSY NMR spectrum (RT, CD_2Cl_2) of complex **9**.

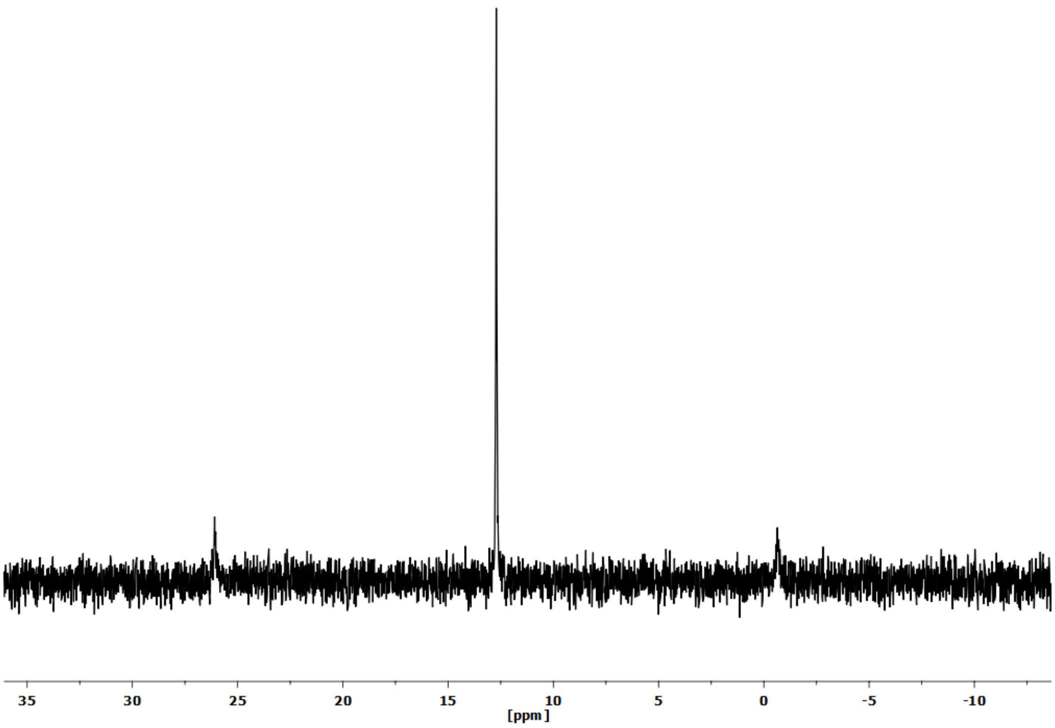

Figure S74. $^{31}P\{^{1}H\}$ NMR spectrum (RT, CD_2Cl_2) of complex **9**.

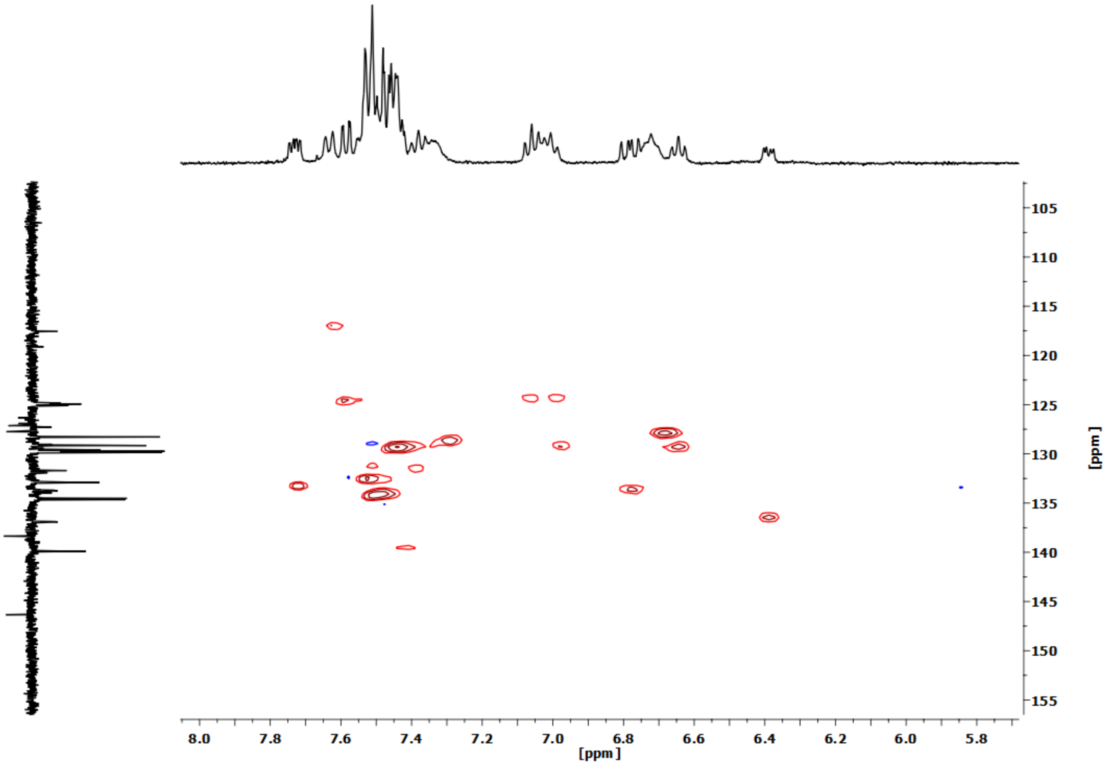

Figure S75. ^{1}H-^{13}C HSQC NMR spectrum (RT, CD_2Cl_2) of complex **9**.

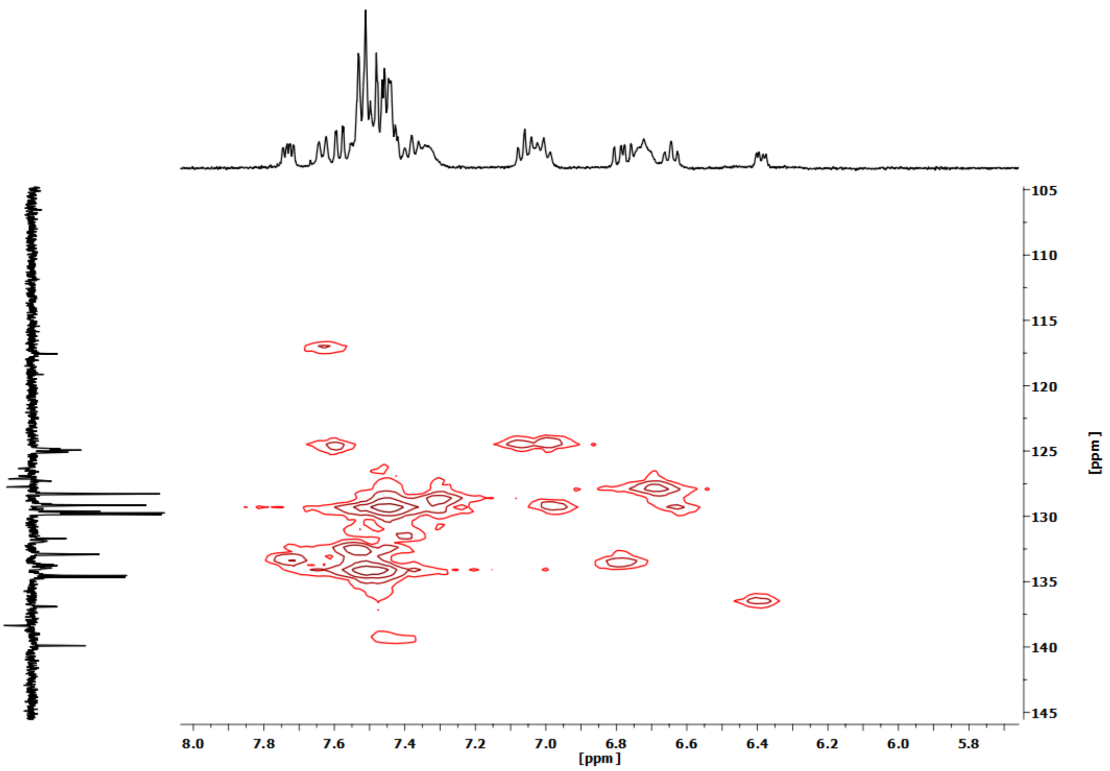

Figure S76. ^{1}H-^{13}C HMBC NMR spectrum (RT, CD_2Cl_2) of complex **9**.

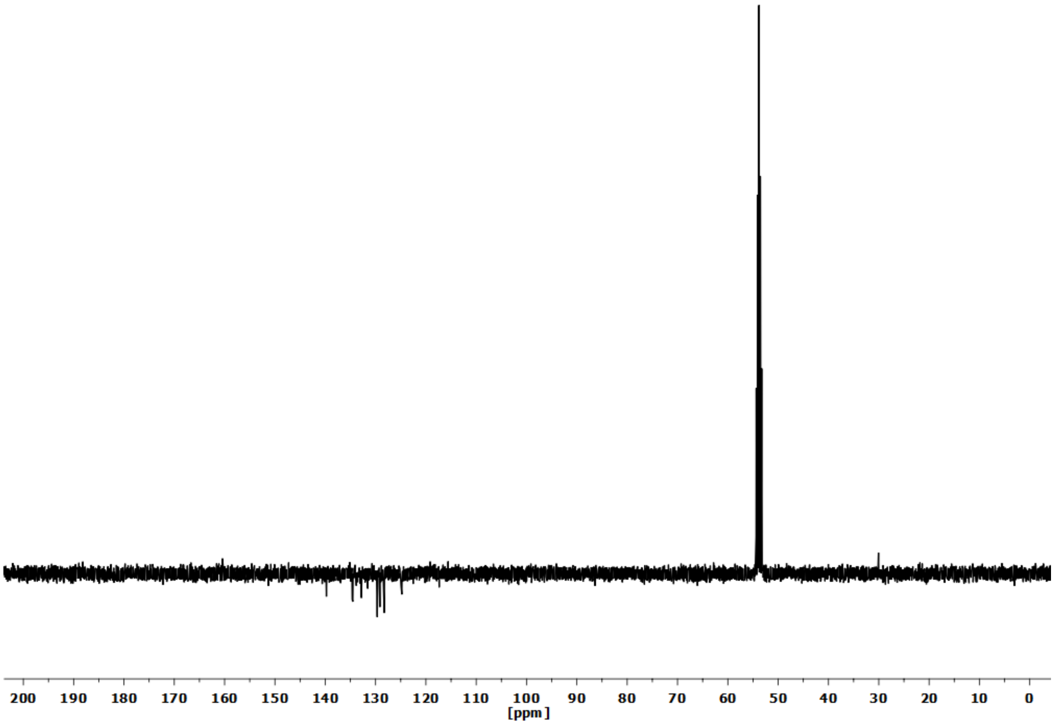

Figure S77. $^{13}C\{^{1}H\}$ APT NMR spectrum (RT, CD_2Cl_2) of complex **9**.

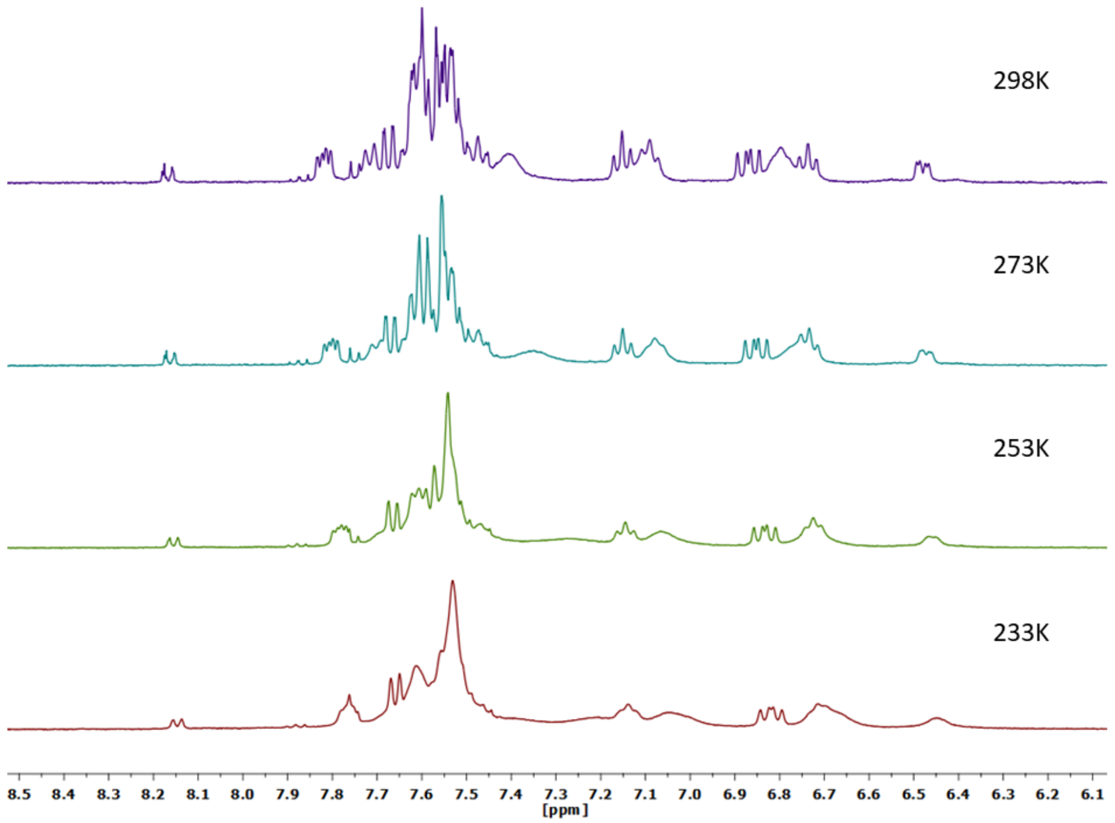

Figure S78. ^{1}H NMR spectrum (298K, 273K, 253K and 233K, CD_2Cl_2) of complex **9**.

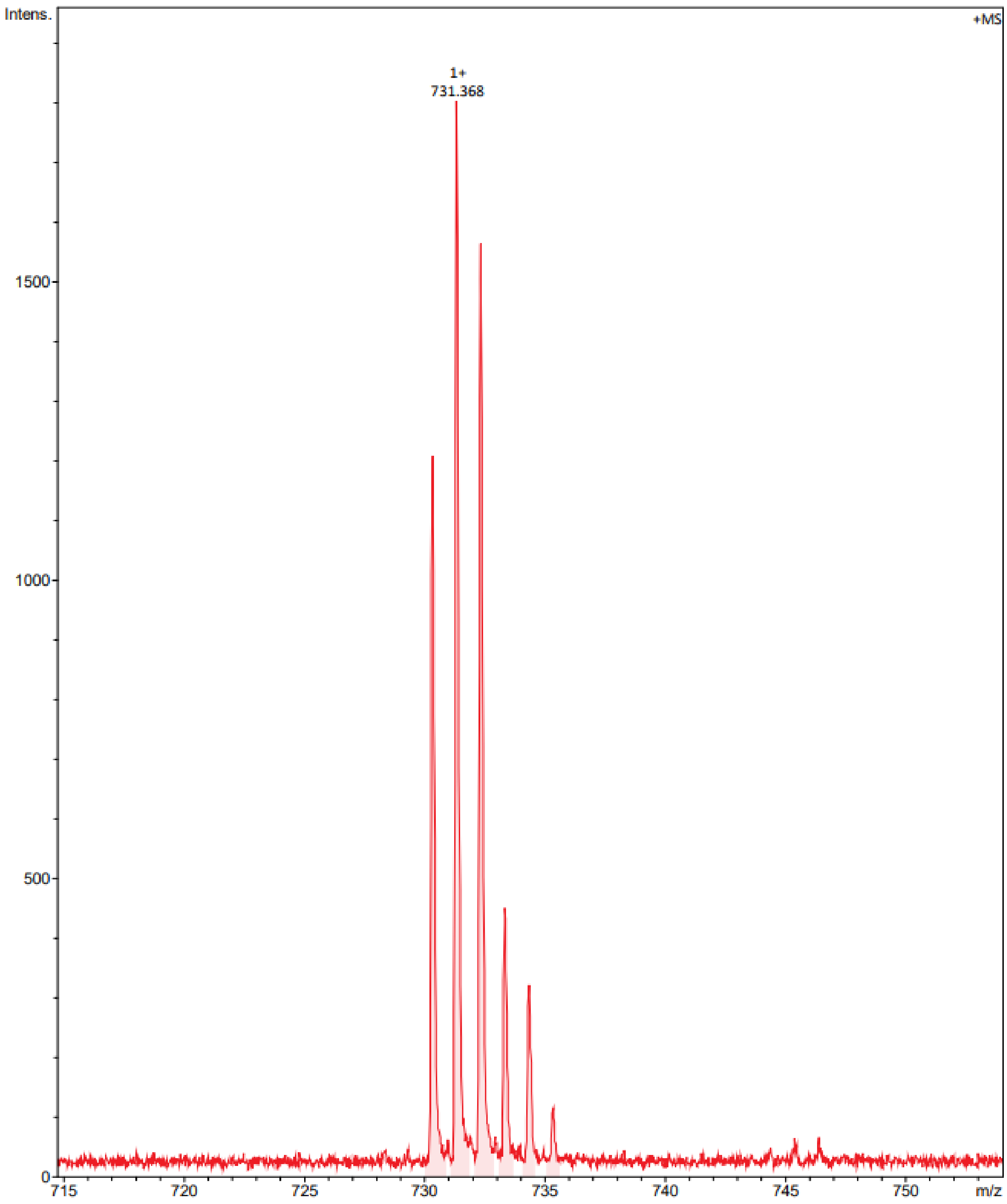

Figure S79. MS (MALDI+ DCTB) molecular peak of complex **9**.

2.10. Spectra of complex [{Pt(CNC-H){PPh2(C6H4-*o*-OH)}}{Pt(CNC-H){PPh2(C6H4-*o*-O)}}](ClO4) (10)

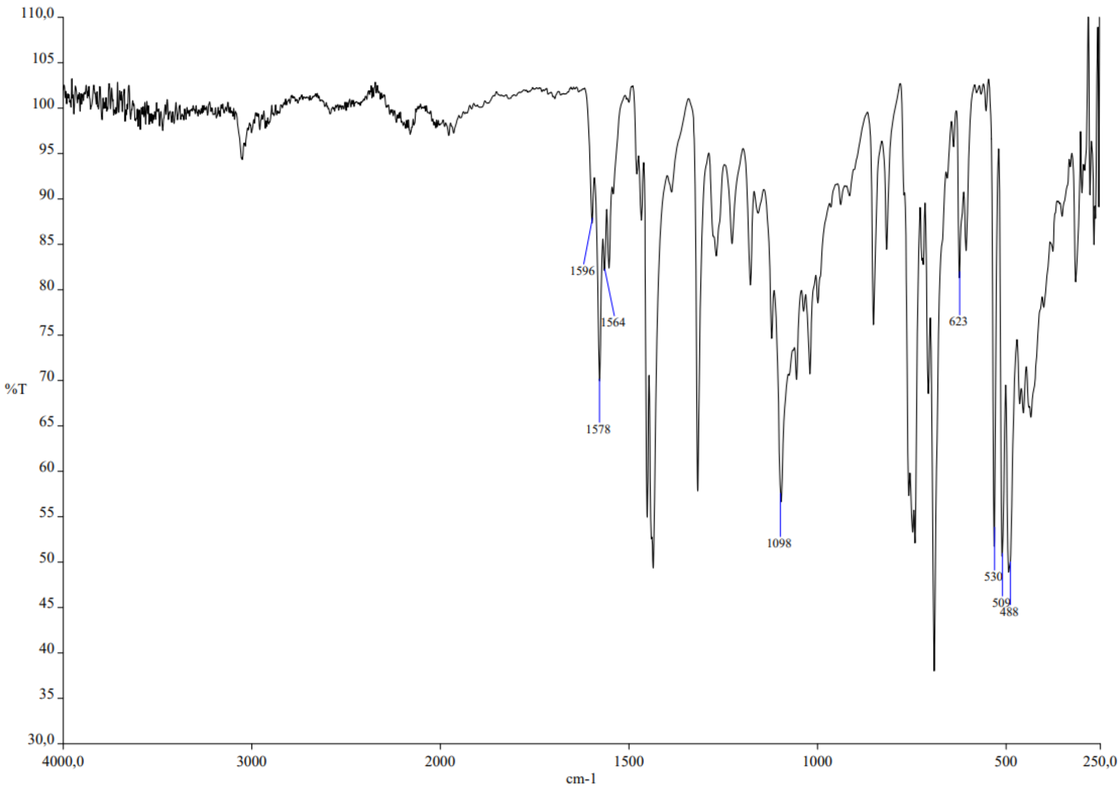

Figure S80. ATR-IR spectrum (RT, CD_2Cl_2) of complex **10**.

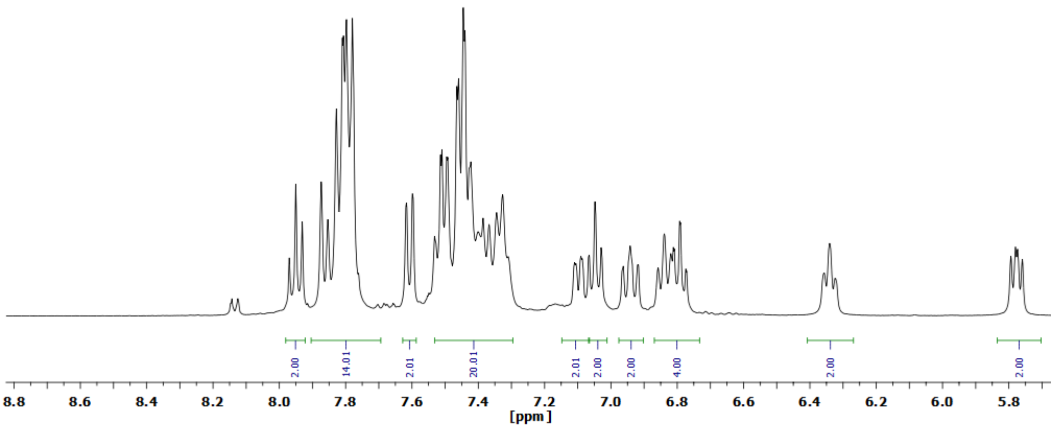

Figure S81. ^1H NMR spectrum (273K, CD_2Cl_2) of complex **10**.

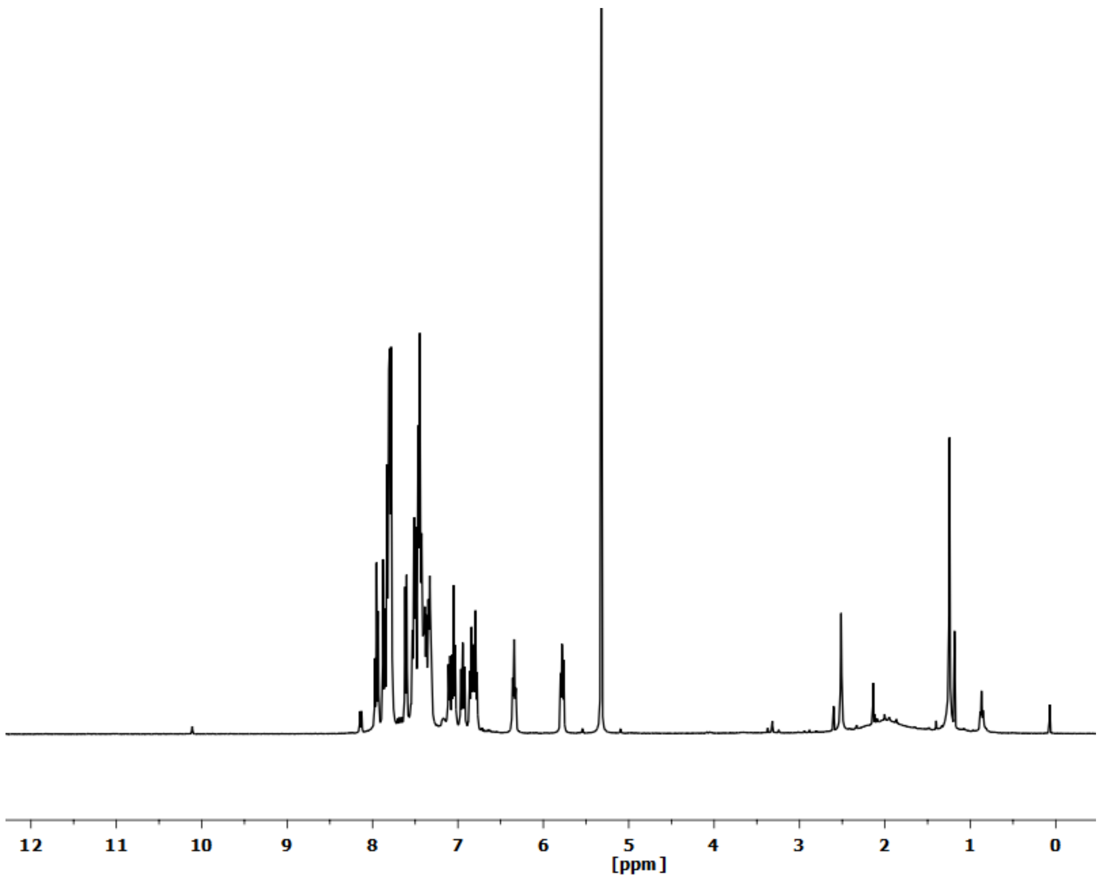

Figure S82. Full ^{1}H NMR spectrum (273K, CD_2Cl_2) of complex **10**.

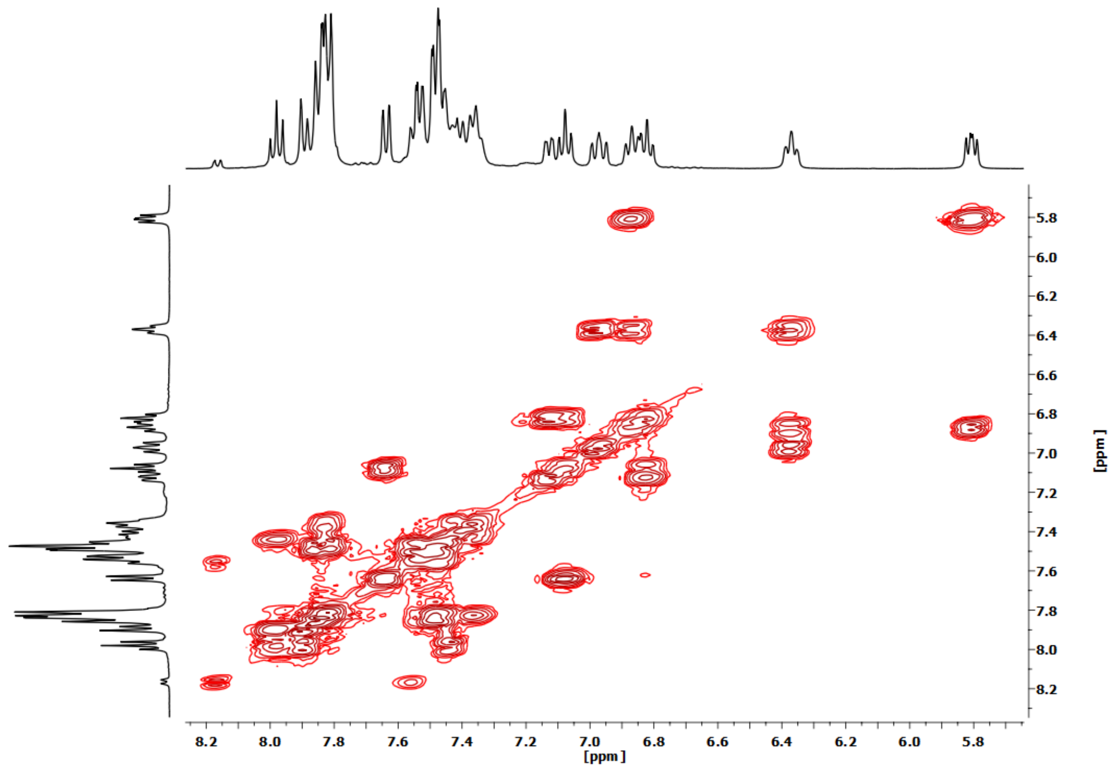

Figure S83. ^{1}H-^{1}H COSY NMR spectrum (273K, CD_2Cl_2) of complex **10**.

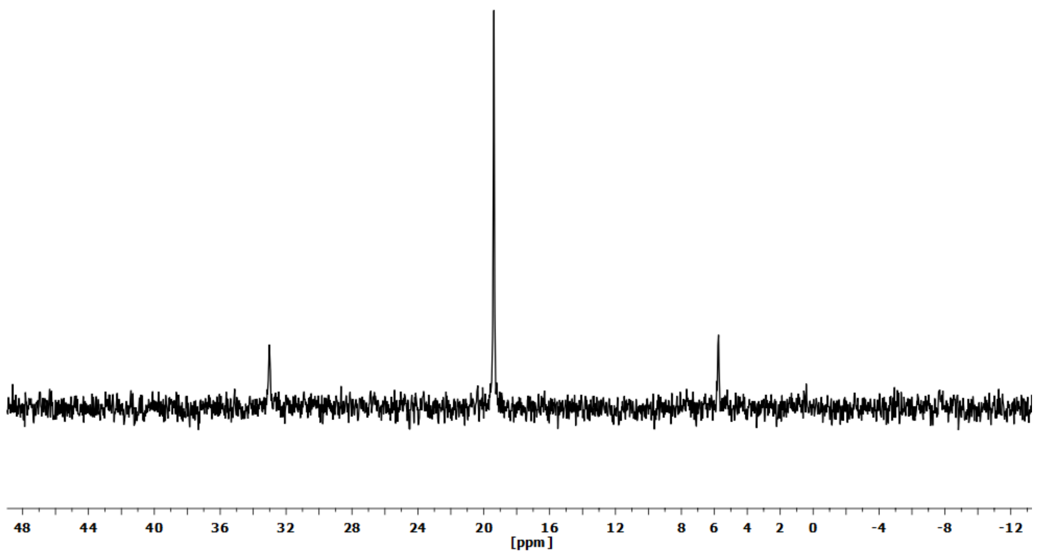

Figure S84. $^{31}P\{^{1}H\}$ NMR spectrum (273K, CD_2Cl_2) of complex **10**.

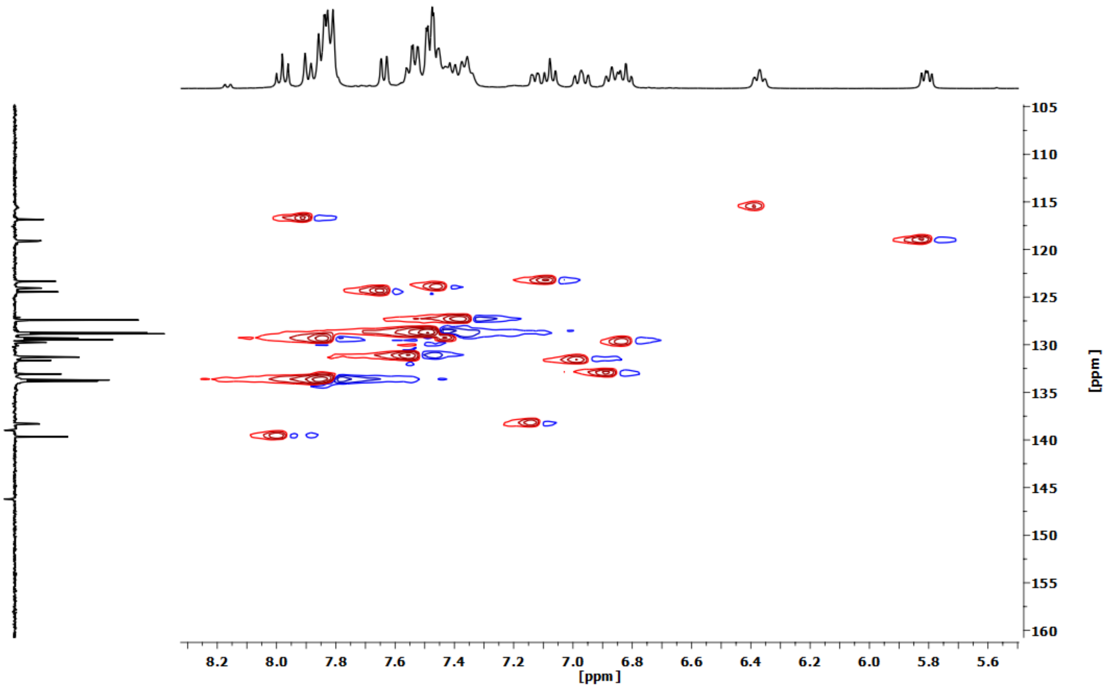

Figure S85. ^{1}H-^{13}C HSQC NMR spectrum (273K, CD_2Cl_2) of complex **10**.

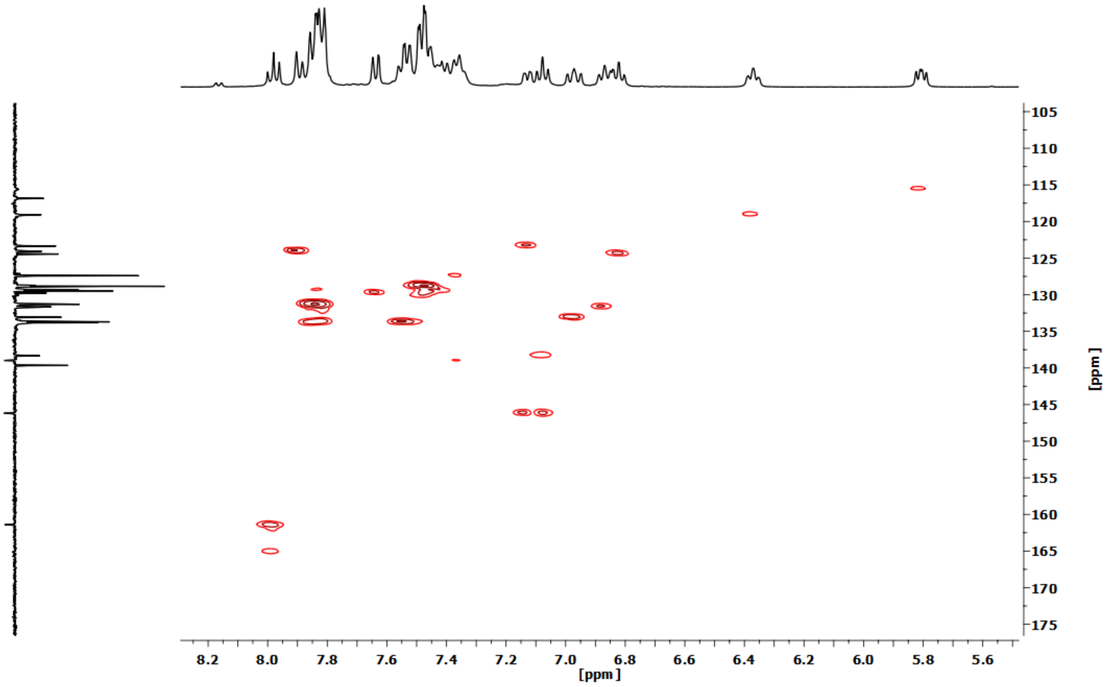

Figure S86. ^{1}H-^{13}C HMBC NMR spectrum (273K, CD_2Cl_2) of complex **10**.

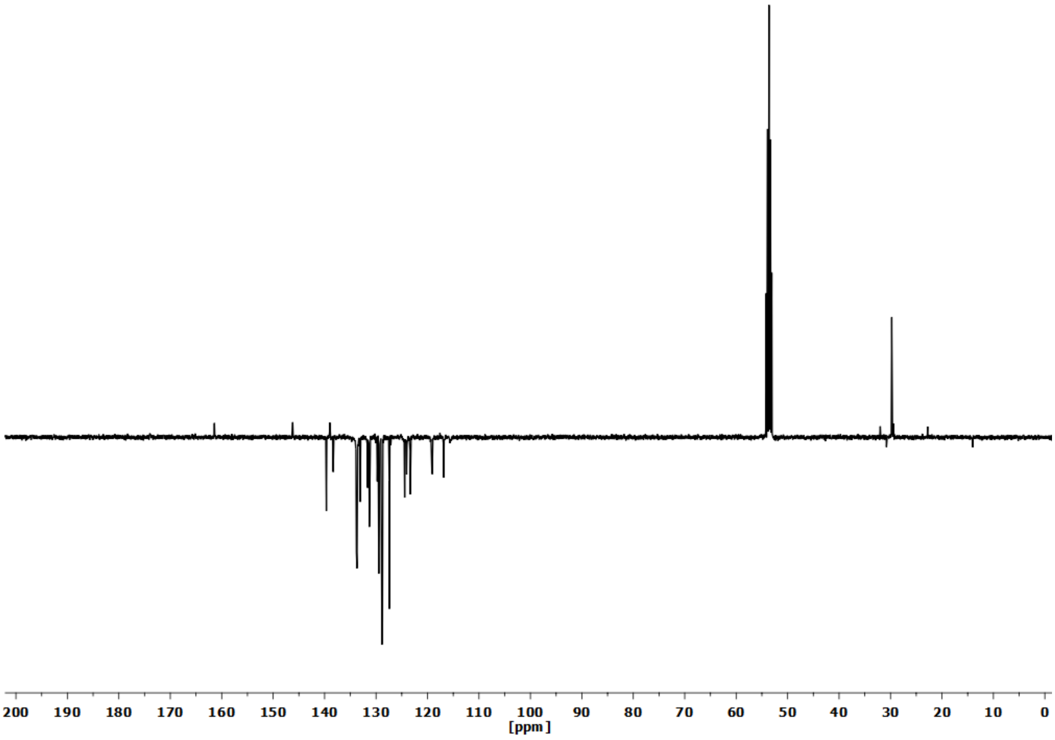

Figure S87. $^{13}C\{^{1}H\}$ APT NMR spectrum (273K, CD_2Cl_2) of complex **10**.

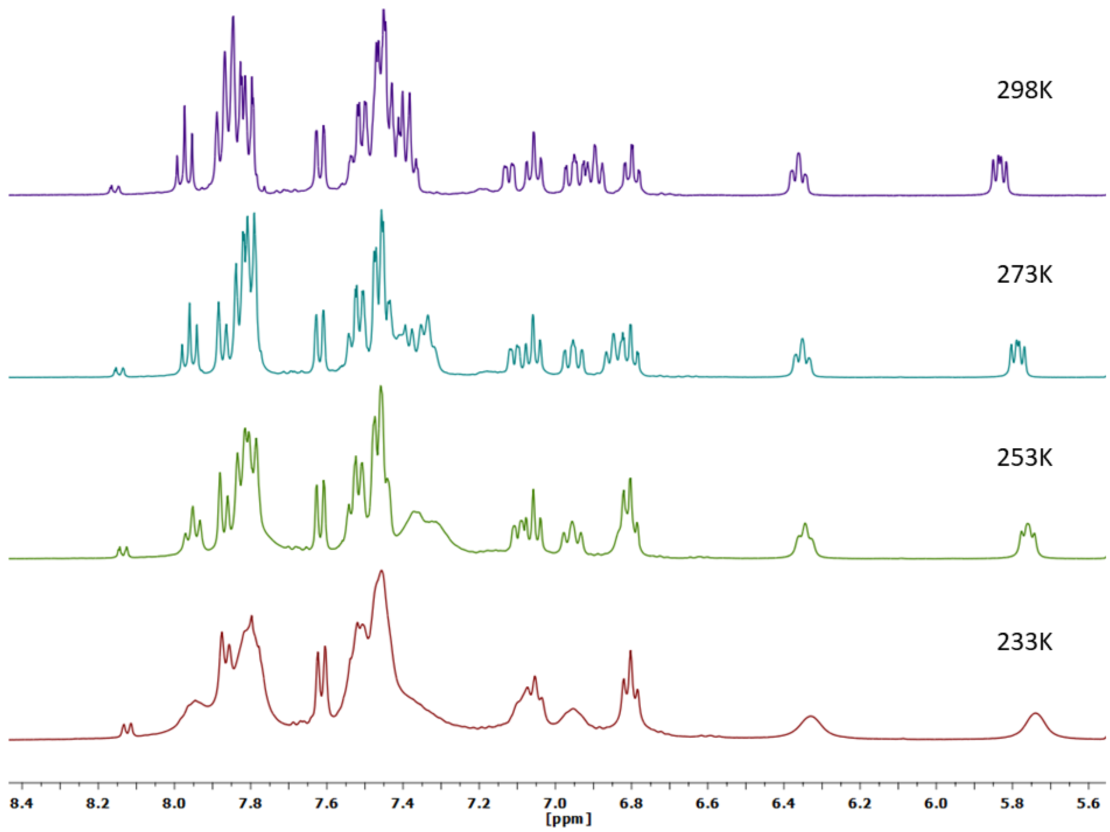

Figure S88. ^{1}H NMR spectrum (298K, 273K, 253K and 233K, CD_2Cl_2) of complex **10**.

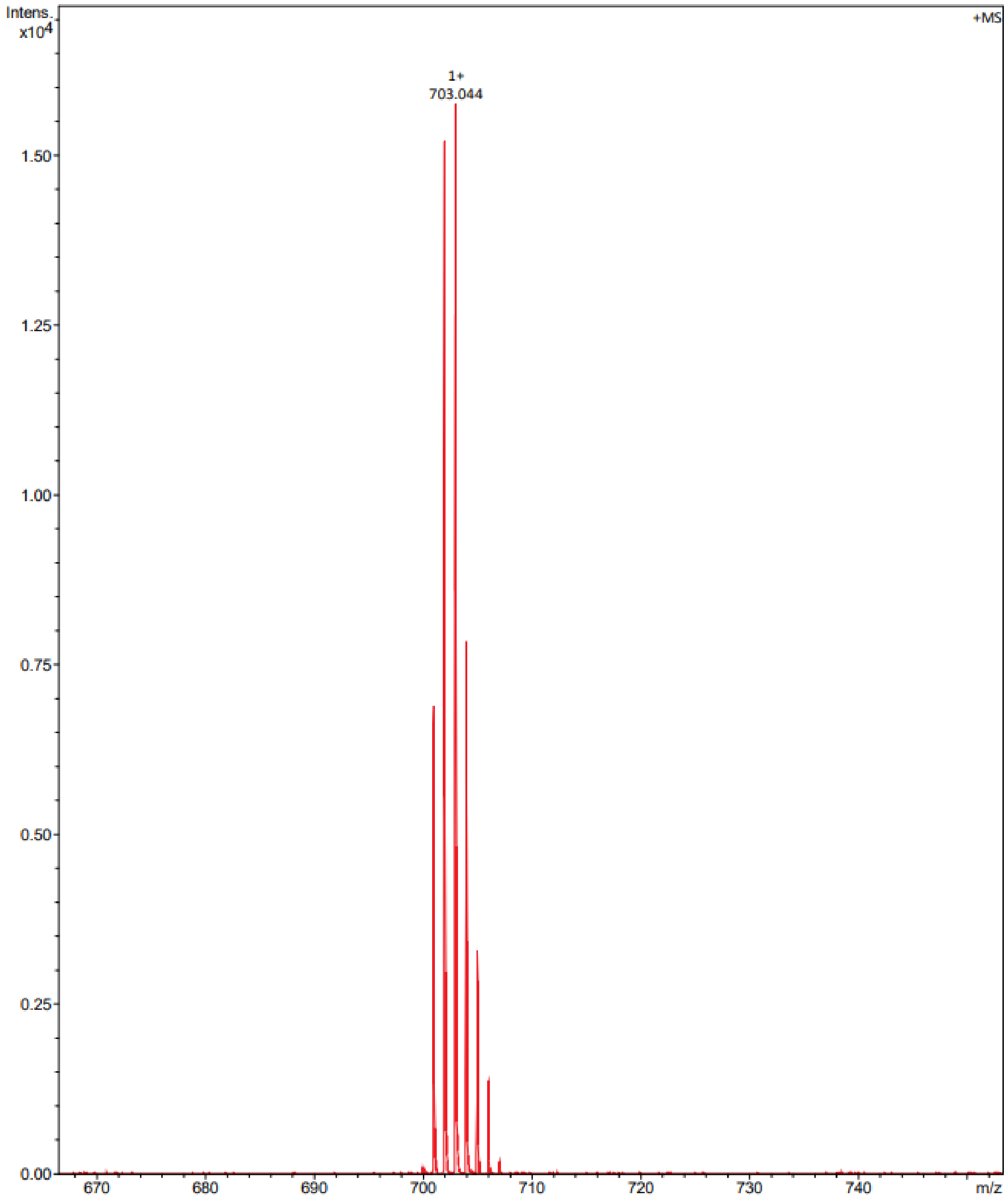

Figure S89. MS (MALDI+ DCTB) molecular peak of complex **10**.

3. Crystal data and structure refinement

Crystal data and other details of the structure analyses are presented in Tables S1 and S2. Suitable crystals for X-ray diffraction studies were obtained by slow diffusion of *n*-hexane into concentrated solutions of the complexes in 3 mL of CH_2Cl_2 or Me_2CO. Crystals were mounted at the end of quartz fibres. The radiation used in all cases was graphite monochromated MoK_α (λ = 0.71073 Å). X-ray intensity data were collected on an Oxford Diffraction Xcalibur diffractometer. The diffraction frames were integrated and corrected from absorption by using the CrysAlis RED program.[2]

The structures were solved by Patterson and Fourier methods and refined by full-matrix least squares on F^2 with SHELXL-97.[3] All non-hydrogen atoms were assigned anisotropic displacement parameters and refined without positional constraints, except as noted below. All hydrogen atoms were constrained to idealized geometries and assigned isotropic displacement parameters equal to 1.2 times the U_{iso} values of their attached parent atoms.

In the structure of **3**·0.5Me_2CO·0.5*n*-C_6H_{14}, the obtained crystals did not diffract very well and the data collected were rather weak. Also the packing of the molecules is not very efficient, so large voids are formed in the structure. In these voids, a rather diffuse solvent accommodated. After trying to model this solvent, only one molecule of acetone (with occupancy 0.5) and one half of *n*-hexane (the other half is generated by symmetry) could be defined. In both cases it was necessary to constrain the geometry with DFIX. The remaining electron density caused by the even more diffuse solvent was treated with SQUEEZE.[4] In any case some atoms show large and elongated thermal parameter, so for several of them ISOR was used.

In the structure of **5**·2CH_2Cl_2, one of the dichloromethane solvent molecule shows a disorder in one of its chlorine atoms which were modelled and refined with 0.9/0.1 partial occupancies.

For **6**·2 Me_2CO·0.5H_2O, it was not possible to find the positions of the hydrogen atoms of the water molecules in the electron density maps, and thus these atoms were not included in the final model.

For **9**·2CH_2Cl_2, the position of the hydrogen atom H1 bonded to O2 was found in the electron density maps and refined with no constrain in the O2–H1 distance and an isotropic displacement parameters equal to 1.2 times the U_{iso} values of O2.

For **10**·CH_2Cl_2, there are two molecules in the asymmetric unit that establish the hydrogen bridging system with another molecule generated from itself by a rotoinversor axis. Thus, there are two different O-H···O interactions. Furthermore, this situation produces that the interacting hydrogen atoms (H1O and H2O) are disordered over the two molecules and the ones generated by symmetry. The position of these hydrogen atoms, H1O and H2O, were found in the electron density maps and refined with a constrain in the O–H distance (0.84 Å) and 0.5 occupancy. Besides, the dichloromethane solvent molecule shows a disorder in one of its chlorine atoms which were modelled and refined with 0.66/0.33 partial occupancies.

Full-matrix least-squares refinement of these models against F^2 converged to final residual indices given in Tables S1 and S2.

Deposition Numbers <url href="https://www.ccdc.cam.ac.uk/services/structures?id=doi:10.1002/chem.202502370">2358548-2358554</url> contain the supplementary crystallographic data for this paper. These data are provided free of charge by the joint Cambridge Crystallographic Data Centre and Fachinformationszentrum Karlsruhe <url href="http://www.ccdc.cam.ac.uk/structures">Access Structures service</url>

Table S1. Crystal data and structure refinement for complexes **1**·CH_2Cl_2, **2**·CH_2Cl_2·and **3**·0.5Me_2CO·0.5*n*-C_6H_{14}.

	**1**·CH_2Cl_2	**2**·CH_2Cl_2	**3**·0.5Me_2CO·0.5*n*-C_6H_{14}
formula	$C_{36}H_{26}NO_2PPt$ ·CH_2Cl_2	$C_{35}H_{26}$ NOPPt ·CH_2Cl_2	$C_{100}H_{82}Ag_2Cl_2N_2O_{12}P_2Pt$ ·0.5Me_2CO·0.5*n*-C_6H_{14}
M_t [g mol^{-1}]	815.56	787.55	2472.58
T [K]	100(1)	100(1)	100(1)
λ [Å]	0.71073	0.71073	0.71073
crystal system	monoclinic	monoclinic	triclinic
space group	$P2_1/n$	$P2_1/c$	*P*-1
a [Å]	9.3284(2)	8.4494(2)	16.5544(4)
b [Å]	16.0093(3)	22.3359(5)	20.3648(5)
c [Å]	20.9697(4)	16.3739(4)	20.4528(6)
α [º]	90	90	117.218(3)
β [º]	90.372(2)	104.788(3)	90.401(2)
γ [º]	90	90	107.266(2)
V [Å^3]	3131.58(9)	2987.80(13)	5772.1(3)
Z	4	4	2
ρ [Mg m^{-3}]	1.730	1.751	1.423
μ [mm^{-1}]	4.738	4.960	2.908

$F(000)$	1600	1544	4900
2θ range [º]	5.4-56.9	6.0-61.3	4.8-57.0
no. of reflections collected	30952	22421	88931
no. of unique reflections	6772	8040	24559
R(int)	0.0405	0.0264	0.0965
final R indices [$I > 2\theta(I)$][a]			
R_1	0.0268	0.0236	0.0645
wR_2	0.0535	0.0522	0.1343
R indices (all data)			
R_1	0.0356	0.0286	0.1331
wR_2	0.0574	0.0542	0.1595
Goodness-of-fit on F^2[b]	1.026	1.029	1.018

[a] $R_1 = \sum(|F_o| - |F_c|) / \sum |F_o|$. $wR_2 = [\sum w (F_o^2 - F_c^2)^2 /\sum w(F_o^2)^2]^{1/2}$. [b] Goodness-of-fit = $[\sum w (F_o^2 - F_c^2)^2 / (n_{obs} - n_{param})]^{1/2}$.

Table S2. Crystal data and structure refinement for complexes **5**·2CH_2Cl_2, **6**·2 Me_2CO·0.5H_2O, **9**·2CH_2Cl_2 and **10**·CH_2Cl_2.

	**5**·2CH_2Cl_2	**6**·2 Me_2CO·0.5H_2O	**9**·2CH_2Cl_2	**10**·CH_2Cl_2
formula	$C_{72}H_{52}AgClN_2O_8P_2Pt_2$ ·**5**·2CH_2Cl_2	$C_{70}H_{62}AgClN_2O_6P_2Pt_2$ ·2 Me_2CO·0.5H_2O	$C_{72}H_{51}ClN_2O_8P_2Pt_2$ ·2CH_2Cl_2	$C_{70}H_{53}ClN_2O_6P_2Pt_2$ ·CH_2Cl_2
M_t [g mol^{-1}]	1838.44	1737.74	1731.58	1590.64
T [K]	100(1)	100(1)	100(1)	100(1)
λ [Å]	0.71073	0.71073	0.71073	0.71073
crystal system	monoclinic	monoclinic	monoclinic	monoclinic
space group	$P2_1/c$	$P2_1/n$	$P2_1/c$	$P2_1/c$
a [Å]	21.2977(4)	17.4738(2)	20.9006(2)	21.3945(2)
b [Å]	15.8124(2)	20.7850(2)	16.0549(1)	15.7511(1)
c [Å]	21.1289(3)	17.8716(3)	21.6399(2)	18.8525(2)
β [º]	110.670(2)	91.2114(12)	114.8118(11)	108.8552(11)
V [Å^3]	6657.47(19)	6489.41(14)	6591.1(1)	6012.12(10)
Z	4	4	4	4
ρ [Mg m^{-3}]	1.834	1.779	1.745	1.757
μ [mm^{-1}]	4.792	4.752	4.551	4.893
F(000)	3584	3412	3400	3120
2θ range [º]	4.9-56.9	3.3-55.8	4.3-59.0	4.4-56.9

no. of reflections collected	92039	121352	149346	181857
no. of unique reflections	14894	14354	17192	14329
R(int)	0.0600	0.0898	0.0408	0.0456
final *R* indices [$I > 2\theta(I)$][a]				
R_1	0.0344	0.0400	0.0240	0.0317
wR_2	0.0599	0.0734	0.0469	0.0746
R indices (all data)				
R_1	0.0563	0.0685	0.0303	0.0392
wR_2	0.0658	0.0835	0.0490	0.0778
Goodness-of-fit on F^2[b]	1.022	1.024	1.034	1.068

[a] $R_1 = \sum(|F_o| - |F_c|) / \sum |F_o|$. $wR_2 = [\sum w (F_o^2 - F_c^2)^2 / \sum w(F_o^2)^2]^{1/2}$. [b] Goodness-of-fit = $[\sum w (F_o^2 - F_c^2)^2 / (n_{obs} - n_{param})]^{1/2}$.

Table S3. Selection of bond lengths (Å) and angles (º) for **1** and **2**.

	**1**	**2**
Pt–N	2.101(3)	2.132(2)
Pt–C7	1.990(3)	1.993(2)
Pt–P	2.2067(8)	2.2044(6)
Pt–O	2.103(2)	2.101(2)
C7–Pt–N	81.76(6)	81.29(9)
C7–Pt–O	162.41(11)	173.58(8)
P–Pt–O	84.67(6)	83.43(5)

Table S4. Selection of bond lengths (Å) and angles (º) for **3**.

Pt(1)–C(7)	1.991(8)	Pt(1)–O(1)	2.101(5)
Pt(1)–N(1)	2.101(6)	Pt(1)–P(1)	2.214(2)
Ag(1)–O(2)	2.237(5)	Ag(1)–P(2)	2.361(2)
Ag(1)–O(3)	2.502(7)	Pt(2)–C(61)	2.013(9)
Pt(2)–O(7)	2.103(6)	Pt(2)–N(2)	2.124(7)
Pt(2)–P(3)	2.207(2)	Ag(2)–O(8)	2.223(6)
Ag(2)–P(4)	2.352(3)		
C(7)–Pt(1)–O(1)	161.2(3)	C(7)–Pt(1)–N(1)	80.8(3)
O(1)–Pt(1)–N(1)	93.6(2)	C(7)–Pt(1)–P(1)	101.3(3)
O(1)–Pt(1)–P(1)	85.91(15)	N(1)–Pt(1)–P(1)	174.78(19)
O(2)–Ag(1)–P(2)	154.29(16)	O(2)–Ag(1)–O(3)	81.4(2)
P(2)–Ag(1)–O(3)	118.51(16)	C(24)–O(2)–Ag(1)	106.2(5)
Cl(1)–O(3)–Ag(1)	116.9(3)		

Table S5. Selection of bond lengths (Å) and angles (º) for **5**.

Pt(1)–C(7)	1.996(4)	Pt(1)–O(1)	2.120(3)
Pt(1)–N(1)	2.128(3)	Pt(1)–P(1)	2.2136(10)
Pt(2)–C(43)	1.994(4)	Pt(2)–N(2)	2.132(3)
Pt(2)–O(3)	2.133(3)	Pt(2)–P(2)	2.2142(10)
Ag–O(4)	2.203(3)	Ag–O(2)	2.205(3)
C(7)–Pt(1)–O(1)	165.09(14)	C(7)–Pt(1)–N(1)	81.20(15)
O(1)–Pt(1)–N(1)	95.37(12)	C(7)–Pt(1)–P(1)	99.54(12)
O(1)–Pt(1)–P(1)	84.75(7)	N(1)–Pt(1)–P(1)	176.58(9)
C(43)–Pt(2)–N(2)	81.16(14)	C(43)–Pt(2)–O(3)	164.78(14)
N(2)–Pt(2)–O(3)	96.69(11)	C(43)–Pt(2)–P(2)	101.03(12)
N(2)–Pt(2)–P(2)	174.84(9)	O(3)–Pt(2)–P(2)	82.42(8)
O(4)–Ag–O(2)	142.86(11)		

Table S6. Selection of bond lengths (Å) and angles (º) for **6**.

Pt(1)–C(7)	1.989(5)	Pt(1)–N(1)	2.131(4)
Pt(1)–O(1)	2.144(3)	Pt(1)–P(1)	2.2050(14)
Pt(2)–C(42)	1.987(6)	Pt(2)–N(2)	2.138(4)
Pt(2)–O(2)	2.152(3)	Pt(2)–P(2)	2.2108(13)
Ag–O(2)	2.258(3)	Ag–O(1)	2.337(3)
Ag–C(13)	2.424(5)	Ag–C(48)	2.531(5)
C(7)–Pt(1)–N(1)	80.41(19)	C(7)–Pt(1)–O(1)	173.53(16)
N(1)–Pt(1)–O(1)	98.56(14)	C(7)–Pt(1)–P(1)	97.57(16)
N(1)–Pt(1)–P(1)	177.30(12)	O(1)–Pt(1)–P(1)	83.24(10)
C(42)–Pt(2)–N(2)	80.50(19)	C(42)–Pt(2)–O(2)	174.17(17)
N(2)–Pt(2)–O(2)	97.77(14)	C(42)–Pt(2)–P(2)	99.78(15)
N(2)–Pt(2)–P(2)	179.67(12)	O(2)–Pt(2)–P(2)	81.94(9)
O(2)–Ag–O(1)	126.07(13)	O(2)–Ag–C(13)	122.66(15)
O(1)–Ag–C(13)	101.33(15)	O(2)–Ag–C(48)	95.19(15)
O(1)–Ag–C(48)	85.99(15)	C(13)–Ag–C(48)	120.89(19)

Table S7. Selection of bond lengths (Å) and angles (º) for **9**.

Pt(1)–C(7)	1.992(2)	Pt(1)–N(1)	2.0912(19)
Pt(1)–O(1)	2.1341(16)	Pt(1)–P(1)	2.2091(6)
O(1)–C(24)	1.241(3)	O(2)–C(24)	1.287(3)
O(2)–H(1)	0.84(3)	Pt(2)–C(43)	1.990(2)
Pt(2)–N(2)	2.1112(19)	Pt(2)–O(3)	2.1206(16)
Pt(2)–P(2)	2.2086(6)		
C(7)–Pt(1)–N(1)	81.25(9)	C(7)–Pt(1)–O(1)	162.73(8)
N(1)–Pt(1)–O(1)	93.23(7)	C(7)–Pt(1)–P(1)	100.40(7)
N(1)–Pt(1)–P(1)	173.36(6)	O(1)–Pt(1)–P(1)	86.97(5)
C(24)–O(2)–H(1)	117(2)	C(43)–Pt(2)–N(2)	81.25(9)
C(43)–Pt(2)–O(3)	163.46(8)	N(2)–Pt(2)–O(3)	95.67(7)
C(43)–Pt(2)–P(2)	100.82(7)	N(2)–Pt(2)–P(2)	169.47(6)
O(3)–Pt(2)–P(2)	85.19(5)		

Table S8. Selection of bond lengths (Å) and angles (º) for **10**.

Pt(1)–C(7)	1.979(4)	Pt(1)–N(1)	2.092(3)
Pt(1)–O(1)	2.160(2)	Pt(1)–P(1)	2.2218(9)
O(1)–C(19)	1.358(4)	O(1)–H(1O)	0.827(10)
Pt(2)–C(42)	1.986(4)	Pt(2)–N(2)	2.092(4)
Pt(2)–O(2)	2.188(3)	Pt(2)–P(2)	2.2108(10)
C(7)–Pt(1)–N(1)	80.58(15)	C(7)–Pt(1)–O(1)	166.91(13)
N(1)–Pt(1)–O(1)	95.72(11)	C(7)–Pt(1)–P(1)	102.68(12)
N(1)–Pt(1)–P(1)	173.23(9)	O(1)–Pt(1)–P(1)	82.37(7)
C(42)–Pt(2)–N(2)	79.86(18)	C(42)–Pt(2)–O(2)	171.35(15)
N(2)–Pt(2)–O(2)	99.37(15)	C(42)–Pt(2)–P(2)	99.67(13)
N(2)–Pt(2)–P(2)	175.62(11)	O(2)–Pt(2)–P(2)	81.75(9)

Table S9. Bond lengths (Å) and angles (º) involved in the hydrogen bonding found in the structures of **9** and **10**.

	**9**	**10**
O–H	0.84(3)	0.83(6), 0.83(12)
O···O'	2.461(2)	2.447(4), 2,472(5)
O'···H	1.66(3)	1.63(6), 1.64(12)
O–H···O'	160(3)	170(6), 176(11)
C–O–H	117(2)	110(4), 107(7)

4. Computational details

Density Functional Theory (DFT) calculations were carried out with the Gaussian 16 suite of programs,[5] using the BP86 density functional[6-7] together with Grimme's D3 dispersion correction.[8] The ECP-60-mwb pseudopotential was used for platinum and gold,[9] the ECP-28-mwb pseudopotential was used for silver,[9] and the 6-31G(d) basis set was used for all other atoms.[10-11] Geometry optimizations were performed without any symmetry constraint in CH_2Cl_2, by using the solvation model based on density (SMD).[12] Stationary points were characterized by calculating the Hessian matrix analytically to confirm that all species are minima (no imaginary frequencies) or transition states (one single imaginary frequency) on the potential energy surface. Atomic coordinates (x, y, z) for the optimized structures are included in a separate file (DFTstructures.xyz).

5. References

[1] M. Baya, Ú. Belío, I. Fernández, S. Fuertes, A. Martín, *Angew. Chem. Int. Ed.* **2016**, *55*, 6978-6982.

[2] CrysAlis RED, CCD camera data reduction program, Rigaku Oxford Diffraction, Oxford, UK, **2019**.

[3] G. Sheldrick, *Acta Crystallogr. Sect. C* **2015**, *71*, 3-8.

[4] P. Van der Sluis, A. L. Spek, *Acta Cryst.* **1990**, *A46*, 194-201.

[5] M. J. Frisch, G. W. Trucks, H. B. Schlegel, G. E. Scuseria, M. A. Robb, J. R. Cheeseman, G. Scalmani, V. Barone, G. A. Petersson, H. Nakatsuji, X. Li, M. Caricato, A. V. Marenich, J. Bloino, B. G. Janesko, R. Gomperts, B. Mennucci, H. P. Hratchian, J. V. Ortiz, A. F. Izmaylov, J. L. Sonnenberg, Williams, F. Ding, F. Lipparini, F. Egidi, J. Goings, B. Peng, A. Petrone, T. Henderson, D. Ranasinghe, V. G. Zakrzewski, J. Gao, N. Rega, G. Zheng, W. Liang, M. Hada, M. Ehara, K. Toyota, R. Fukuda, J. Hasegawa, M. Ishida, T. Nakajima, Y. Honda, O. Kitao, H. Nakai, T. Vreven, K. Throssell, J. A. Montgomery Jr., J. E. Peralta, F. Ogliaro, M. J. Bearpark, J. J. Heyd, E. N. Brothers, K. N. Kudin, V. N. Staroverov, T. A. Keith, R. Kobayashi, J. Normand, K. Raghavachari, A. P. Rendell, J. C. Burant, S. S. Iyengar, J. Tomasi, M. Cossi, J. M. Millam, M. Klene, C. Adamo, R. Cammi, J. W. Ochterski, R. L. Martin, K. Morokuma, O. Farkas, J. B. Foresman, D. J. Fox, Gaussian 16 Rev. C.01, Wallingford, CT, **2016**.

[6] A. D. Becke, *Phys. Rev. A* **1988**, *38*, 3098-3100.

[7] J. P. Perdew, *Phys. Rev. B* **1986**, *33*, 8822-8824.

[8] S. Grimme, J. Antony, S. Ehrlich, H. Krieg, *J. Chem. Phys.* **2010**, *132*, 154104.

[9] D. Andrae, U. Häußermann, M. Dolg, H. Stoll, H. Preuß, *Theor. Chim. Acta* **1990**, *77*, 123-141.

[10] R. Ditchfield, W. J. Hehre, J. A. Pople, *J. Chem. Phys.* **1971**, *54*, 724-728.

[11] P. C. Hariharan, J. A. Pople, *Theor. Chim. Acta* **1973**, *28*, 213-222.

[12] A. V. Marenich, C. J. Cramer, D. G. Truhlar, *J. Phys. Chem. B* **2009**, *113*, 6378-6396.
